# *N*-Biphenyl Pyrrolinones and Dibenzofurans as RNA-Binding Protein LIN28 Inhibitors Disrupting the LIN28–*Let-7* Interaction

**DOI:** 10.1021/acsmedchemlett.3c00341

**Published:** 2023-11-14

**Authors:** Lydia Borgelt, Lisa Hohnen, Jakob S. Pallesen, Pascal Hommen, Georg L. Goebel, Francesco Bosica, Yang Liu, Gavin O’Mahony, Peng Wu

**Affiliations:** † Chemical Genomics Centre, Max Planck Institute of Molecular Physiology, Otto-Hahn Str. 15, Dortmund 44227, Germany; ‡ Department of Chemical Biology, Max Planck Institute of Molecular Physiology, Otto-Hahn Str. 11, Dortmund 44227, Germany; § Faculty of Chemistry and Chemical Biology, TU Dortmund University, Otto-Hahn Str. 6, Dortmund 44227, Germany; ∥ Faculty of Chemistry and Biochemistry, Ruhr-University Bochum, Universitätsstr. 150, Bochum 44801, Germany; ¶ Medicinal Chemistry, Research and Early Development, Cardiovascular, Renal and Metabolism, BioPharmaceuticals R&D, AstraZeneca, SE-431 83 Mölndal, Sweden

**Keywords:** Pyrrolinones, LIN28 inhibitors, RNA-binding protein, Structure−activity relationship

## Abstract

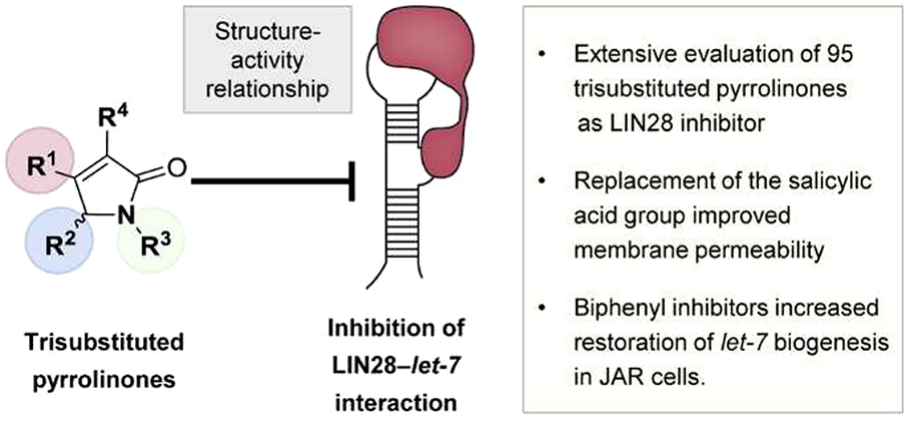

The RNA-binding protein LIN28 is a regulator of miRNA *let-7* biogenesis. Inhibitors of LIN28 are highly sought after given the central role that LIN28 plays in tumorigenesis and development of cancer stem cells as well as LIN28’s association with poor clinical prognosis. Although LIN28 inhibitors of different scaffolds have been reported, the potential of most LIN28 inhibiting small molecules was not fully explored since very limited structure–activity relationship (SAR) studies have been performed. We previously identified trisubstituted pyrrolinones as a new class of LIN28 inhibitors disrupting the LIN28–*let-7* interaction. Here, we performed extensive SAR by evaluating 95 small molecules and identified new trisubstituted pyrrolinones featuring either an *N*-biphenyl or *N*-dibenzofuran substituent, overthrowing the existing conclusion that a salicylic acid moiety is indispensable for activity. Exchange of the negatively charged salicylic acid moiety in LIN28 inhibitors with a heterocyclic substituent is beneficial for membrane permeability, leading to increased activity in a cellular assay, and will potentially reduce toxicity.

RNA-binding proteins (RBPs) are a large protein class with more than 1500 members involved in all stages of RNA metabolism, regulation, and function. RBPs post-transcriptionally regulate not only coding mRNAs but also noncoding RNAs such as lncRNAs and miRNAs.^1,2^ Due to their involvement in versatile cellular processes, dysregulation of which often leads to human diseases, RBPs are emerging as new targets in biological studies to probe cellular regulatory networks and development of new therapeutic agents.^3−5^ One of the first discovered as well as most extensively studied RNA-binding proteins is the miRNA-binding protein LIN28. It regulates stem-cell differentiation by forming a bistable switch together with its interaction partner miRNA *let-7*, via inhibition of *let-7* biogenesis by LIN28 and the repression of LIN28 mRNA expression by mature *let-7*.^6^ High expression levels of LIN28 are usually observed in undifferentiated, pluripotent cells in early development, while high *let-7* expression is associated with cell differentiation.^7,8^ The LIN28 protein comprises two RNA binding domains, an *N*-terminal cold shock domain (CSD) and a *C*-terminal zinc knuckle domain (ZKD) connected by a flexible linker.^9^ The CSD mainly recognizes a (U)GAU consensus sequence while the ZKD binds to RNA with GGAG motifs.^10^ In humans, the protein LIN28 exists in two isoforms, LIN28A and LIN28B. The latter was mainly observed in the nucleolus, while LIN28A mainly acts in the cytoplasm.^6^ Both isoforms inhibit *let-7* biogenesis by binding to primary and precursor miRNA hairpin loops prior to processing and thereby inhibiting RNA cleavage by Drosha and Dicer (Figure 1A). LIN28A is additionally able to recruit terminal uridylyltransferases targeting its interaction partner pre-*let-7* for degradation.^11^ Inhibition of LIN28 by small molecules could disrupt its interaction with *let-7* and thus restore *let-7* miRNA biogenesis (Figure 1B). Mature *let-7* is bound by Ago proteins and incorporated into the RNA-induced silencing complex (RISC) in which it acts by binding to the 3′-untranslated regions of mRNAs, suppressing the translation of mRNAs of oncogenic proteins, such as HMGA2, MYC and RAS.^12,13^ Of note, LIN28 overexpression is considered a biomarker for cancer stem cells and is associated with poor prognosis in cancer.^14,15^ Further, LIN28 was reported to be involved in the glucose metabolism of cancer cells.^16^ Therefore, LIN28 inhibitors are promising candidates for cancer therapy.

**Figure 1:**
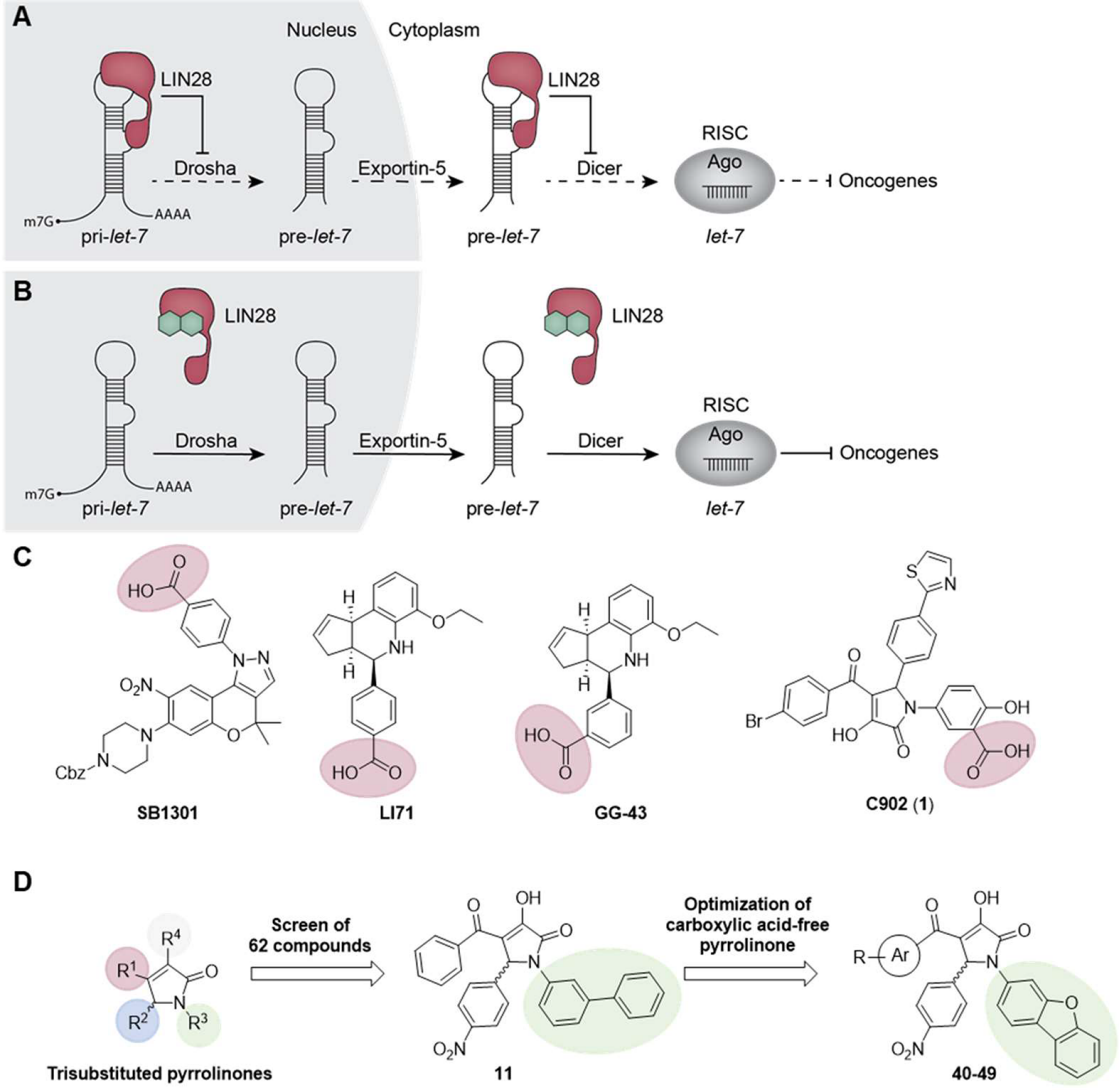
(A) Simplified illustration of the LIN28–*let-7* biogenesis pathway. (B) Inhibition of the LIN28–*let-7* interaction via small molecules as a promising anticancer strategy. (C) Reported LIN28 CSD inhibitors with a carboxylic acid. (D) Three generations of trisubstituted pyrrolinones were evaluated in this extensive SAR study as LIN28 inhibitors. Ago, protein argonaute; RISC, RNA-induced silencing complex.

Small-molecule LIN28 inhibitors of diverse scaffolds have been discovered via different screening approaches, mainly based on biochemical assays.^17−27^ However, most identified molecules lack potent cellular activity, and only limited structure–activity relationship (SAR) evaluation has been performed for a limited selection of LIN28-inhibiting small molecules. Notably, among the most potent reported inhibitors, the presence of a carboxylic acid is crucial for LIN28 inhibitory activity against the CSD (Figure 1C). Associated with our efforts in developing LIN28 inhibitors,^26−29^ we previously identified trisubstituted pyrrolinones as a new series of LIN28 inhibitors, represented by the compound C902 (**1**) binding to the cold shock domain. Initial SAR investigation surrounding **1** showed the importance of an *N*-salicylic acid substituent while other functional groups substituted on the pyrrolinone core scaffold allowed for modification to variable extents.^26^ In this work, we tested the activity for a total of 95 small-molecule analogues of compound **1** and extensively evaluated the SAR of the trisubstituted pyrrolinones as LIN28 inhibitors (Figure 1D). The first-generation small molecules are mostly salicylic-acid-containing compounds based on the reported inhibitor **1**. In contrast to our previous finding on the essential salicyclic acid group, one pyrrolinone without the salicylic acid substituent but with a biphenyl group retained activity and caught our attention. The absence of the carboxylic acid is probably associated with improved membrane permeability and less metabolic toxicity, thus the pyrrolinones harboring the biphenyl group instead of the salicylic acid group were the focus in the second-generation analogues. A series of small molecules in which the biphenyl group was replaced by a dibenzofuran group were included in this study as the third-generation analogues with improved activity. To note, in this study we also evaluated a fourth-generation analogue with a spirocyclic pyrrolinone scaffold, which did not show inhibition against the LIN28–*let-7* interaction.

We identified the trisubstituted pyrrolinone inhibitors by a fluorescence polarization assay measuring the disruption of the LIN28–*let-7* interaction. An initial SAR study with compounds bearing varied substituents on the *N*-phenyl group showed the importance of the salicylic acid moiety at this position. Given the bioavailability associated with the salicylic acid, in this study, we aimed to identify potent pyrrolinones without the salicylic acid group by performing an extensive SAR analysis involving 95 compounds. The compounds featured a variety of modifications at the 1-, 3-, 4- and 5-positions of the trisubstituted pyrrolinone scaffold (Figure 1D).

First, we screened a total of 60 compounds, including compound **2** that are all analogues of **1** (Tables S1–S4) using an electrophoretic mobility shift assay (EMSA). Compound **2** is a trisubstituted pyrrolinone harboring the crucial salicylic acid moiety of **1**, a nitrophenyl residue at the 5-position and a benzoyl substituent at the 4-position. Compound **2**, as well as the series of trisubstituted pyrrolinones, was previously reported as a stabilizer of the protein–protein interaction (PPI) between 14-3-3 protein and the plasma membrane protein H^+^-ATPase 2 (PMA2) of plants.^30−32^ The EMSA assay separates the protein–RNA complex of LIN28 and *let-7* from unbound *let-7* in nondenaturing electrophoresis. The RNA was detected by its fluorophore label, allowing a visual readout and a robust throughput. Although compound **2** was not active in disrupting the LIN28–*let-7* interaction, some of its derivatives showed varied activity against LIN28 indicating a different SAR for both proteins, as observed in our previous SAR study.^26^

A total of 11 compounds among the 60 pyrrolinones showed at least 60% inhibition measured in EMSA at a single concentration of 75 μM (Figures 2, S1, and S2) Generally, single modifications at each of the three substituents of the trisubstituted pyrrolinones were sufficient to render the compounds active. Among the 16 compounds with modified 4-position substituent of the pyrrolinone, three compounds **3**, **4** and **5** showed a minimum inhibitory activity of 60%. Interestingly, **5** lacked the carbonyl group that was present in **1** and most other active compounds. Instead, a thiazol-2-yl substituent was directly attached at position 4 of the pyrrolinone core. The three active inhibitors together with the inactive compounds revealed that the presence of the salicylic acid moiety at position 1 is not sufficient for the inhibition of LIN28. This observation was further underlined by molecules with modifications at the 5-position since not all such small molecules were able to inhibit LIN28 despite bearing the salicylic acid moiety. Active molecules harbored nitrogen-containing heterocycles (**6**, **7**, **8**, **9**) or a 4-aminophenyl group (**10**), possibly involved in hydrogen bond formation with the LIN28 protein. Modifications of the 1-position revealed that the general trend that modifications of the salicylic acid residue led to a loss in activity.^26^ It is noteworthy that all investigated pyrrolinones have a stereocenter at the 5-position of the core scaffold. For the testing of the 60 pyrrolinones, racemic mixtures of the compounds were used mostly, except for molecules **16** and **17**, **18** and **19**, **20** and **21**, and **22** and **23** that were enantiomerically pure isomers. However, none of these aforementioned enantiomerically pure molecules were active.

**Figure 2:**
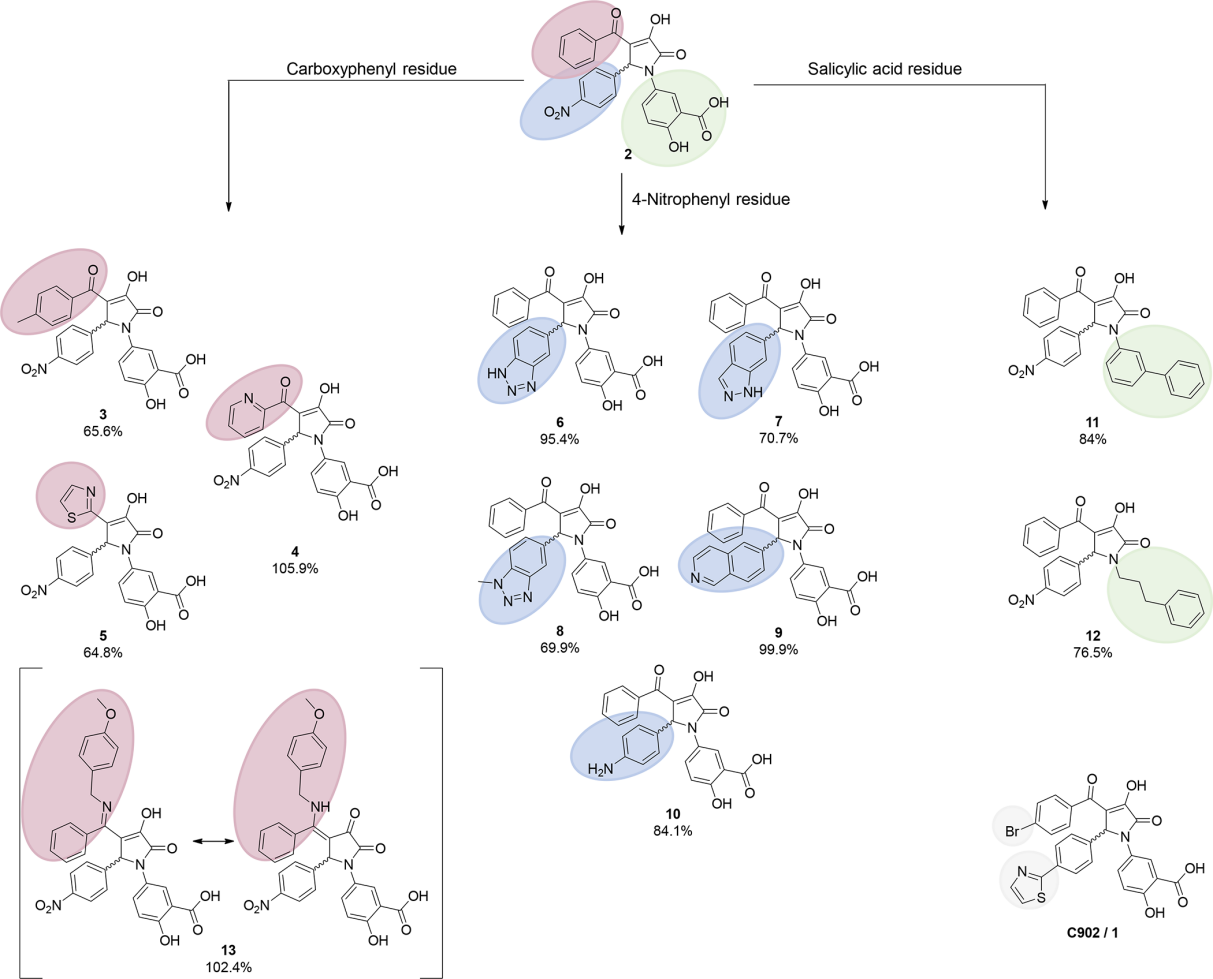
Summary of the 11 pyrrolinones (compounds **3**–**13**) that showed at least 60% inhibition measured in the EMSA at 75 μM. The structure of our previously reported LIN28 inhibitor **1** is shown in the bottom right corner for comparison. Structural variations at the benzoyl 4-nitrophenyl, 5-phenyl, and salicylic acid substituents are highlighted in red, blue, and green, respectively.

Of particular interest were two molecules **11** and **12** without the salicylic acid moiety, which showed inhibitory activity of 84% and 77%, respectively. Modification of the carbonyl on the benzoyl substituent led to compound **13**, an active compound stabilized by an internal hydrogen bond. Further rigidification via the formation of a bicyclic core led to pyrrolopyrazolones **14** and **15** that were inactive. Active molecules were further investigated in dose–response EMSA (Figure 3). All 11 pyrrolinones shown in Figure 2 inhibited the formation of the LIN28–*let-7* interaction in a concentration-dependent manner. Of the 11 active compounds, compound **8** seemed to be the least active (IC_50_: ∼90 μM), matching with the observation that compound **8** was among the molecules with the lowest potency in the initial screen. Compound **9**, which has an isoquinolin-6-yl substituent at the 5-position of the pyrrolinone, was the most active small molecule (IC_50_ ∼ 5 μM). Compounds **10** and **12** showed activities with IC_50_ between 5 and 15 μM. Compounds **6** and **4** were slightly less active with IC_50_ values of ∼20 and ∼19 μM, respectively.

**Figure 3:**
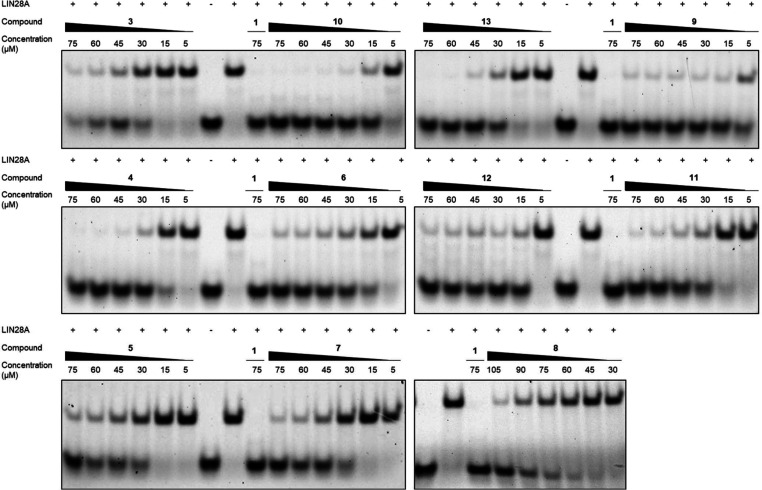
Concentration-dependent evaluation for the 11 pyrrolinone inhibitors that disrupted LIN28–*let-7* complex formation in EMSA.

Exchange of the salicylic acid group with an alternative functional group without carboxylic acid is beneficial for cellular permeability of small-molecule drugs and probes. The lack of the negatively charged salicylic acid moiety could facilitate passive diffusion across membranes and thus increase the cellular bioavailability of small-molecules. Further, carboxylic acids were reported to potentially have toxic metabolic products.^33^ Therefore, the pyrrolinone **11** without the salicylic acid moiety in the 1-position but with retained LIN28 inhibitory activity caught our attention for the following modifications and evaluations. The carboxylic acid moiety of **1** was predicted to form a hydrogen bond with lysine 98 of the LIN28 CSD and the backbone of alanine 101. In comparison, the biphenyl group of compound **11** could be involved in π–cation interactions instead.

Pyrrolinone **11** was then evaluated for its direct binding to LIN28 in a biolayer interferometry measurement, in which it showed concentration-dependent binding to the LIN28 CSD (Figure 4A). The biolayer interferometry result hinted an inhibition mechanism driven by binding to the CSD, resembling the inhibition mechanism of compound **1**.^26^ Subsequently, the (*S*)- and (*R*)-enantiomers of **11**, namely **11S** and **11R**, respectively, were tested for their inhibitory potency against LIN28 (Figure 4B–D). Both enantiomers induced an increase in the thermal stability of the LIN28 CSD by ∼1.9 °C when incubated with protein at 75 μM. In this measurement monitoring direct binding to LIN28 CSD, the isomers of **11** were even more active than compounds **4**, **6**, **9**, and **10**, some of the most active small molecules in the dose–response EMSA. In comparison, compound **8**, the least active compound of those evaluated in concentration-dependent EMSA, induced a shift of less than 0.5 °C in the thermal shift assay. Dose–response EMSA of the two enantiomers of **11** revealed that the (*R*)-enantiomer was slightly more active than the (*S*)-enantiomer with IC_50_ values of 24 and 37 μM, respectively. The racemic mixture of both enantiomers exhibited an IC_50_ value of 27 μM, indicating that both enantiomers are involved in LIN28 inhibition, while the (*R*)-enantiomer seems to have a slightly favorable geometry. Compound **11** as a racemic mixture was further tested in the JAR cells for the ability to perturb levels of mature *let-7i* and *let-7d* miRNAs. Biogenesis of the two *let-7* miRNAs was affected by compound **11** treatment in a concentration-dependent manner (Figure 4E). A 2- to 3-fold increase in inducing the mature miRNA level in comparison with that of compound **1** was observed, indicating that the exchange of the salicylic acid moiety led to increased cellular activity.

**Figure 4:**
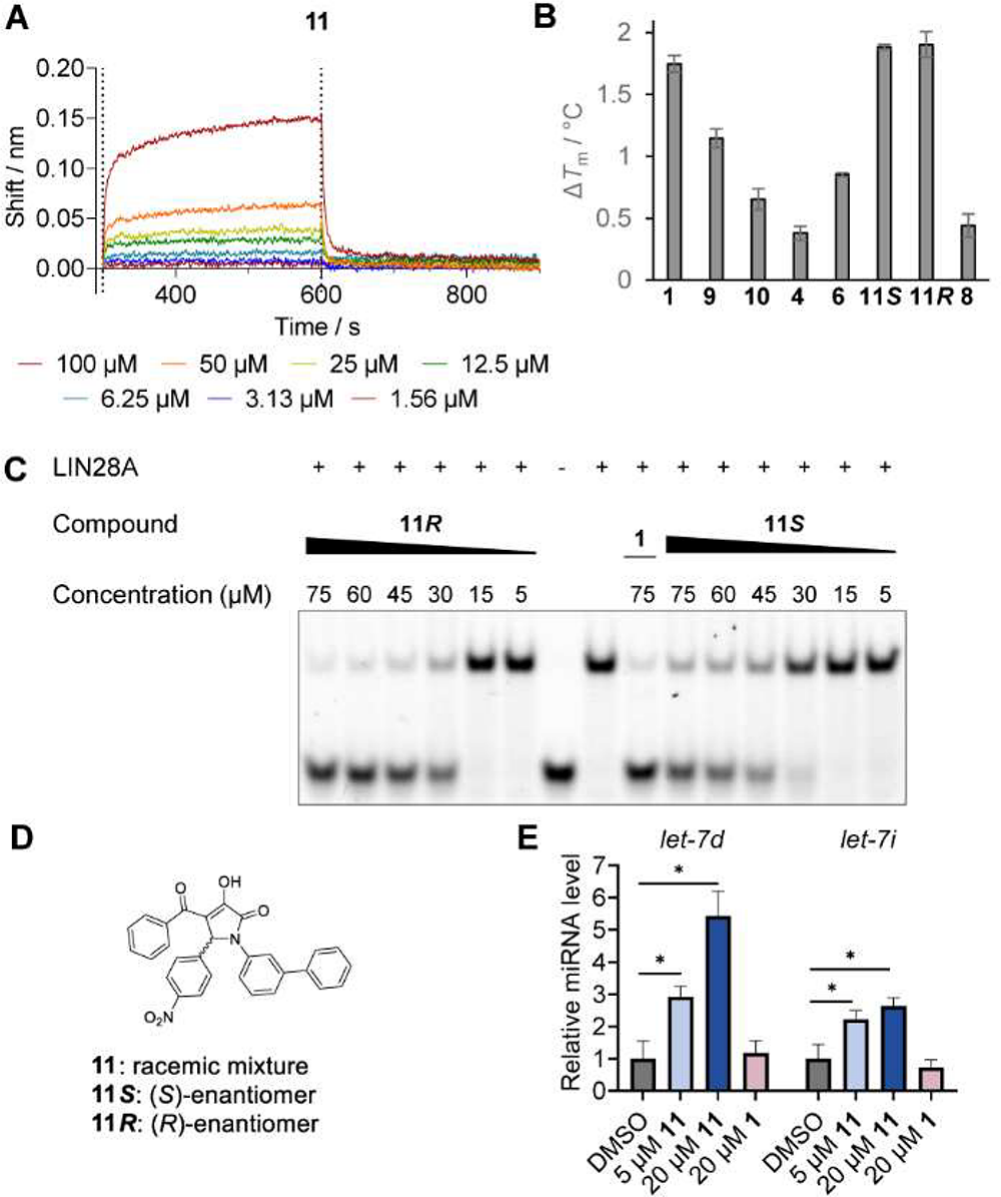
(A) Concentration-dependent biolayer interferometry of **11**. (B) Melting temperature of the LIN28 CSD treated with 75 μM pyrrolinones measured by nanoDSF. (C) Concentration-dependent inhibition of the LIN28–*let-7* interaction in EMSA for the enantiomers **11***R* and **11***S* of the biphenyl pyrrolinone **11**. (D) Chemical structures of **11**, **11***S*, and **11***R*. (E) Relative mature *let-7d* and *let-7i* levels in JAR cells after treatment with compounds **11** and **1**; error bars indicate the standard deviation.

Furthermore, we demonstrated that in addition to the disruption of the protein–RNA interaction between LIN28A and *let-7*, compound **11** also inhibited the LIN28B–*let-7* interaction in a concentration-dependent manner, as shown in EMSA (Figure S3).

The potential binding mode for compound **11** with the CSD of LIN28 was probed by a molecular docking analysis, which indicated the formation of a salt bridge between the nitro group of **11** and K102 of LIN28 CSD, as well as extensive π–cation and π–π stacking interactions involving the aromatic moieties of **11** and LIN28 residues K102, K78 and F73 (Figure S4).

A total of 16 analogues based on the biphenylpyrrolinone **11** with different cyclic substituents in the 4-position of the pyrrolinone core were then synthesized and assayed in the single-dose EMSA at 75 μM concentration (Figures 5 and S1). Several functional groups which were not included in the initial 60 compounds, such as furan-2-yl, cycloalkyls or 2-fluorophenyl, were incorporated in the 16 compounds collection. In general, pyrrolinones with cycloalkyl substituents (**24**–**26**) were less active than compounds with aromatic heterocycles, and certain modifications of the phenyl ring led to complete loss of activity (**27**–**29**). It seemed that a halogen or methoxy substituent at ortho- or para-position (**30**–**33**), but not disubstituted at both para- and meta-position (**34**, **29**), were favorable for activity. Compound **33** with a 2-fluorophenyl group showed LIN28 inhibition higher than that of compound **11**. As an alternative to phenyl modification with hydrogen bond acceptor substituents, aromatic five-membered heterocycles with hydrogen bond acceptors such as furan-2-yl and *N*-methyl-pyrrole led to molecules **35**–**37** with equivalent activities. The observations from the second-generation pyrrolinones matched the results from the initial screening in which compound **4** with a pyridine-2-yl substituent and compound **5** with a thiazole-2-yl group were active, which can probably be attributed to the additional hydrogen bond acceptors in the modified substituents. All compounds with an activity above 60% were evaluated in a concentration-dependent assay (Figure S2). Three of the four molecules with screening activity above 80% exhibited IC_50_ below 30 μM while the molecules with less than 80% activity had IC_50_ values above 30 μM, with the exception of compound **35** that showed the highest activity in the single-dose experiment but had an IC_50_ of ∼45 μM.

**Figure 5:**
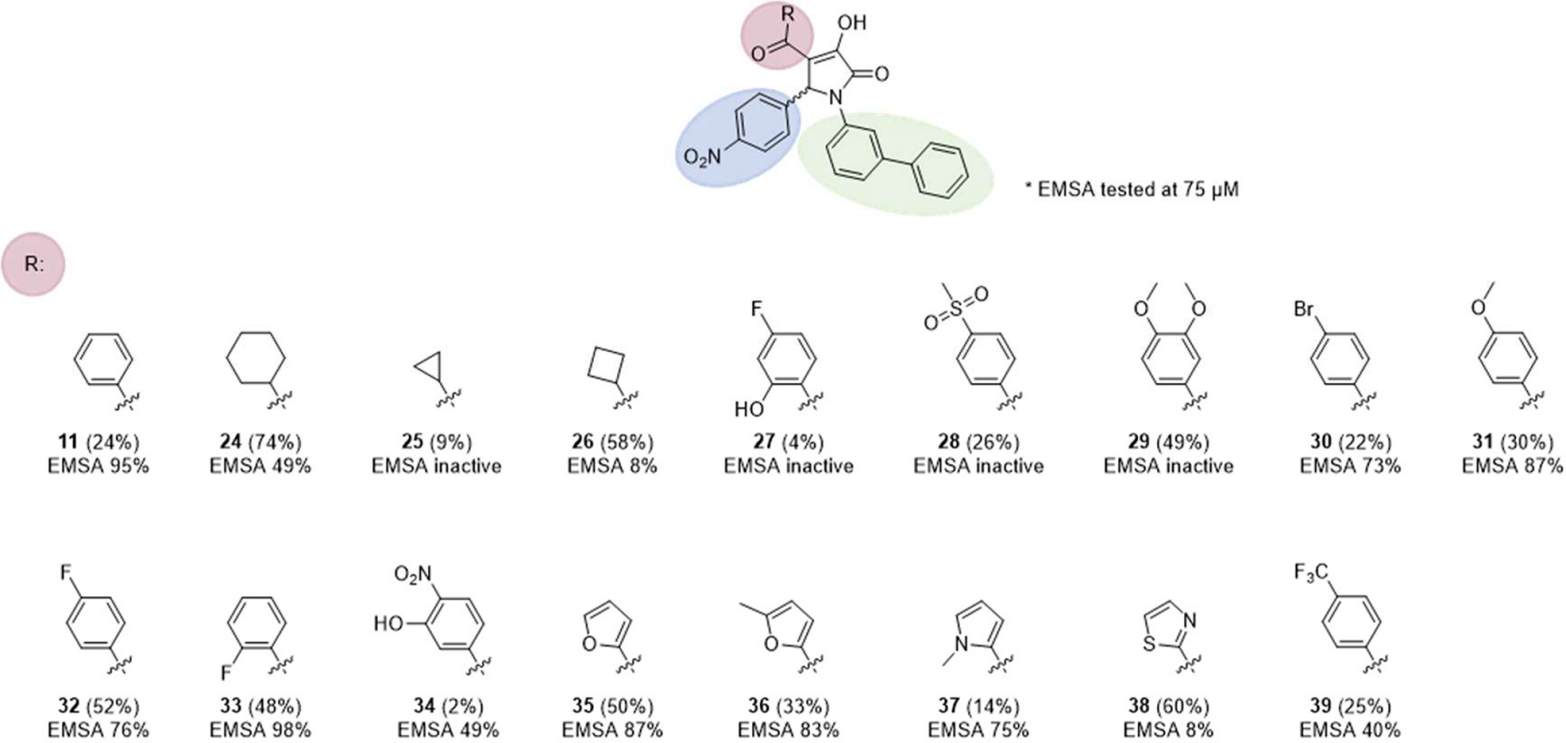
Synthesized *N*-biphenyl-substituted pyrrolinones and their tested LIN28–*let-7* inhibitory activity in EMSA.

A third-generation of 10 pyrrolinones was then synthesized via modifications on the *N*-biphenyl group to add another possible hydrogen bond acceptor and further rigidify the scaffold. The idea is to reduce conformational entropy penalty upon the small-molecule binding to macromolecular targets. Specifically, the biphenyl group was fused via a furanyl moiety to form a tricyclic dibenzofuran substituent. Two different linkage positions of the dibenzofuranyl were evaluated with five distinct substituents at the 4-position of the pyrrolinone core. The molecules were tested for their ability to inhibit the interaction of LIN28 and *let-7* (Figures 6A and S1).

**Figure 6:**
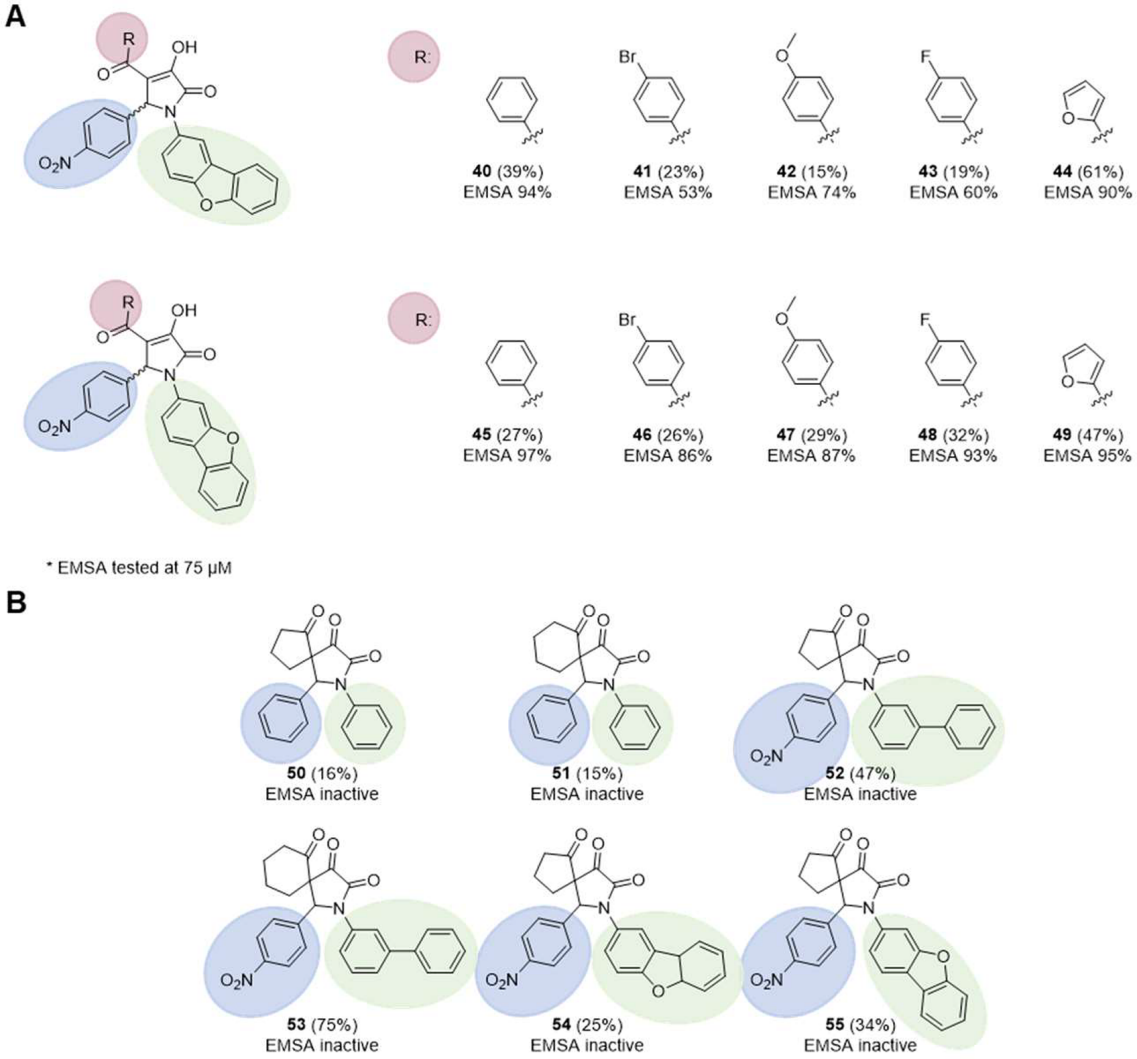
(A) Synthesized *N*-dibenzofuran-substituted pyrrolinones and their tested LIN28–*let-7* inhibitory activity in EMSA. (B) Synthesized spirocyclic pyrrolinones did not show activity in EMSA.

All 10 dibenzofurans inhibited LIN28 in the single-dose EMSA, with generally higher activities in the case of the dibenzofuran-3-yl- instead of the dibenzofuran-2-yl-pyrrolinones. Similar to the second-generation biphenyl pyrrolinones, the unsubtituted benzoyl group in the 4-position of the pyrrolinone led to the highest activity against LIN28. All that dibenzofurans showed more than 60% inhibition were further tested in the concentration-dependent EMSA (Figure S2), which revealed that compounds **45** and **49**, both with dibenzofuran-3-yl at the 1-position but with a 4-phenyl or a 4-furan-2-yl at the 4-position, respectively, exhibited the best inhibitory activities among the evaluated molecules.

A final diversification we performed was to investigate the formation of a spirocyclic pyrrolinone scaffold (Figure 6B). Spirocyclization rigidifies scaffolds while simultaneously adding three-dimensionality, potentially increasing the binding affinity and potency of the molecules.^34,35^ Six different spirocyclic pyrrolinones harboring either phenyl-, biphenyl-, or dibenzofuranyl substituents at the 1-position of the pyrrolinone core were evaluated (Figures 6B and S1). However, none of the spirocyclic pyrrolinones was able to disrupt the LIN28–*let-7* interaction, and thus, the spirocyclization direction was not pursued further.

In summary, we performed extensive structural modifications based on the LIN28-inhibiting trisubstituted pyrrolinone scaffold via the evaluation of a collection of 95 analogues. Modifications of the trisubstituted pyrrolinones at the 1-, 3-, 4- and 5-positions of the core were tested for their impact on inhibition against the interaction between LIN28 and *let-7*. From our study, a salicylic acid (e.g., compound **1**) or a dibenzofuran-3-yl substituent in the 1-position, furan-2-yl (**49**) or picolinoyl (**4**) in the 4-position, and isoquinolin-6-yl (**9**) in the 5-position of the pyrrolinone were shown to be the components required to achieve the optimal inhibitory activity for the pyrrolinone inhibitors. Contrary to the previous observations from a narrower SAR evaluation,^26^ the salicylic acid moiety was proven to be not indispensable for LIN28-inhibitory activity in this study. The salicylic acid group can be replaced by the biphenyl or dibenzofuranyl substituents, which were evaluated as the second- and third-generation pyrrolinones, respectively. The lack of the charged carboxylic acid theoretically facilitates passive diffusion through cellular membranes and thus increases the bioavailability of the pyrrolinones.^33^ Subsequently, treatment with the biphenyl inhibitor **11** led to an increased fold change in inducing the mature *let-7* level in LIN28-overexpressing JAR cells.

The trisubstituted pyrrolines were reported to be able to chelate with Mg^2+^-ions via the 3-hydroxy group and the 4-carbonyl moiety,^30^ suggesting that the LIN28 inhibitors described in this study could potentially act as metal chelators. Our in-house data invalidated the possibility that the observed LIN28 inhibition was due to metal chelating activity. Specifically, the reported metal-chelating compounds **22** and **23** were inactive against LIN28 in our assays; while in comparison, compound **13** that was reported to function in a metal-independent manner^30^ was active against the LIN28–*let-7* interaction. Furthermore, compound **5** that lacks the chelating 4-carbonyl moiety was active in our assays. In conclusion, the evaluated trisubstituted pyrrolinones probably functioned via a metal-independent binding mechanism as LIN28 inhibitors. This work represented the most extensive structural modifications performed so far for small molecules targeting the LIN28–*let-7* interaction and thereby paves the way for the development of next-generation LIN28 inhibitors.

## Methods

No unexpected or unusually high safety hazards were encountered.

### Purification of LIN28

Residues 16–187 of the LIN28A protein, residues 16–126 of the LIN28A cold shock domain, or residues 24–111 of the LIN28B cold shock domain were subcloned into the pET19a vector or pMAL vector. The plasmids were transformed to *Escherichia coli BL21*(DE3) for expression. Protein was produced at 18 °C for 18 h after induction with 300 μM IPTG. Cells were harvested and lysed in buffer containing 50 mM NaH_2_PO_4_, pH 7.5, 300 mM NaCl, 0.1 mM PMSF, 1% Triton-X100, SIGMAFAST Protease Inhibitor Cocktail (Sigma-Aldrich) and 10 μg/mL DNase I by sonication. Then, the suspension was centrifuged for 60 min at 4 °C and 60000*g*. The supernatant was applied on an immobilized nickel affinity chromatography column (HisTrap, GE Healthcare) equilibrated with buffer A (50 mM NaH_2_PO_4_, pH 8.0, 300 mM NaCl, and 5% glycerol). Protein was eluted using a gradient with up to 0.5 M imidazole before cleavage of the affinity tag using TEV protease overnight at 4 °C dialyzing against buffer A. Reverse nickel affinity chromatography was then performed before final purification by gel-filtration using a buffer containing 30 mM NaH_2_PO_4_, pH 7.5, 50 mM NaCl, 5% glycerol, and 2 mM β-mercaptoethanol and a HighLoad Superdex 75pg 16/600 column (GE Healthcare). The purified protein was then concentrated, snap-frozen in liquid nitrogen, and stored at −80 °C.

### Electrophoretic Mobility Shift Assay

Inhibition of the LIN28–*let-7* complex was evaluated by electrophoretic mobility shift assays. Mixtures containing compound at indicated concentrations, LIN28A(16–187) or LIN28B(24–111), and recombinant ribonuclease inhibitor (TaKaRa Bio) were incubated at room temperature for 2 h. The reaction buffer contained 50 mM TRIS, pH 7.5, 100 mM NaCl, 10 mM β-mercaptoethanol, 50 μM ZnCl_2_, 2% DMSO, 0.01% Tween-20 and 12% glycerol. Cy-3-fluorophore labeled RNA, (pre-E *let-7f-1*-Cy3, *Mus musculus*, GGGGUAGUGAUUUUACCCUGUUUAGGAGAU-Cy3, purchased from IDT) was added to the reaction and incubated for further 15 min. The final concentrations were as follows: 10 nM LIN28A or 200 nM LIN28B, 5 nM RNA. Of each mixture, 10 μL was analyzed on a nondenaturing PAGE (5.3% acrylamide TAE PAGE for full-length LIN28 and 10% acrylamide TAE PAGE for analysis of the CSD) prerun for 1 h without sample and run for 1 h at 220 V at 4 °C in 0.25× TAE buffer. Gels were imaged using a ChemiDoc MP instrument (Bio-Rad Laboratories). Band intensities were analyzed using ImageJ and inhibition was quantified by calculation of the ratio of free RNA fluorescence to the fluorescence of the protein–RNA complex, and normalization to free RNA and LIN28–*let-7* complex controls. The intensity ratio was analyzed by nonlinear regression fit in GraphPad Prism to determine the IC_50_ values.

## Supporting Information Available

Supplementary tables, figures and methods, general chemistry information, synthetic procedures, compound characterization data, NMR spectra, and chiral separation procedure (PDF)

## Abbreviations

BLI: biolayer interferometry
CSD: cold shock domain
EMSA: electrophoretic mobility shift assay
nanoDSF: nanodifferential scanning fluorimetry
PPI: protein–protein interaction
RBP: RNA-binding protein
RISC: RNA-induced silencing complex
SAR: structure–activity relationship
ZKD: zinc knuckle domain
