# Supplementary material for: N-Biphenyl Pyrrolinones and Dibenzofurans as RNA-Binding Protein LIN28 Inhibitors Disrupting the LIN28–Let-7 Interaction
Source: ACS Med Chem Lett. 2023 Nov 14;14(12):1707–15. doi: 10.1021/acsmedchemlett.3c00341 (PMC10726440; doi:10.1021/acsmedchemlett.3c00341)
Supplement: Supplementary file 1 — ml3c00341_si_001.pdf [file ml3c00341_si_001.pdf]

Supporting Information

N-Biphenyl Pyrrolinones and Dibenzofurans as RNA-Binding Protein LIN28 Inhibitors Disrupting the LIN28–*Let-7* Interaction

Lydia Borgelt,[†,‡,§,#] Lisa Hohnen,[†,‡,||,#] Jakob S. Pallesen,[¶,#] Pascal Hommen,[†,‡,§,#] Georg L. Goebel,[†,‡,§,#] Francesco Bosica,[¶] Yang Liu,[†,‡,§] Gavin O'Mahony,[¶,*] and Peng Wu[†,‡,*]

[†]Chemical Genomics Centre, Max Planck Institute of Molecular Physiology, Otto-Hahn Str. 15, Dortmund 44227, Germany

[‡]Department of Chemical Biology, Max Planck Institute of Molecular Physiology, Otto-Hahn Str. 11 Dortmund 44227, Germany

[§]Faculty of Chemistry and Chemical Biology, TU Dortmund University, Otto-Hahn Str. 6, Dortmund 44227, Germany

[||]Faculty of Chemistry and Biochemistry, Ruhr-University Bochum, Universitätsstr. 150, Bochum 44801, Germany

[¶]Cardiovascular and Metabolic Diseases, Innovative Medicines and Early Development Biotech Unit, AstraZeneca Gothenburg, Pepparedsleden 1, SE-431 83 Mölndal, Sweden

* Correspondence to: peng.wu@mpi-dortmund.mpg.de; gavin.omahony@astrazeneca.com

Contents

Supplementary Tables....S3

Supplementary Figures S9

Supplementary Methods S13

General Chemistry Information....S13

General Procedure for the Doebner Condensation S166

General Procedure for Butanoate Synthesis S38

NMR Spectra S43

Chiral separation....S72

Reference S75

Supplementary Tables

Table S1. Tested compounds (R^3, R^4 variations)

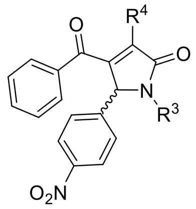

Index	Cpd.	R^3	R^4	Isomerism
1	**56**	3-carboxy-4-hydroxyphenyl	4-(methyl-benzyl)amine	racemic mixture
2	**57**	3-phenylethyl	hydroxy	racemic mixture
3	**11**	biphenyl	hydroxy	racemic mixture
4	**58**	phenyl	hydroxy	racemic mixture
5	**59**	phenyl	amino	racemic mixture
6	**60**	phenyl	ethylamino	racemic mixture
7	**61**	2-chlorophenyl	hydroxy	racemic mixture
8	**62**	2-carboxyphenyl	hydroxy	racemic mixture
9	**63**	3-carbamoyl-phenyl	hydroxy	racemic mixture
10	**64**	3-sulfamoyl-phenyl	hydroxy	racemic mixture
11	**65**	3-trifluoro-methylphenyl	hydroxy	racemic mixture
12	**66**	4-trifluoro-methylphenyl	hydroxy	racemic mixture
13	**67**	4-nitrophenyl	hydroxy	racemic mixture
14	**68**	4-cyanophenyl	hydroxy	racemic mixture
15	**69**	4-hydroxyphenyl	hydroxy	racemic mixture
16	**70**	2-phenoxyethyl	hydroxy	racemic mixture
17	**12**	3-phenylpropyl	hydroxy	racemic mixture
18	**71**	benzyl	hydroxy	racemic mixture
19	**72**	3-carboxy-4-hydroxyphenyl	amino	racemic mixture
20	**73**	4-carboxy-pyridin-2-yl	hydroxy	racemic mixture
21	**74**	3-carboxy-methylphenyl	hydroxy	racemic mixture
22	**2(*R/S*)**	3-carboxy-4-hydroxyphenyl	hydroxy	isomer (*R*) or (*S*)
23	**2(*S/R*)**	3-carboxy-4-hydroxyphenyl	hydroxy	isomer (*S*) or (*R*)

24	**11*S***	biphenyl	hydroxy	(*S*)-isomer
25	**11*R***	biphenyl	hydroxy	(*R*)-isomer
26	**20**	3-nitrilophenyl	hydroxy	(*S*)-isomer
27	**21**	3-nitrilophenyl	hydroxy	(*R*)-isomer
28	**22**	3-carboxy-4-hydroxyphenyl	NH_2	(*R*)-isomer

Table S2. Tested compounds (R^1 variations)

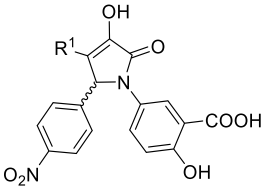

Index	Cpd.	R^1	Isomerism
1	**3**	4-methyl-benzoyl	racemic mixture
2	**23**	imino(phenyl)	(*R*)-isomer
3	**75**	4-methoxy-benzyl	racemic mixture
4	**76**	2-bromobenzoyl	racemic mixture
5	**77**	2-chlorobenzoyl	racemic mixture
6	**78**	4-chlorobenzoyl	racemic mixture
7	**79**	3-(difluoromethoxy)benzoyl	racemic mixture
8	**80**	benzyl	racemic mixture
9	**4**	picolinoyl	racemic mixture
10	**81**	Pyrazin-2-yl	racemic mixture
11	**5**	thiazol-2-yl	racemic mixture
12	**16**	4-chlorobenzoyl	(*R*)-isomer
13	**17**	4-chlorobenzoyl	(*S*)-isomer
14	**18**	benzyl	(*S*)-isomer
15	**19**	benzyl	(*R*)-isomer

Table S3. Tested compounds (R^2 variations)

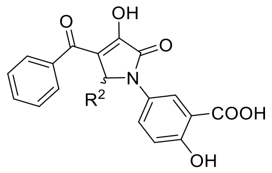

Index	Cpd.	R^2	Isomerism
1	**82**	phenyl	racemic mixture
2	**10**	4-aminophenyl	racemic mixture
3	**83**	3-acetamido-phenyl	racemic mixture
4	**84**	4-acetamido-phenyl	racemic mixture
5	**7**	1*H*-indazol-6-yl	racemic mixture
6	**85**	1-methyl-1*H*-indazol-6-yl	racemic mixture
7	**86**	1*H*-indazol-5-yl	racemic mixture
8	**87**	1-oxoisoindolin-5-yl	racemic mixture
9	**88**	2-methyl-1-oxoisoindolin-5-yl	racemic mixture
10	**6**	1*H*-benzo[*d*][1,2,3]triazol-5-yl	racemic mixture
11	**8**	1-methyl-1*H*-benzo[*d*][1,2,3]triazol-5-yl	racemic mixture
12	**89**	benzo[*c*][1,2,5]oxadiazol-5-yl	racemic mixture
13	**90**	1-oxo-1,3-dihydroisobenzofuran-5-yl	racemic mixture
14	**9**	isoquinolin-6-yl	racemic mixture
15	**91**	3,4-dimethoxy-phenyl	racemic mixture

Table S4. Tested compounds (R’, R^3 variations)

Index	Cpd. ID		R’	R^3
1	**92**		H	phenyl
2	**93**		ethyl	phenyl
3	**94**	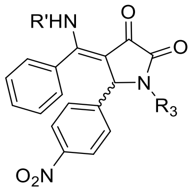	H	3-carboxy-4-hydroxyphenyl
4	**13**		4-methoxy-benzyl	3-carboxy-4-hydroxyphenyl
5	**14**	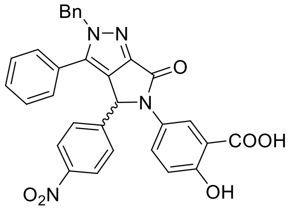		
6	**15**	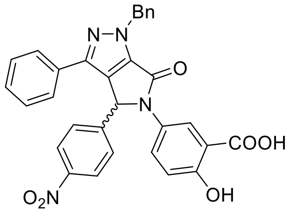		

Table S5. Quantification of the EMSA results (IC_{50}) for the pyrrolinone inhibitors that disrupted the LIN28–*let-7* complex formation shown in Figure 3.

Cpd.	IC_{50} (μM)
**3**	33
**10**	<15[a]
**13**	26
**9**	<15[a]
**4**	19
**6**	20
**12**	<15[a]
**11**	27
**5**	32
**7**	34
**8**	>75[a]

[a]Estimation based on the EMSA quantification method: band intensities were quantified by calculation of the ratio of free RNA fluorescence to the fluorescence of the protein–RNA complex, and normalization to free RNA and LIN28–*let-7* complex controls.

Supplementary Figures

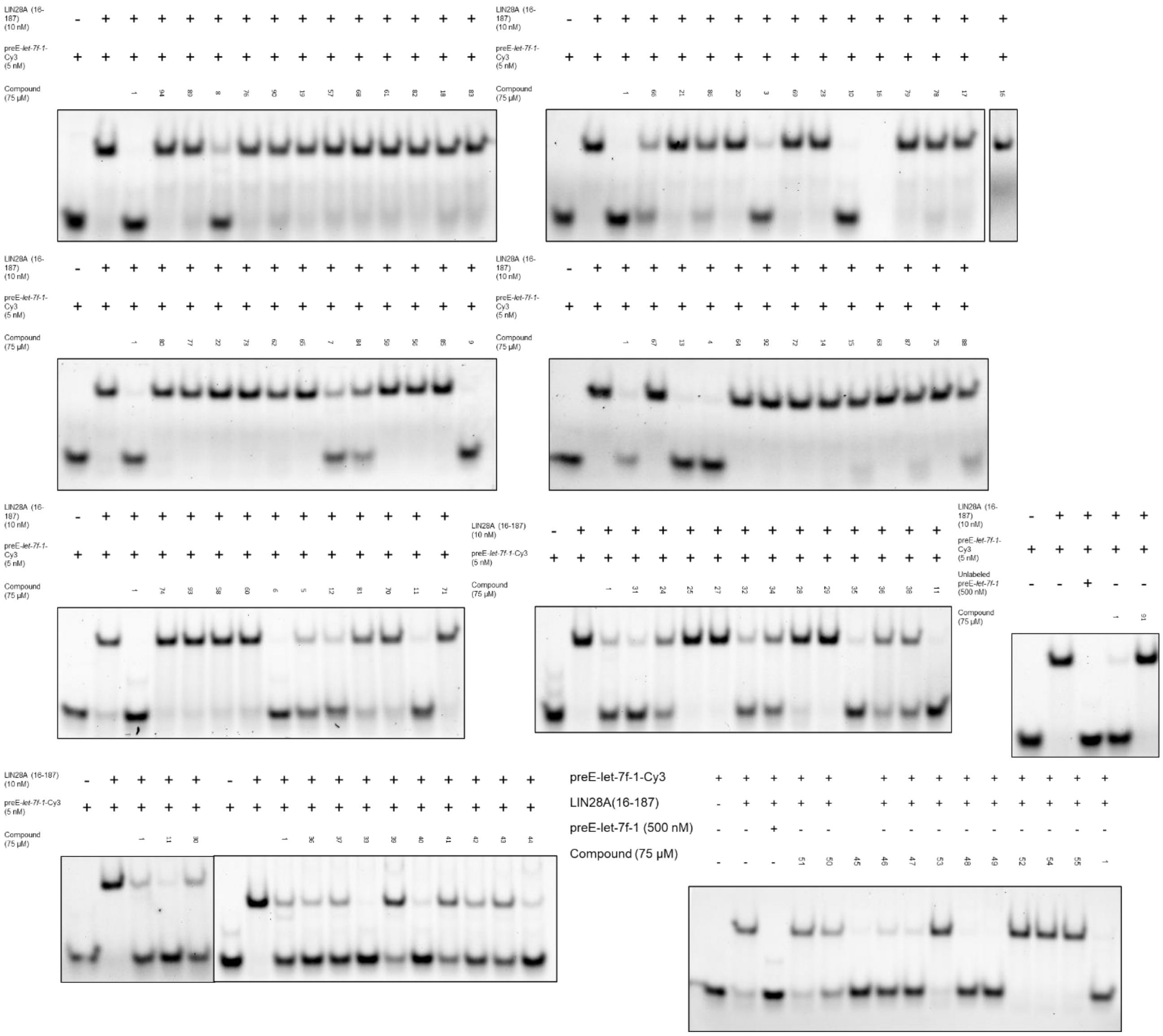

Figure S1. Single-dose screening EMSAs of trisubstituted pyrrolinones from this study.

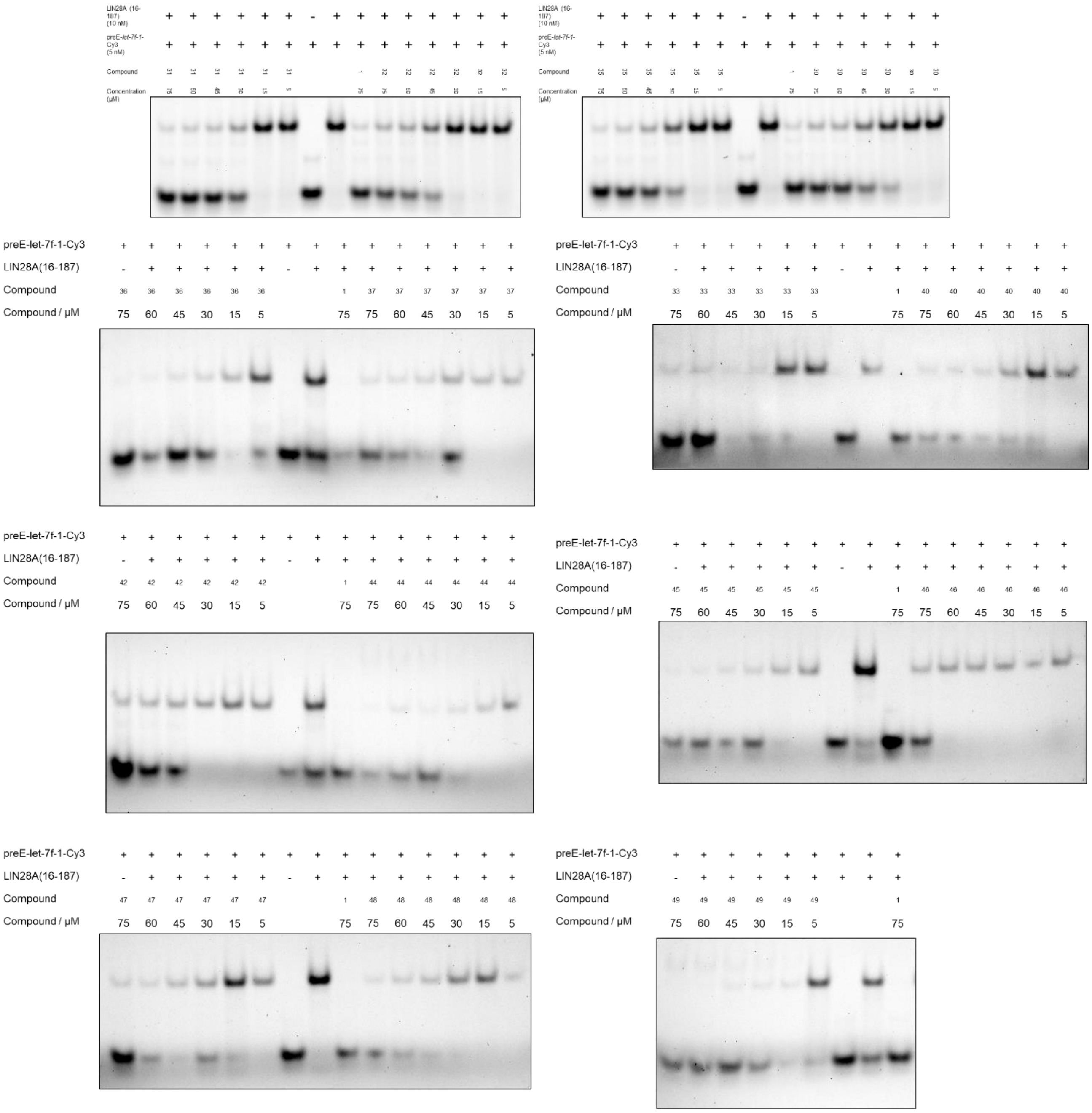

Figure S2. Dose-dependent EMSAs of compounds with activity above 60 % in screening EMSA.

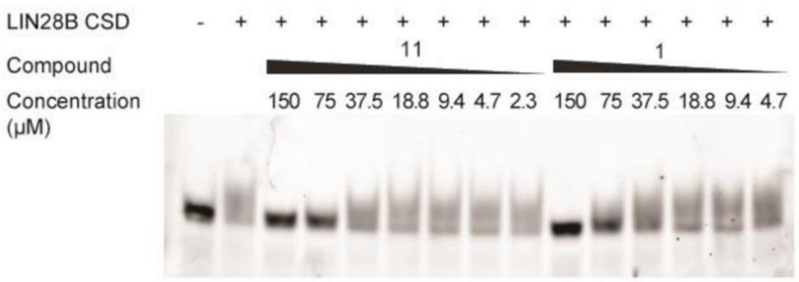

Figure S3. Dose-dependent EMSA of compounds **11** and **1** using LIN28B cold shock domain (CSD).

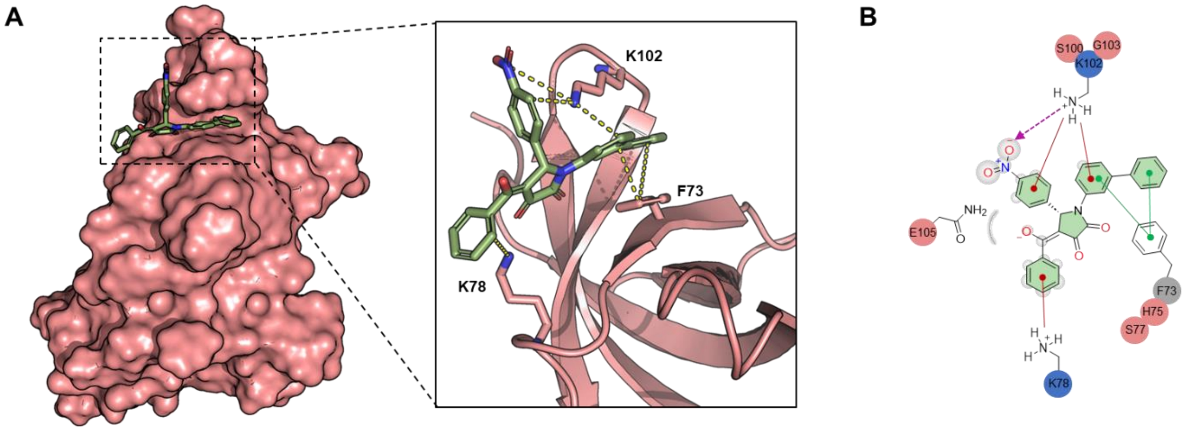

Figure S4. Molecular docking analysis of **11** with LIN28A (PDB code: 5UDZ). (A) An optimal docking configuration of **11** in complex with the RNA-binding site of LIN28A. Surface structure of LIN28A (salmon) and **11** in green carbon backbone (left) and the enlarged show of the ribbon structure of LIN28A with **11** (right). Selected key interacting residues are depicted as sticks. (B) 2D illustration of the binding interaction between **11** and LIN28A. The binding mode shows a salt bridge between K102 and the nitro of **11** and an additional cation–π interactions with the biphenyl moiety and the nitrophenyl ring of **11**, as well as a π–π interactions involving F73. Another cation–π interaction is formed with K78 and the benzoyl residue of **11**, underlining the importance of the aromatic moiety in contributing to LIN28 binding.

Supplementary Methods

Nano differential scanning fluorimetry (nanoDSF)

Compounds (final concentration 75 µM) were incubated with LIN28A (16-126) CSD protein for 45 minutes at room temperature in a buffer containing 30 mM NaH_2PO4, 50 mM NaCl and 1 mM $MgCl_2$ at pH 8.0. Thermal protein stability was determined using NanoTemper Prometheus NT.48 with a temperature ramp from 20 °C to 90 °C increasing the temperature by 1 °C per minute. Melting temperatures were determined using the software of the device.

Biolayer Interferometry (BLI)

Recombinant LIN28A CSD was biotinylated with EZ-Link Sulfo NHS-LC-LC-Biotin (Thermo Fisher Scientific) following the manufacturer's guidelines with a labeling ratio of one biotin per protein. Free biotin reagent was removed during dialysis against buffer (20 mM HEPES pH 7.5, 150 mM NaCl, 1 mM $MgCl_2$). After loading optimization, the protein was immobilized on SA biosensors (Sartorius). The association and dissociation of compounds were recorded in a buffer containing 20 mM HEPES pH 7.5, 150 mM NaCl, 1 mM $MgCl_2$, 0.05 % Tween 20, 1 % DMSO and 0.3 mg/mL BSA. Data was measured and analyzed using an Octet Red384 instrument (Sartorius). Double referencing against samples without compound and sensors loaded with 10 µg/mL biocytin instead of protein was performed.

qPCR

JAR cells (obtained from DSMZ, German Collection of Microorganisms and Cell Cultures, Braunschweig, Germany, DSMZ no. ACC462) were plated in six-well plates and three independent replicates per compounds were treated at indicated concentrations (0.5 % DMSO) for 24 h followed by extraction of total RNA using the RNeasy Mini Kit (Qiagen). Reverse transcription of 20 ng total RNA was performed following the protocol of the TaqMan microRNA Reverse Transcription Kit (Applied Biosystems). Then, qPCR was done with TaqMan microRNA assays purchased from Applied Biosystems (Assay IDs: 001973, 002221, 002283) detecting for *let-7d* and *let-7i* using U6 snRNA as a control. The assay was done with two technical replicates from three independent biological replicates using TaqMan Universal Master Mix II, with UNG (Applied Biosystems) and a CFX Connect Real-Time PCR System

(BioRad). Results were normalized to U6 snRNA expression and DMSO control using the $2^{-\Delta\Delta CT}$ method.

Docking Analysis

For computational docking analysis of pyrrolinones **11S/R** to the RNA binding site of LIN28A (PDB code: 5UDZ)[1] Schrödinger® Maestro 12.3 was used. The three-dimensional structures of compounds were prepared after performing energy minimization by MM2 with PerkinElmer Chem3D® 22.2. Chemical states were generated with the ligand preparation module and LIN28A conformation was prepared with the protein preparation module. Crucial interactions of *let-7* with LIN28A were identified based on the resolved structure of the RNP complex. The binding site for docking was defined by crucial residues of LIN28A involved in the binding of *let-7* and generated by the grid generation module. The glide dock module was used and the results were evaluated according to interactions between small molecules and LIN28A, small molecule orientations, docking scores and solvent exposure patterns. The interactions of small molecules (pyrrolinones) and LIN28A were visualized using PyMOL 2.5.2.

General Chemistry Information

All commercially available reagents and solvents were used without further purification unless noted otherwise. Dry solvents were purchased from Fischer Scientific, VWR and/or Acros and used without further treatment. Oxygen and/or moisture sensitive solutions were transferred using syringes and cannulas under inert gas.

Thin layer chromatography (TLC) was performed on silica coated aluminum plates (Merck 60 F254) and visualized under UV irradiation (254 nm) or through potassium permanganate stain (1.5 g $KMnO_4$, 10 g K_2CO_3, 1.25 mL of 10% aqueous NaOH solution and 200 mL of water).

Analytical uHPLC-MS and LC-MS was performed on an Agilent 1260 II Infinity system equipped with a mass detector (UHPLC column: Zorbax Eclipse C18 Rapid Resolution 2.1x50 mm 1.8µm; LC-MS column: InfinityLab Poroshell 120 EC-C18, 2.1x150, 2.7 µm). Appropriate gradient systems were applied by mixing Acetonitrile (+ 0.1%) and Water (+ 0.1% TFA).

Purification of crude products was achieved through flash column chromatography (FC, silica gel 60 Å, 0.035-0.070 mm) or automated medium pressure liquid chromatography (MPLC, Buchi Pure C-810, Buchi Pure C-835) using the indicated solvents. Challenging separations were carried out on an Buchi Pure C-835 system (columns: Nuleodur C18 gravity VP 125/10 5 µm). Appropriate gradient systems were applied by mixing Acetonitrile (+ 0.1% TFA) and Water (+ 0.1% TFA). Chiral separation of enantiomers was performed by chiral column chromatography on a Chiralpak IC (250 mm × 20 mm, 5 μm) column.

NMR spectra were recorded on Bruker AV 400 Avance III HD (NanoBay), Agilent Technologies DD2, Bruker AV 500 Avance III HD (Prodigy), Bruker AV 600 Avance III HD (CryoProbe) or Bruker AV 700 Avance III HD (CryoProbe) spectrometers. Data is reported in parts per million (ppm) with reference to the used deuterated solvent ($CDCl_3$: 7.26 ppm, 77.16 ppm; DMSO-d_6: 2.50 ppm, 39.52 ppm; Acetone-d_6: 2.05 ppm, 29.84 ppm, 206.26 ppm).[1] Chemical shift value is reported in ppm, multiplicity (s = singlet, d = doublet, t = triplet, dd = double doublet, and m = multiplet), integration value, and coupling constant value in Hz. Signals were assigned to their corresponding Hydrogens or Carbons based on 2D NMR correlations ($^1H/^1H$ COSY, $^1H/^1H$ NOESY, $^1H/^{13}C$ HSQC, $^1H/^{13}C$ HMBC).

High-resolution mass spectrometry (HRMS) was performed on an LTQ Orbitrap mass spectrometer coupled to an Accela HPLC-System (HPLC column: Hypersyl GOLD, 50 mm x 1 mm, particle size 1.9 µm, ionization method: electron spray ionization (ESI)).

General Procedure for the Doebner Condensation

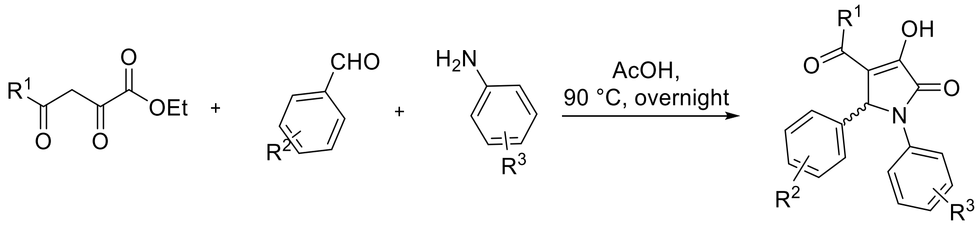

Scheme S1.

The benzaldehyde derivative (1.00 equiv) followed by the aniline derivative (1.00 equiv) were added to a suspension (0.06 M) of the dioxobutanoate component (1.00 equiv) in acetic acid.[1] The reaction mixture was stirred, overnight at 90 °C. After being cooled to room temperature the mixture was diluted with Et_2O and filtered. The residue was washed with Et_2O to give the desired pyrrolinone derivative after drying. In the case that precipitation did not yield pure product, the precipitate was further purified with an appropriate gradient on a prepartive HPLC system.

2-hydroxy-5-(3-hydroxy-4-(4-methylbenzoyl)-5-(4-nitrophenyl)-2-oxo-2,5-dihydro-1*H*-pyrrol-1-yl)benzoic acid (3)

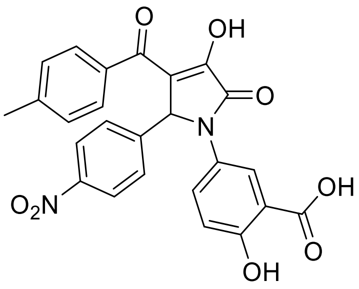

Prepared from methyl 2,4-dioxo-4-(*p*-tolyl)butanoate (250 mg, 1.14 mmol), 4-nitrobenzaldehyde (175 mg, 1.14 mmol) and 5-amino-2-hydroxybenzoic acid (183 mg, 1.14 mmol). After solvent removal, the crude product was purified by preparative HPLC (5–95% ACN with 0.1 M FA, flow rate 30 mL/min). Collected fractions were freeze-dried, to give the title compound (14 mg, 3%) as a off-white solid. **^{1}H NMR** (500 MHz, DMSO, 25°C) δ 2.34 (s, 3H), 6.45 (s, 1H), 6.90 (d, *J* = 8.9 Hz, 1H), 7.26 (d, *J* = 8.0 Hz, 2H), 7.6–7.65 (m, 2H), 7.69 (dd, *J* = 9.0, 2.4 Hz, 3H), 8.01–8.08 (m, 3H), 11.19 (s, 1H), 12.06 (s, 1H). **^{13}C NMR** (126 MHz, DMSO, 25°C) δ 21.18, 60.85, 113.04, 117.58, 119.40, 123.54, 124.89, 128.77, 129.27, 130.54, 135.23, 143.23, 144.41, 147.20, 150.44, 158.76, 164.54, 171.21, 188.67. **HRMS**-ESI *m/z* $[M + H]^+$ calcd for $C_{25}H_{19}N_2O_8$: 475.1141, found: 475.1125.

2-hydroxy-5-(3-hydroxy-5-(4-nitrophenyl)-2-oxo-4-picolinoyl-2,5-dihydro-1*H*-pyrrol-1-yl)benzoic acid (4):

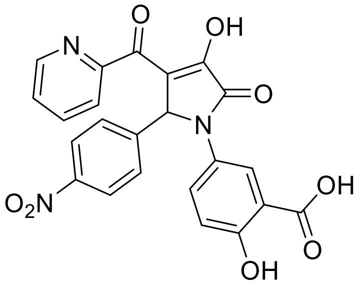

Prepared from ethyl 2,4-dioxo-4-(pyridin-2-yl)butanoate (150 mg, 0.64 mmol), 4-nitrobenzaldehyde (97 mg, 0.64 mmol) and 5-amino-2-hydroxybenzoic acid (99 mg, 0.64 mmol). After solvent removal, the crude product was purified by preparative HPLC (5-95% ACN in 0.1 M FA, flow rate 60 mL/min). The collected fractions were freeze-dried, to give the title compound (29 mg, 10%) as a pale-yellow solid. **^{1}H NMR** (500 MHz, DMSO, 25°C) δ 6.37 (s, 1H), 6.88 (d, *J* = 8.9 Hz, 1H), 7.63–7.68 (m, 2H), 7.70 (dd, *J* = 9.0, 2.8 Hz, 1H), 8.02 (ddd, *J* = 7.7, 5.1, 1.3 Hz, 1H), 8.04–8.08 (m, 3H), 8.15 (dt, *J* = 8.0, 1.1 Hz, 1H), 8.36 (td, *J* = 7.8, 1.6 Hz, 1H), 9.00 (ddd, *J* = 5.1, 1.7, 0.8 Hz, 1H). **^{13}C NMR** (126 MHz, DMSO, 25°C) δ 60.17, 113.16, 117.49, 117.77, 123.30, 124.55, 124.86, 127.55, 129.37, 129.46, 130.26, 142.13, 145.62, 145.80, 146.97, 149.85, 157.61, 158.82, 163.88, 171.20, 180.86. **HRMS**-ESI *m/z* $[M + H]^+$ calcd for $C_{23}H_{16}N_3O_8$: 462.0937, found: 462.0941.

2-hydroxy-5-(3-hydroxy-5-(4-nitrophenyl)-2-oxo-4-(thiazol-2-yl)-2,5-dihydro-1*H*-pyrrol-1-yl)benzoic acid (5):

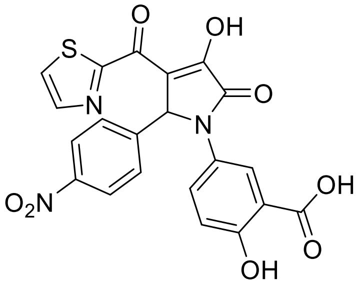

Prepared from ethyl 2-oxo-3-(thiazol-2-yl)propanoate (227 mg, 1.14 mmol), 4-nitrobenzaldehyde (172 mg, 1.14 mmol) and 5-amino-2-hydroxybenzoic acid (175 mg, 1.14 mmol). After solvent removal, the crude product was purified by preparative HPLC (5–95% ACN with in 0.2% NH_3, pH 10, flow rate 30 mL/min). Collected fractions were freeze-dried, to give the title compound (131 mg, 26%) as a off-white solid. **^{1}H NMR** (500 MHz, DMSO, 25°C) δ 6.36 (s, 1H), 6.55 (d, *J* = 8.7 Hz, 1H), 6.69 (s, 1H), 7.30 (dd, *J* = 8.7, 2.8 Hz, 3H),

7.55–7.59 (m, 2H), 7.60 (d, *J* = 3.3 Hz, 1H), 7.66 (d, *J* = 3.2 Hz, 1H), 7.77 (d, *J* = 2.8 Hz, 1H), 8.02–8.09 (m, 2H). One solvent peak observed at 1.76 ppm. **^{13}C NMR** (126 MHz, DMSO, 25°C) δ 61.25, 115.96, 119.32, 119.92, 123.32, 125.24, 125.57, 127.12, 129.36, 142.09, 146.15, 146.93, 158.23, 160.78, 164.81, 170.87, 171.51. One solvent peak observed at 22.54 ppm. **HRMS**-ESI *m/z* $[M + H]^+$ calcd for $C_{20}H_{13}N_3O_7S$: 440.0552, found: 440.0554.

5-(2-(1H-benzo[*d*][1,2,3]triazol-5-yl)-3-benzoyl-4-hydroxy-5-oxo-2,5-dihydro-1*H*-pyrrol-1-yl)-2-hydroxybenzoic acid (6)

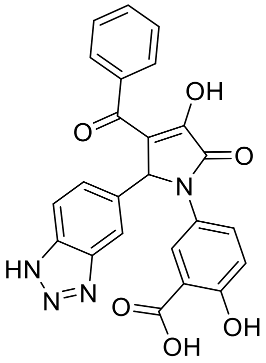

13

Prepared from ethyl 2,4-dioxo-4-phenylbutanoate (250 mg, 1.14 mmol), 1*H*-benzo[*d*][1,2,3]triazole-5-carbaldehyde (177 mg, 1.14 mmol) and 5-amino-2-hydroxybenzoic acid (174 mg, 1.14 mmol) After solvent removal, the reaction mixture was triturated with diethyl ether. The solid residue was washed with diethyl ether and subsequently purified by preparative HPLC (5-95% ACN in 0.1 M FA, flow rate 60 mL/min). The collected fractions were freeze-dried, to give the title compound (173 mg, 33%) as a yellow solid. **^{1}H NMR** (500 MHz, DMSO, 25°C) δ 2.54 (s, 1H), 6.46 (s, 1H), 6.86 (d, *J* = 8.9 Hz, 1H), 7.38 (s, 1H), 7.44 (t, *J* = 7.7 Hz, 2H), 7.51–7.58 (m, 1H), 7.69–7.75 (m, 3H), 8.03 (d, *J* = 2.7 Hz, 1H), 11.98 (s, 1H). **^{13}C NMR** (126 MHz, DMSO, 25°C) δ 59.93, 111.34, 115.70, 117.99, 123.26, 125.94, 126.52, 127.10, 128.94, 131.04, 136.20, 148.88, 157.01, 162.76, 169.54, 187.59. Because of tautomerism the three signals from the benzotriazole are too weak and cannot be detected by NMR. **HRMS**-ESI *m/z* $[M + H]^+$ calcd for $C_{24}H_{17}N_4O_6$: 457.1148, found: 457.1141.

5-(3-benzoyl-4-hydroxy-2-(1*H*-indazol-6-yl)-5-oxo-2,5-dihydro-1*H*-pyrrol-1-yl)-2-hydroxybenzoic acid (7):

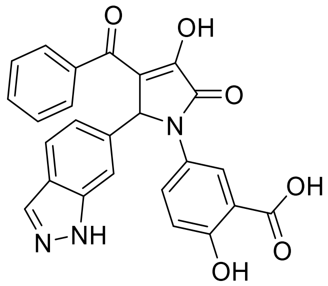

Prepared from ethyl 2,4-dioxo-4-phenylbutanoate (250 mg, 1.14 mmol), 1H-indazole-6-carbaldehyde (170 mg, 1.14 mmol) and 5-amino-2-hydroxybenzoic acid (174 mg, 1.14 mmol) After solvent removal, the reaction mixture was triturated with diethyl ether. The solid residue was washed with diethyl ether and subsequently purified by preparative HPLC (5-90% ACN in 0.2% NH_3, flow rate 60 mL/min). The collected fractions were freeze-dried, to give the title compound (128 mg, 24%) as a off-white solid. **^{1}H NMR** (500 MHz, DMSO, 25°C) δ 6.37 (s, 1H), 6.81 (d, *J* = 8.9 Hz, 1H), 6.99 (dd, *J* = 8.6, 1.3 Hz, 1H), 7.44 (t, *J* = 7.7 Hz, 2H), 7.51–7.59 (m, 3H), 7.65 (dd, *J* = 8.9, 2.8 Hz, 1H), 7.68–7.73 (m, 2H), 7.90 (d, *J* = 1.0 Hz, 1H), 7.99 (d, *J* = 2.8 Hz, 1H), 12.99 (s, 1H). **^{13}C NMR** (126 MHz, DMSO, 25°C) δ 62.01, 110.77, 113.49, 117.24, 118.52, 119.93, 120.82, 122.53, 124.91, 127.59, 128.22, 128.78, 130.36, 132.72, 133.27, 134.16, 137.89, 139.60, 150.26, 158.88, 164.48, 171.20, 189.29. **HRMS**-ESI *m/z* $[M + H]^+$ calcd for $C_{25}H_{17}N_3O_6$: 455.1117, found: 455.1198.

5-(3-benzoyl-4-hydroxy-2-(1-methyl-1*H*-benzo[*d*][1,2,3]triazol-5-yl)-5-oxo-2,5-dihydro-1*H*-pyrrol-1-yl)-2-hydroxybenzoic acid (8):

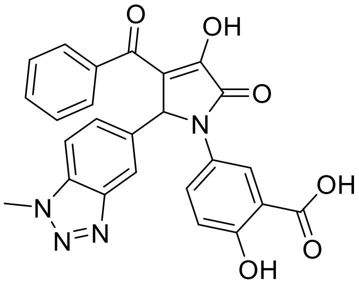

Prepared from ethyl 2,4-dioxo-4-phenylbutanoate (250 mg, 1.14 mmol), 1-methyl-1H-benzo[d][1,2,3]triazole-5-carbaldehyde (193 mg, 1.14 mmol) and 5-amino-2-hydroxybenzoic acid (174 mg, 1.14 mmol) After solvent removal, the reaction mixture was triturated with diethyl ether. The solid residue was washed with diethyl ether and dried under reduced pressure, to give the title compound (167 mg, 31%) as a yellow solid. **^{1}H NMR** (500 MHz, DMSO, 25°C) δ 4.16 (s, 3H), 6.45 (s, 1H), 6.83 (d, *J* = 8.9 Hz, 1H), 7.43 (t, *J* = 7.7 Hz, 2H), 7.48–7.58 (m, 2H), 7.64 (dd, *J* = 8.7, 0.8 Hz, 1H), 7.70 (dd, *J* = 8.9, 2.7 Hz, 1H), 7.72–7.77 (m,

2H), 8.02 (d, *J* = 2.8 Hz, 1H), 8.10 (t, *J* = 1.1 Hz, 1H). ^{13}**C NMR** (126 MHz, DMSO, 25°C) δ 40.43, 111.09, 113.71, 117.22, 119.48, 119.58, 125.02, 127.38, 128.18, 128.82, 130.27, 132.31, 132.68, 133.10, 137.93, 144.89, 150.80, 158.88, 164.45, 171.19, 189.23. **HRMS**-ESI *m/z* [M + H]$^+$ calcd for $C_{25}H_{18}N_4O_6$: 471.1304, found: 471.1299.

5-(3-benzoyl-4-hydroxy-2-(isoquinolin-6-yl)-5-oxo-2,5-dihydro-1*H*-pyrrol-1-yl)-2-hydroxybenzoic acid (9):

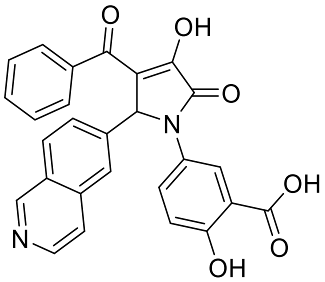

Prepared from ethyl 2,4-dioxo-4-phenylbutanoate (268 mg, 1.22 mmol), isoquinoline-6-carbaldehyde (201 mg, 1.22 mmol) and 5-amino-2-hydroxybenzoic acid (186 mg, 1.22 mmol). After solvent removal, the reaction mixture was triturated with diethyl ether. The solid residue was washed with diethyl ether, then taken up in 1,4-dioxane and stirred for 15 min. The resulting suspension was filtered, the filtrate was evaporated under reduced pressure and purified by preparative HPLC (5-95% ACN in 0.1 M FA, flow rate 60 mL/min). The collected fractions were freeze-dried, to give the title compound (20 mg, 4%) as a yellow solid. ^{1}**H NMR** (500 MHz, DMSO, 25°C) δ 6.46 (s, 1H), 6.85 (d, *J* = 8.9 Hz, 1H), 7.43 (t, *J* = 7.7 Hz, 2H), 7.5–7.57 (m, 1H), 7.64–7.74 (m, 4H), 7.80 (d, *J* = 5.8 Hz, 1H), 7.98 (d, *J* = 8.6 Hz, 1H), 8.04 (dd, *J* = 8.5, 2.2 Hz, 2H), 8.43 (d, *J* = 5.8 Hz, 1H), 9.18 (s, 1H). ^{13}**C NMR** (126 MHz, DMSO, 25°C) δ 113.37, 117.37, 119.27, 120.47, 125.02, 126.22, 126.51, 127.51, 127.65, 128.16, 128.24, 128.73, 130.45, 132.65, 135.06, 137.92, 139.42, 142.57, 151.10, 151.69, 158.86, 164.64, 171.14, 189.13. **HRMS**-ESI *m/z* [M + H]$^+$ calcd for $C_{27}H_{18}N_2O_6$: 467.1243, found: 467.1241.

5-(2-(4-aminophenyl)-3-benzoyl-4-hydroxy-5-oxo-2,5-dihydro-1*H*-pyrrol-1-yl)-2-hydroxybenzoic acid (10)

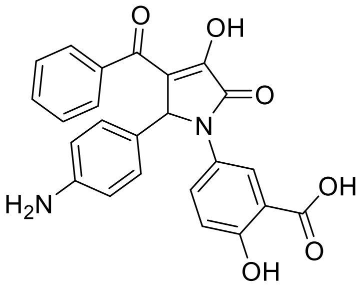

The racemic mixture was synthesized according to reported procedures and the compound characterization correlates with previously reported data.[1]

1-([1,1'-biphenyl]-3-yl)-4-benzoyl-3-hydroxy-5-(4-nitrophenyl)-1,5-dihydro-2*H*-pyrrol-2-one (11):

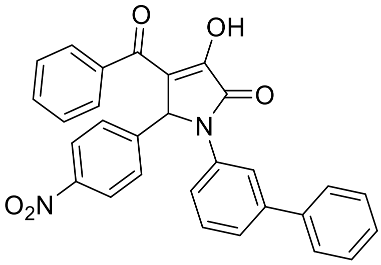

4-Nitrobenzaldehyde (44.65 mg, 0.30 mmol, 1.00 equiv.) followed by the 3-Aminobiphenyl (50.00 mg, 0.30 mmol, 1.00 equiv.) were added to a suspension (6.0 mL, 0.06 M) of Ethyl 2,4-dioxo-4-phenylbutanoate (65.07 mg, 0.30 mmol, 1.00 equiv.) in acetic acid following the general procedure to give the desired product as a yellow solid (38.6 mg, 24%). **^{1}H NMR** (700 MHz, DMSO-d_6) δ 12.21 (s, 1H), 8.06 (d, *J* = 8.9 Hz, 2H), 7.96 (t, *J* = 1.9 Hz, 1H), 7.80 (d, *J* = 8.9 Hz, 2H), 7.74 (dd, *J* = 8.3, 1.3 Hz, 2H), 7.66 (dt, *J* = 7.4, 1.9 Hz, 1H), 7.63 (dd, *J* = 8.3, 1.3 Hz, 2H), 7.45 (s, 5H), 7.42 (d, *J* = 29.0 Hz, 3H), 6.68 (s, 1H). **^{13}C NMR** (176 MHz, DMSO-d_6) δ 189.53, 165.21, 147.64, 145.17, 141.28, 139.77, 138.34, 137.12, 133.22, 129.94, 129.74, 129.50, 129.46, 129.22, 128.67, 128.29, 128.01, 127.22, 127.11, 127.04, 126.96, 124.44, 123.96, 122.17, 121.21, 119.76, 60.83.

HRMS-ESI (m/z): $[M+H]^+$ calculated for $C_{29}H_{21}N_2O_5$ $[M+H]^+$ 477.1445; found, 477.1447.

11*S* and 11*R*

The synthesis and the chiral separation of the racemic mixture was performed according to the reported procedure.[1]

4-benzoyl-3-hydroxy-5-(4-nitrophenyl)-1-(3-phenylpropyl)-1,5-dihydro-2*H*-pyrrol-2-one (12):

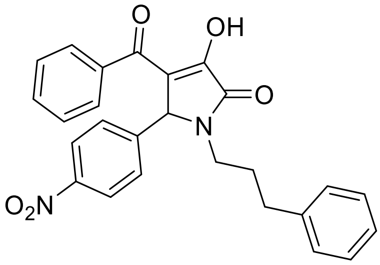

Prepared from ethyl 2,4-dioxo-4-phenylbutanoate (250 mg, 1.14 mmol), 4-nitrobenzaldehyde (172 mg, 1.14 mmol) and 3-phenylpropan-1-amine (0.162 mL, 1.14 mmol). After solvent removal, the reaction mixture was triturated with diethyl ether. The solid residue was washed with diethyl ether and subsequently purified by preparative HPLC (2-94% ACN in 0.2% NH3, flow rate 60 mL/min). The collected fractions were freeze-dried, to give the title compound (191 mg, 38%) as a pale yellow solid. **^{1}H NMR** (500 MHz, DMSO-d_6) δ 1.73 (dddd, *J* = 28.4, 14.4, 7.6, 4.1 Hz, 2H), 2.48 (d, *J* = 6.5 Hz, 1H), 2.51–2.56 (m, 1H), 2.72 (ddd, *J* = 13.7, 8.0, 5.4 Hz, 1H), 3.60 (dt, *J* = 13.9, 7.7 Hz, 1H), 5.66 (s, 1H), 7.11–7.18 (m, 3H), 7.19–7.27 (m, 2H), 7.43 (t, *J* = 7.7 Hz, 2H), 7.5–7.57 (m, 1H), 7.66 (ddd, *J* = 11.0, 7.4, 1.6 Hz, 4H), 8.13–8.18 (m, 2H). **^{13}C NMR** (126 MHz, DMSO, 25°C) δ 28.91, 32.21, 59.96, 118.54, 123.73, 125.80, 128.07, 128.21, 128.26, 129.26, 132.43, 138.14, 141.09, 144.70, 147.35, 152.31, 165.45, 188.77. **HRMS**-ESI (m/z) [M+H]+ calculated for $C_{26}H_{23}N_2O_5$ [M+H]+: 443.1607, found: 443.1618.

(Z)-2-hydroxy-5-(3-(((4-methoxybenzyl)amino)(phenyl)methylene)-2-(4-nitrophenyl)-4,5-dioxopyrrolidin-1-yl)benzoic acid (13)

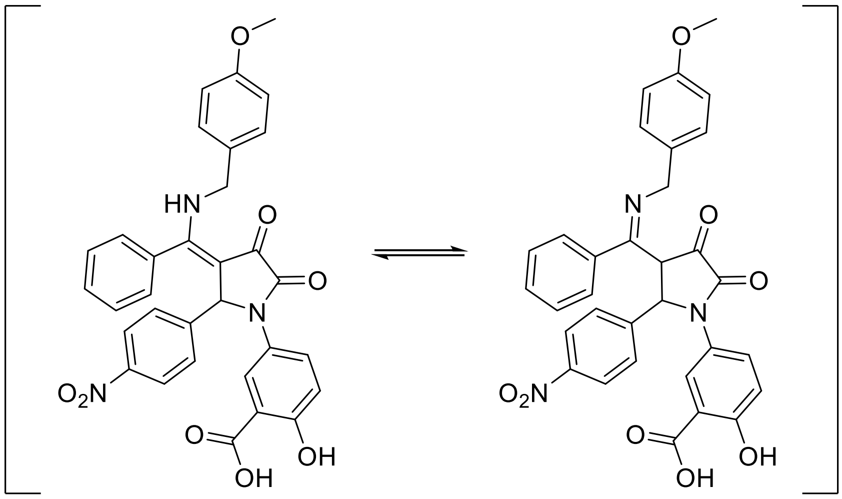

The racemic mixture was synthesized according to the reported procedure and the characterization correlates with previously reported data.[1]

1-([1,1'-biphenyl]-3-yl)-4-(cyclohexanecarbonyl)-3-hydroxy-5-(4-nitrophenyl)-1,5-dihydro-2*H*-pyrrol-2-one (24):

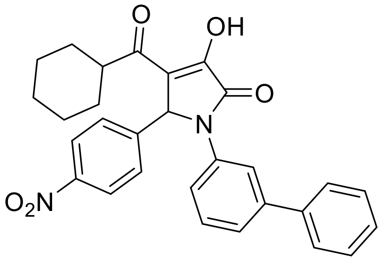

4-Nitrobenzaldehyde (44.65 mg, 0.30 mmol, 1.00 equiv.) followed by the 3-Aminobiphenyl (50.00 mg, 0.30 mmol, 1.00 equiv.) were added to a suspension (6 mL, 0.06 M) of Ethyl-4-cyclohexyl-2,4-dioxobutanoate (**95e**) (133.71 mg, 0.30 mmol, 1.00 equiv.) in acetic acid following the general procedure to give the desired product as an orange solid (119.0 mg, 83%). **HRMS**-ESI (m/z): $[M+H]^+$ calculated for $C_{29}H_{27}N_2O_5$ $[M+H]^+$ 483.1915; found, 483.1918.

1-([1,1'-biphenyl]-3-yl)-4-(cyclopropanecarbonyl)-3-hydroxy-5-(4-nitrophenyl)-1,5-dihydro-2*H*-pyrrol-2-one (25):

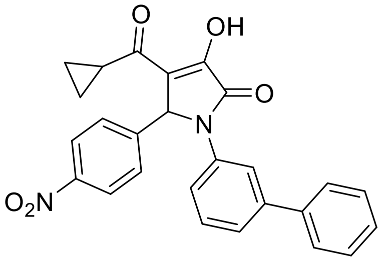

4-Nitrobenzaldehyde (44.65 mg, 0.30 mmol, 1.00 equiv.) followed by the 3-Aminobiphenyl (50.00 mg, 0.30 mmol, 1.00 equiv.) were added to a suspension (6 mL, 0.06 M) of Ethyl-4-cyclopropyl-2,4-dioxobutanoate (**95f**) (108.84 mg, 0.30 mmol, 1.00 equiv.) in acetic acid following the general procedure to give the desired product as an orange solid (13.0 mg, 9%). **HRMS**-ESI (m/z): $[M+H]^+$ calculated for $C_{26}H_{21}N_2O_5$ $[M+H]^+$ 441.1445; found, 441.1448.

1-([1,1'-biphenyl]-3-yl)-4-(cyclobutanecarbonyl)-3-hydroxy-5-(4-nitrophenyl)-1,5-dihydro-2*H*-pyrrol-2-one (26):

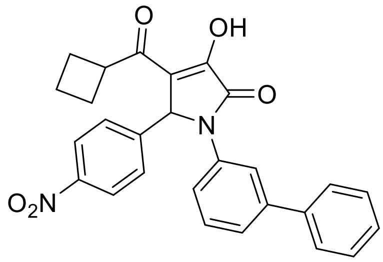

4-Nitrobenzaldehyde (44.65 mg, 0.30 mmol, 1.00 equiv.) followed by the 3-Aminobiphenyl (50.00 mg, 0.30 mmol, 1.00 equiv.) were added to a suspension (5 mL, 0.06 M) of Ethyl-4-cyclobutyl-2,4-dioxobutanoate (**95k**) (73.21 mg, 0.30 mmol, 1.00 equiv.) in acetic acid following the general procedure to give the desired product as a yellow solid (88.5 mg, 66%). **^{1}H NMR** (600 MHz, DMSO-d_6) δ 8.04 (d, *J* = 8.6 Hz, 2H), 7.89 (s, 1H), 7.60 (dd, *J* = 12.1, 8.2 Hz, 5H), 7.48 – 7.42 (m, 3H), 7.37 (s, 3H), 6.29 (s, 1H), 3.97 (s, 1H), 2.06 (s, 4H), 1.87 (s, 2H). **^{13}C NMR** (151 MHz, DMSO-d_6) δ 147.15, 141.14, 139.88, 129.81, 129.77, 129.76, 129.46, 129.42, 129.31, 128.20, 128.16, 127.18, 127.09, 123.61, 123.58, 121.74, 120.87, 67.49, 60.05, 25.60, 23.67, 17.75. **HRMS**-ESI (m/z): [M+H]$^+$ calculated for $C_{27}H_{23}N_2O_5$ [M+H]$^+$ 455.1601; found, 455.1605.

1-([1,1'-biphenyl]-3-yl)-4-(4-fluoro-2-hydroxybenzoyl)-3-hydroxy-5-(4-nitrophenyl)-1,5-dihydro-2*H*-pyrrol-2-one (27):

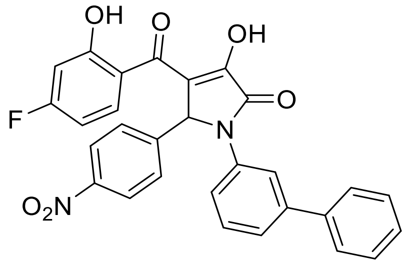

4-Nitrobenzaldehyde (44.65 mg, 0.30 mmol, 1.00 equiv.) followed by the 3-Aminobiphenyl (50.00 mg, 0.30 mmol, 1.00 equiv.) were added to a suspension (5 mL, 0.06 M) of Ethyl-4-(4-fluoro-2-hydroxyphenyl)-2,4-dioxobutanoate (**95g**) (125.19 mg, 0.30 mmol, 1.00 equiv) in acetic acid following the general procedure to give the desired product as a yellow solid (6.3 mg, 4%). **^{1}H NMR** (600 MHz, DMSO-d_6) δ 10.03 (s, 2H), 7.90 (t, *J* = 1.8 Hz, 2H), 7.60 (d, *J* = 8.2 Hz, 4H), 7.56 (d, *J* = 9.8 Hz, 2H), 7.48 (t, *J* = 7.7 Hz, 4H), 7.38 (td, *J* = 7.6, 3.1 Hz, 4H), 7.32 (d, *J* = 9.2 Hz, 2H). **LCMS-ESI** (m/z) calculated for $C_{29}H_{19}FN_2O_6$ [M+H]$^+$: 511.1; found: 511.0.

1-([1,1'-biphenyl]-3-yl)-3-hydroxy-4-(4-(methylsulfonyl)benzoyl)-5-(4-nitrophenyl)-1,5-dihydro-2*H*-pyrrol-2-one (28):

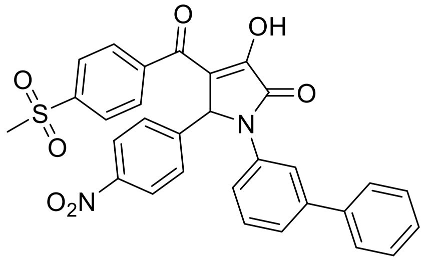

4-Nitrobenzaldehyde (44.65 mg, 0.30 mmol, 1.00 equiv.) followed by the 3-Aminobiphenyl (50.00 mg, 0.30 mmol, 1.00 equiv.) were added to a suspension (5 mL, 0.06 M) of Ethyl-4-(4-(methylsulfonyl)phenyl)-2,4-dioxobutanoate (**95j**) (146.90 mg, 0.30 mmol, 1.00 equiv.) in acetic acid following the general procedure to give the desired product as a yellow solid (46.6 mg, 28%). **HRMS**-ESI (m/z): $[M+H]^+$ calculated for $C_{30}H_{23}N_2O_7S$ $[M+H]^+$ 555.1221; found, 555.1227.

1-([1,1'-biphenyl]-3-yl)-4-(3,4-dimethoxybenzoyl)-3-hydroxy-5-(4-nitrophenyl)-1,5-dihydro-2*H*-pyrrol-2-one (29):

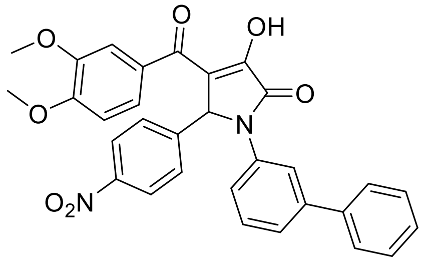

4-Nitrobenzaldehyde (44.65 mg, 0.30 mmol, 1.00 equiv.) followed by the 3-Aminobiphenyl (50.00 mg, 0.30 mmol, 1.00 equiv.) were added to a suspension (5 mL, 0.06 M) of Ethyl-4-(3,4-dimethoxyphenyl)-2,4-dioxobutanoate (**95b**) (103.52 mg, 0.30 mmol, 1.00 equiv.) in acetic acid following the general procedure to give the desired product as a yellow solid (86.0 mg, 49%). **HRMS**-ESI (m/z): $[M+H]^+$ calculated for $C_{31}H_{25}N_2O_7$ $[M+H]^+$ 537.1662; found, 537.1591.

1-([1,1'-biphenyl]-3-yl)-4-(4-bromobenzoyl)-3-hydroxy-5-(4-nitrophenyl)-1,5-dihydro-2*H*-pyrrol-2-one (30):

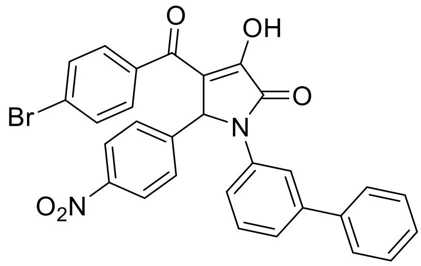

4-Nitrobenzaldehyde (44.65 mg, 0.30 mmol, 1.00 equiv.) followed by the 3-Aminobiphenyl (50.00 mg, 0.30 mmol, 1.00 equiv.) were added to a suspension (6.0 mL, 0.06 M) of Ethyl 4-(4-bromophenyl)-2,4-dioxobutanoate (88.38 mg, 0.30 mmol, 1.00 equiv.) in acetic acid following the general procedure to give the desired product as a yellow solid (40.4 mg, 22%). **^{1}H NMR** (600 MHz, DMSO-d_6) δ 8.94 (s, 1H), 8.04 (d, *J* = 8.9 Hz, 2H), 7.98 (s, 1H), 7.88 (d, *J* = 8.5 Hz, 2H), 7.83 (d, *J* = 8.5 Hz, 2H), 7.66 (d, *J* = 8.9 Hz, 2H), 7.65 – 7.59 (m, 2H), 7.47 (t, *J* = 7.7 Hz, 2H), 7.38 (d, *J* = 7.4 Hz, 2H), 7.35 (d, *J* = 7.3 Hz, 2H), 6.25 (s, 1H). **^{13}C NMR** (176 MHz, DMSO-d_6) δ 165.06, 147.66, 145.12, 141.29, 139.75, 137.44, 137.06, 132.70, 132.29, 131.76, 131.17, 130.38, 129.95, 129.92, 129.76, 129.50, 129.46, 128.30, 127.22, 127.11, 124.50, 123.95, 122.19, 121.22, 60.73. **HRMS**-ESI (m/z): $[M+H]^+$ calculated for $C_{29}H_{20}N_2O_5Br$ $[M+H]^+$ 555.0550; found, 555.0553.

1-([1,1'-biphenyl]-3-yl)-3-hydroxy-4-(4-methoxybenzoyl)-5-(4-nitrophenyl)-1,5-dihydro-2*H*-pyrrol-2-one (31):

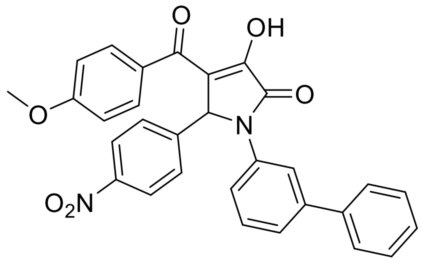

4-Nitrobenzaldehyde (44.65 mg, 0.30 mmol, 1.00 equiv.) followed by the 3-Aminobiphenyl (50.00 mg, 0.30 mmol, 1.00 equiv.) were added to a suspension (5 mL, 0.06 M) of Ethyl-4-(4-methoxyphenyl)-2,4-dioxobutanoate (**95d**) (123.23 mg, 0.30 mmol, 1.00 equiv.) in acetic acid following the general procedure to give the desired product as a yellow solid (50.7 mg, 30%). **^{1}H NMR** (700 MHz, DMSO-d_6) δ 12.04 (s, 1H), 8.03 (d, *J* = 8.4 Hz, 2H), 7.96 (s, 1H), 7.80 (d, *J* = 8.3 Hz, 2H), 7.74 (d, *J* = 8.4 Hz, 2H), 7.68 – 7.64 (m, 1H), 7.61 (d, *J* = 7.6 Hz, 2H), 7.46 (t, *J* = 7.6 Hz, 2H), 7.42 – 7.34 (m, 3H), 6.94 (d, *J* = 8.4 Hz, 2H), 6.55 (s, 1H), 3.81 (s, 3H). **^{13}C NMR** (176 MHz, DMSO-d_6) δ 186.87, 166.69, 162.93, 147.31, 146.81, 141.18, 139.90, 137.63, 131.67, 129.85, 129.55, 129.43, 128.21, 127.19, 124.02, 123.74, 121.80,

120.92, 114.28, 113.59, 60.86, 55.87. **HRMS**-ESI (m/z): $[M+H]^+$ calculated for $C_{30}H_{23}N_2O_6$ $[M+H]^+$ 507.1551; found, 507.1557.

1-([1,1'-biphenyl]-3-yl)-4-(4-fluorobenzoyl)-3-hydroxy-5-(4-nitrophenyl)-1,5-dihydro-2*H*-pyrrol-2-one (32):

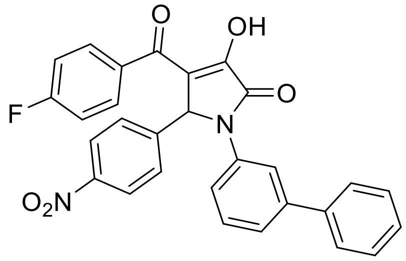

4-Nitrobenzaldehyde (44.65 mg, 0.30 mmol, 1.00 equiv.) followed by the 3-Aminobiphenyl (50.00 mg, 0.30 mmol, 1.00 equiv.) were added to a suspension (5 mL, 0.06 M) of Ethyl-4-(4-fluorophenyl)-2,4-dioxobutanoate (**95h**) (125.19 mg, 0.30 mmol, 1.00 equiv.) in acetic acid following the general procedure to give the desired product as a yellow solid (84.4 mg, 58%). **^{1}H NMR** (700 MHz, DMSO-d_6) δ 7.99 (s, 2H), 7.90 (s, 2H), 7.61 (dd, J = 23.3, 10.4 Hz, 6H), 7.46 (t, J = 7.6 Hz, 3H), 7.37 (t, J = 7.0 Hz, 3H), 7.10 (t, J = 8.3 Hz, 2H), 6.36 (s, 1H). **^{13}C NMR** (176 MHz, DMSO-d_6) δ 183.83, 170.33, 164.33, 162.94, 162.77, 150.17, 146.69, 141.09, 140.06, 138.27, 137.51, 131.62, 129.75, 129.42, 129.22, 128.16, 127.18, 123.67, 123.33, 121.25, 120.51, 115.45, 114.35, 114.22, 60.85. **HRMS**-ESI (m/z): $[M+H]^+$ calculated for $C_{29}H_{20}N_2O_5F$ $[M+H]^+$ 495.1351; found, 495.1354.

1-([1,1'-biphenyl]-3-yl)-4-(2-fluorobenzoyl)-3-hydroxy-5-(4-nitrophenyl)-1,5-dihydro-2*H*-pyrrol-2-one (33):

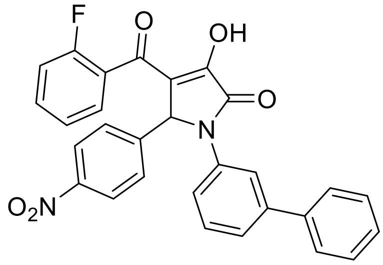

4-Nitrobenzaldehyde (44.65 mg, 0.30 mmol, 1.00 equiv.) followed by the 3-Aminobiphenyl (50.00 mg, 0.30 mmol, 1.00 equiv.) were added to a suspension (5 mL, 0.06 M) of Ethyl-4-(2-fluorophenyl)-2,4-dioxobutanoate (**95o**) (87.98 mg, 0.30 mmol, 1.00 equiv.) in acetic acid following the general procedure to give the desired product as a yellow solid (70.8 mg, 48%).

^{1}H NMR (700 MHz, DMSO-d_6) δ 8.13 – 8.08 (m, 2H), 7.95 (t, *J* = 2.0 Hz, 1H), 7.77 – 7.74 (m, 2H), 7.65 – 7.62 (m, 3H), 7.55 (dddd, *J* = 8.5, 7.1, 5.2, 1.8 Hz, 1H), 7.49 – 7.46 (m, 2H), 7.46 – 7.42 (m, 3H), 7.42 – 7.37 (m, 2H), 7.28 – 7.23 (m, 2H), 6.65 (s, 1H).
^{13}C NMR (176 MHz, DMSO-d_6) δ 164.93, 160.55, 159.13, 147.64, 145.25, 141.30, 139.72, 136.94, 133.64, 130.75, 130.07, 129.96, 129.75, 129.46, 128.64, 128.30, 127.22, 127.12, 124.87, 124.63, 123.93, 122.28, 121.35, 119.99, 116.35, 116.23, 60.36. **HRMS**-ESI (m/z): $[M+H]^+$ calculated for $C_{29}H_{20}N_2O_5F$ $[M+H]^+$ 495.1351; found, 495.1355.

1-([1,1'-biphenyl]-3-yl)-3-hydroxy-4-(3-hydroxy-4-nitrobenzoyl)-5-(4-nitrophenyl)-1,5-dihydro-2*H*-pyrrol-2-one (34):

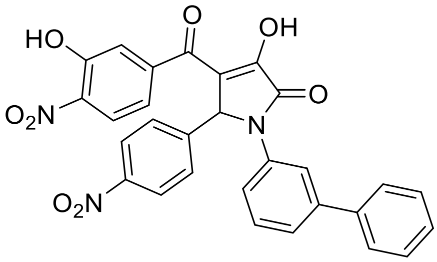

4-Nitrobenzaldehyde (44.65 mg, 0.30 mmol, 1.00 equiv.) followed by the 3-Aminobiphenyl (50.00 mg, 0.30 mmol, 1.00 equiv.) were added to a suspension (5 mL, 0.06 M) of Ethyl-4-(3-hydroxy-4-nitrophenyl)-2,4-dioxobutanoate (**95i**) (138.49 mg, 0.30 mmol, 1.00 equiv.) in acetic acid following the general procedure to give the desired product as a yellow solid (4.1 mg, 3%). **HRMS**-ESI (m/z): $[M+H]^+$ calculated for $C_{29}H_{20}N_3O_8$ $[M+H]^+$ 538.1244; found, 538.1249.

1-([1,1'-biphenyl]-3-yl)-4-(furan-2-carbonyl)-3-hydroxy-5-(4-nitrophenyl)-1,5-dihydro-2*H*-pyrrol-2-one (35):

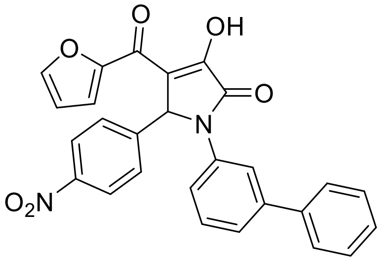

4-Nitrobenzaldehyde (44.65 mg, 0.30 mmol, 1.00 equiv.) followed by the 3-Aminobiphenyl (50.000 mg, 0.30 mmol, 1.00 equiv.) were added to a suspension (5 mL, 0.06 M) of Ethyl-4-(furan-2-yl)-2,4-dioxobutanoate (**95c**) (77.63 mg, 0.30 mmol, 1.00 equiv.) in acetic acid following the general procedure to give the desired product as a yellow solid (77.9 mg, 50%).

^{1}H NMR (700 MHz, DMSO-d_6) δ 8.07 – 8.03 (m, 2H), 7.97 (dd, *J* = 1.7, 0.7 Hz, 1H), 7.94 (t, *J* = 2.0 Hz, 1H), 7.74 – 7.70 (m, 2H), 7.67 – 7.66 (m, 1H), 7.66 – 7.63 (m, 2H), 7.62 (t, *J* = 1.1 Hz, 1H), 7.50 – 7.46 (m, 3H), 7.43 – 7.37 (m, 3H), 6.72 (dd, *J* = 3.6, 1.7 Hz, 1H), 6.67 (s, 1H). **^{13}C NMR** (176 MHz, DMSO-d_6) δ 174.69, 165.04, 151.99, 150.59, 148.52, 147.64, 145.05, 141.28, 139.74, 137.01, 129.93, 129.73, 129.44, 128.28, 127.22, 127.10, 127.04, 124.47, 123.94, 122.22, 121.25, 120.98, 119.42, 112.92, 60.66. **HRMS**-ESI (m/z): $[M+H]^+$ calculated for $C_{27}H_{19}N_2O_6$ $[M+H]^+$ 467.1243; found, 467.1241.

1-([1,1'-biphenyl]-3-yl)-3-hydroxy-4-(5-methylfuran-2-carbonyl)-5-(4-nitrophenyl)-1,5-dihydro-2*H*-pyrrol-2-one (36):

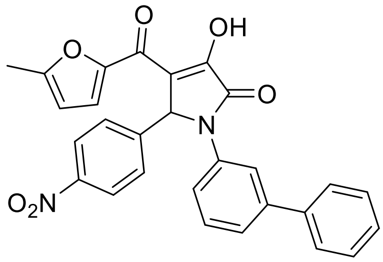

4-Nitrobenzaldehyde (44.65 mg, 0.30 mmol, 1.00 equiv.) followed by the 3-Aminobiphenyl (50.00 mg, 0.30 mmol, 1.00 equiv.) were added to a suspension (5 mL, 0.06 M) of Ethyl-4-(5-methylfuran-2-yl)-2,4-dioxobutanoate (**95m**) (82.81 mg, 0.30 mmol, 1.00 equiv.) in acetic acid following the general procedure to give the desired product as a yellow solid (46.7 mg, 33%). **^{1}H NMR** (700 MHz, DMSO-d_6) δ 8.05 (d, *J* = 8.9 Hz, 2H), 7.94 (t, *J* = 2.0 Hz, 1H), 7.70 (d, *J* = 8.9 Hz, 2H), 7.65 – 7.62 (m, 3H), 7.60 (d, *J* = 3.5 Hz, 1H), 7.47 (t, *J* = 7.7 Hz, 3H), 7.42 (t, *J* = 1.6 Hz, 1H), 7.40 – 7.38 (m, 2H), 6.66 (s, 1H), 6.37 (dd, *J* = 3.5, 1.1 Hz, 1H), 2.33 (s, 3H). **^{13}C NMR** (176 MHz, DMSO-d_6) δ 173.84, 165.13, 158.79, 150.94, 147.62, 145.08, 141.26, 139.75, 137.06, 129.92, 129.72, 129.49, 129.47, 129.44, 129.29, 128.28, 127.21, 127.10, 126.98, 124.40, 123.92, 123.08, 122.19, 121.22, 60.69, 14.10. **HRMS**-ESI (m/z): $[M+H]^+$ calculated for $C_{28}H_{21}N_2O_6$ $[M+H]^+$ 481.1400; found, 481.1398.

1-([1,1'-biphenyl]-3-yl)-3-hydroxy-4-(1-methyl-1*H*-pyrrole-2-carbonyl)-5-(4-nitrophenyl)-1,5-dihydro-2*H*-pyrrol-2-one (37):

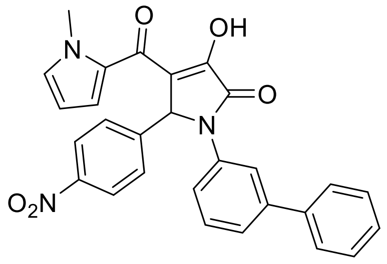

4-Nitrobenzaldehyde (44.65 mg, 0.30 mmol, 1.00 equiv.) followed by the 3-Aminobiphenyl (50.00 mg, 0.30 mmol, 1.00 equiv.) were added to a suspension (5 mL, 0.06 M) of Ethyl-4-(1-methyl-1H-pyrrol-2-yl)-2,4-dioxobutanoate (**95n**) (82.45 mg, 0.30 mmol, 1.00 equiv.) in acetic acid following the general procedure to give the desired product as a yellow solid (19.5 mg, 14%).

^{1}H NMR (700 MHz, DMSO-*d*$_6$) δ 11.54 (s, 1H), 8.00 (d, *J* = 8.4 Hz, 2H), 7.96 (s, 1H), 7.66 (d, *J* = 8.4 Hz, 2H), 7.63 (dt, *J* = 6.7, 2.2 Hz, 1H), 7.61 (d, *J* = 7.6 Hz, 2H), 7.46 (t, *J* = 7.6 Hz, 3H), 7.40 – 7.37 (m, 3H), 7.02 (s, 1H), 6.54 (s, 1H), 6.07 – 5.99 (m, 1H), 3.75 (s, 3H). **^{13}C NMR** (176 MHz, DMSO-*d*$_6$) δ 177.46, 147.32, 141.19, 139.90, 137.53, 131.49, 129.83, 129.55, 129.43, 129.32, 128.21, 127.20, 127.10, 124.02, 123.73, 121.79, 120.93, 107.95, 60.97, 36.98. **HRMS**-ESI (m/z): [M+H]$^+$ calculated for $C_{28}H_{22}N_3O_5$ [M+H]$^+$ 480.1559; found, 480.1557.

1-([1,1'-biphenyl]-3-yl)-3-hydroxy-5-(4-nitrophenyl)-4-(thiazole-2-carbonyl)-1,5-dihydro-2*H*-pyrrol-2-one (38):

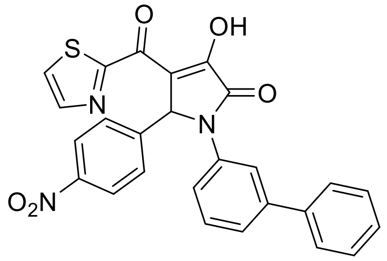

4-Nitrobenzaldehyde (44.65 mg, 0.30 mmol, 1.00 equiv.) followed by the 3-Aminobiphenyl (50.00 mg, 0.30 mmol, 1.00 equiv.) were added to a suspension (5 mL, 0.06 M) of Ethyl-4-(5-methylfuran-2-yl)-2,4-dioxobutanoate (**95l**) (83.93 mg, 0.30 mmol, 1.00 equiv.) in acetic acid following the general procedure to give the desired product as a yellow solid (96.3 mg, 60%).

^{1}H NMR (600 MHz, DMSO-*d*$_6$) δ 8.06 (d, *J* = 8.9 Hz, 2H), 7.97 (t, *J* = 1.9 Hz, 1H), 7.75 (d, *J* = 8.4 Hz, 2H), 7.66 – 7.60 (m, 4H), 7.47 (t, *J* = 7.7 Hz, 3H), 7.44 – 7.35 (m, 4H) 6.59 (s, 1H). **^{13}C NMR** (151 MHz, DMSO-*d*$_6$) δ 169.77, 147.47, 142.76, 141.25, 139.76, 129.92, 129.45,

128.29, 128.01, 127.22, 127.09, 124.55, 123.71, 122.05, 121.89, 121.24, 118.49, 117.73, 59.90, 55.39, 46.23. **LCMS-ESI** (m/z) calculated for $C_{26}H_{18}N_3O_5S$ $[M+H]^+$: 484.1; found:483.8.

1-([1,1'-biphenyl]-3-yl)-3-hydroxy-5-(4-nitrophenyl)-4-(4-(trifluoromethyl)benzoyl)-1,5-dihydro-2*H*-pyrrol-2-one (39):

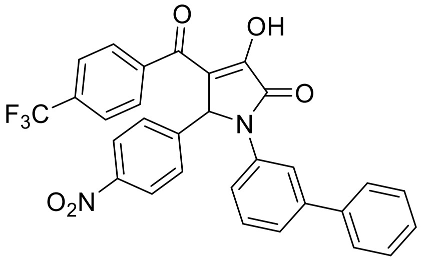

4-Nitrobenzaldehyde (44.65 mg, 0.30 mmol, 1.00 equiv.) followed by the 3-Aminobiphenyl (50.00 mg, 0.30 mmol, 1.00 equiv.) were added to a suspension (5 mL, 0.06 M) of Ethyl-2,4-dioxo-4-(4-(trifluoromethyl)phenyl)butanoate (**95p**) (106.45 mg, 0.30 mmol, 1.00 equiv.) in acetic acid following the general procedure to give the desired product as a yellow solid (39.7 mg, 25%). **HRMS**-ESI (m/z): $[M+H]^+$ calculated for $C_{30}H_{20}N_2O_5F_3$ $[M+H]^+$ 545.1324; found, 545.1325.

4-benzoyl-1-(dibenzo[*b*,*d*]furan-2-yl)-3-hydroxy-5-(4-nitrophenyl)-1,5-dihydro-2*H*-pyrrol-2-one (40):

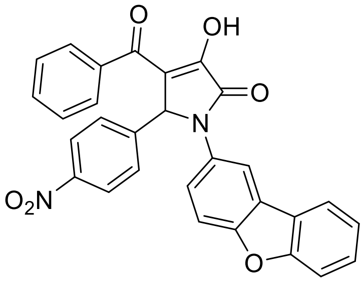

4-Nitrobenzaldehyde (41.24 mg, 0.27 mmol, 1.00 equiv.) followed by the dibenzo[b,d]furan-2-amine (50.00 mg, 0.27 mmol, 1.00 equiv.) were added to a suspension (5 mL, 0.06 M) of Ethyl-2,4-dioxo-4-phenylbutanoate (60.10 mg, 0.27 mmol, 1.00 equiv.) in acetic acid following the general procedure to give the desired product as a yellow solid (52.3 mg, 39%). **^{1}H NMR** (700 MHz, DMSO-d_6) δ 8.41 (s, 1H), 8.10 (d, *J* = 7.7 Hz, 1H), 7.99 (d, *J* = 8.7 Hz, 1H), 7.75 (d, *J* = 66.9 Hz, 4H), 7.67 (s, 3H), 7.52 (t, *J* = 7.7 Hz, 1H), 7.44 (t, *J* = 7.5 Hz, 1H), 7.40 (t, *J* = 7.5 Hz, 1H), 7.37 (t, *J* = 7.6 Hz, 2H), 7.19 (s, 1H), 6.81 (d, *J* = 285.0 Hz, 1H), 6.45 (s, 1H). **^{13}C NMR** (176 MHz, DMSO-d_6) δ 156.40, 153.09, 146.97, 140.32, 132.97, 131.24, 129.46, 129.18, 128.43, 127.89, 124.19, 123.76, 123.67, 123.47, 123.25, 121.72, 115.94,

112.22, 112.14, 61.41, 31.17. **HRMS**-ESI (m/z): $[M+H]^+$ calculated for $C_{29}H_{19}N_2O_6$ $[M+H]^+$ 490.1243; found, 491.1241.

4-(4-bromobenzoyl)-1-(dibenzo[*b,d*]furan-2-yl)-3-hydroxy-5-(4-nitrophenyl)-1,5-dihydro-2*H*-pyrrol-2-one (41):

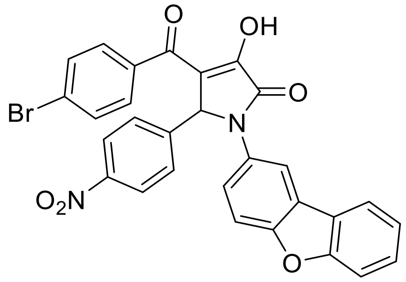

4-Nitrobenzaldehyde (41.24 mg, 0.27 mmol, 1.00 equiv.) followed by the dibenzo[b,d]furan-2-amine (50.00 mg, 0.27 mmol, 1.00 equiv.) were added to a suspension (5 mL, 0.06 M) of Ethyl-ethyl 4-(4-bromophenyl)-2,4-dioxobutanoate (136.06 mg, 0.27 mmol, 1.00 equiv.) in acetic acid following the general procedure to give the desired product as a yellow solid (35.6 mg, 23%).

^{1}H NMR (700 MHz, DMSO-*d*$_6$) δ 12.52 (s, 1H), 8.40 (s, 1H), 8.09 (d, *J* = 7.7 Hz, 1H), 8.01 (d, *J* = 8.3 Hz, 2H), 7.83 – 7.69 (m, 5H), 7.66 (dd, *J* = 11.7, 8.4 Hz, 2H), 7.61 (d, *J* = 8.1 Hz, 2H), 7.52 (t, *J* = 7.7 Hz, 1H), 7.40 (t, *J* = 7.5 Hz, 1H), 6.50 (s, 1H). **^{13}C NMR** (176 MHz, DMSO-*d*$_6$) δ 186.58, 172.47, 156.41, 153.24, 147.47, 147.24, 138.66, 132.50, 131.23, 130.62, 129.58, 128.96, 128.48, 125.74, 124.25, 123.69, 123.65, 123.42, 121.72, 120.16, 116.17, 113.29, 112.24, 61.34, 21.52. **HRMS**-ESI (m/z): $[M+H]^+$ calculated for $C_{29}H_{18}N_2O_6Br$ $[M+H]^+$ 569.0348; found, 569.0350.

1-(dibenzo[*b,d*]furan-2-yl)-3-hydroxy-4-(4-methoxybenzoyl)-5-(4-nitrophenyl)-1,5-dihydro-2*H*-pyrrol-2-one (42):

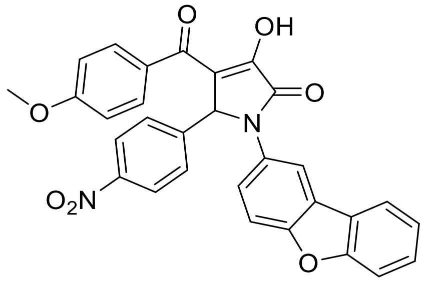

4-Nitrobenzaldehyde (41.24 mg, 0.27 mmol, 1.00 equiv.) followed by the dibenzo[b,d]furan-2-amine (50.00 mg, 0.27 mmol, 1.00 equiv.) were added to a suspension (5 mL, 0.06 M) of

ethyl Ethyl-4-(4-methoxyphenyl)-2,4-dioxobutanoate (**95d**) (97.57 mg, 0.27 mmol, 1.00 equiv.) in acetic acid following the general procedure to give the desired product as a yellow solid (23.2 mg, 16%).

^{1}H NMR (700 MHz, DMSO-*d*$_6$) δ 12.27 (s, 1H), 8.39 (s, 1H), 8.10 (d, *J* = 7.7 Hz, 1H), 8.01 – 7.85 (m, 2H), 7.82 – 7.57 (m, 6H), 7.52 (t, *J* = 7.9 Hz, 1H), 7.39 (t, *J* = 7.3 Hz, 1H), 7.28 (s, 1H), 6.92 – 6.81 (m, 2H), 6.54 (d, *J* = 268.2 Hz, 1H), 3.78 (s, 3H). **^{13}C NMR** (176 MHz, DMSO-*d*$_6$) δ 161.78, 156.40 (2C), 153.44, 153:89, 146.73, 133.46, 132.68, 132.29, 131.33, 130.5, 129.28, 128.53, 124.19, 123.66, 123.26, 123.06, 121.69, 116.51, 115.63, 113.62, 113.27, 112.76, 112.25, 61.47, 55.69. . **HRMS**-ESI (m/z): $[M+H]^+$ calculated for $C_{30}H_{21}N_2O_7$ $[M+H]^+$ 521.1349; found, 521.1346.

1-(dibenzo[*b*,*d*]furan-2-yl)-4-(4-fluorobenzoyl)-3-hydroxy-5-(4-nitrophenyl)-1,5-dihydro-2*H*-pyrrol-2-one (43):

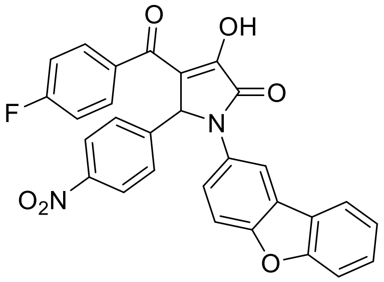

4-Nitrobenzaldehyde (41.24 mg, 0.27 mmol, 1.00 equiv.) followed by the dibenzo[b,d]furan-2-amine (50.00 mg, 0.27 mmol, 1.00 equiv.) were added to a suspension (5 mL, 0.06 M) of Ethyl-4-(4-fluorophenyl)-2,4-dioxobutanoate (**95h**) (92.87 mg, 0.27 mmol, 1.00 equiv.) in acetic acid following the general procedure to give the desired product as a yellow solid (26.8 mg, 19%).

^{1}H NMR (700 MHz, DMSO-*d*$_6$) δ 12.50 (s, 1H), 8.40 (s, 1H), 8.09 (d, *J* = 7.7 Hz, 1H), 8.04 – 7.58 (m, 9H), 7.52 (t, *J* = 7.8 Hz, 1H), 7.39 (t, *J* = 7.5 Hz, 1H), 7.18 (t, *J* = 8.5 Hz, 2H), 6.43 (s, 1H). **^{13}C NMR** (176 MHz, DMSO-*d*$_6$) δ 184.85, 163.36, 156.40, 153.09, 146.93, 136.82, 132.94, 131.85, 130.14, 129.42, 128.42, 124.19, 123.75, 123.67, 123.44, 123.23, 121.70, 115.92, 114.74, 112.22, 112.14, 61.43, 14.51. **HRMS**-ESI (m/z): $[M+H]^+$ calculated for $C_{29}H_{18}FN_2O_6$ $[M+H]^+$ 509.1071; found, 509.1048.

1-(dibenzo[*b*,*d*]furan-2-yl)-4-(furan-2-carbonyl)-3-hydroxy-5-(4-nitrophenyl)-1,5-dihydro-2*H*-pyrrol-2-one (44):

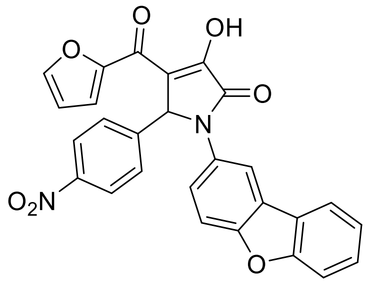

4-Nitrobenzaldehyde (41.24 mg, 0.27 mmol, 1.00 equiv.) followed by the dibenzo[*b,d*]furan-2-amine (50.00 mg, 0.27 mmol, 1.00 equiv.) were added to a suspension (5 mL, 0.06 M) of Ethyl-4-(furan-2-yl)-2,4-dioxobutanoate (**95c**) (81.95 mg, 0.27 mmol, 1.00 equiv.) in acetic acid following the general procedure to give the desired product as a yellow solid (80.6 mg, 61%).

^{1}H NMR (700 MHz, DMSO-*d*$_6$) δ 8.71 (s, 1H), 8.38 (s, 1H), 8.12 (d, *J* = 7.7 Hz, 1H), 7.87 (d, *J* = 107.7 Hz, 3H), 7.56 (d, *J* = 231.7 Hz, 9H), 6.56 (d, *J* = 377.1 Hz, 2H). **^{13}C NMR** (176 MHz, DMSO-*d*$_6$) δ 170.76, 154.27, 151.46, 150.56, 146.38, 144.68, 142.34, 131.48, 127.48, 127.29, 126.35, 122.11, 121.57, 121.02, 119.63, 115.44, 113.68, 113.41, 110.76, 110.09, 109.43, 58.92, 53.26. **HRMS**-ESI (m/z): $[M+H]^+$ calculated for $C_{27}H_{16}N_2O_7$ $[M+H]^+$ 481.1036; found, 481.1032.

4-benzoyl-1-(dibenzo[*b,d*]furan-3-yl)-3-hydroxy-5-(4-nitrophenyl)-1,5-dihydro-2*H*-pyrrol-2-one (45):

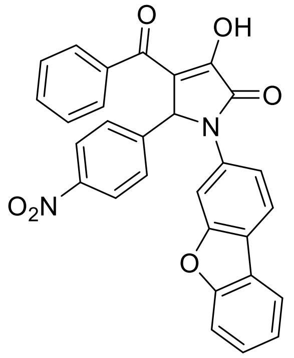

4-Nitrobenzaldehyde (34.3 mg, 0.23 mmol, 1.00 equiv.) followed by the dibenzo[*b,d*]furan-3-amine (41.6 mg, 0.23 mmol, 1.00 equiv.) were added to a suspension (3 mL, 0.06 M) of ethyl 3-oxo-3-phenylpropanoate (50 mg, 0.23 mmol, 1.00 equiv.) in acetic acid following the general procedure to give the desired product as a pale yellow solid (30.1 mg, 0.06 mmol, 27%).

^{1}H NMR (700 MHz, DMSO-*d*$_6$) δ 11.66 (d, *J* = 421.9 Hz, 1H), 8.10 – 8.01 (m, 5H), 7.82 (d, *J* = 8.9 Hz, 2H), 7.75 (d, *J* = 6.8 Hz, 2H), 7.72 (dd, *J* = 8.3, 1.9 Hz, 1H), 7.67 (d, *J* = 8.2 Hz, 1H),

7.59 (d, J = 17.6 Hz, 1H), 7.48 (dt, J = 13.5, 7.5 Hz, 3H), 7.38 (t, J = 7.5 Hz, 1H), 6.68 (s, 1H). **^{13}C NMR** (176 MHz, DMSO-d_6) δ 189.56, 165.18, 156.34, 155.77, 147.62, 144.97, 138.31, 135.95, 133.27, 130.24, 129.77, 129.23, 128.68, 128.00, 124.57, 123.92, 123.80, 123.51, 121.65, 121.50, 121.47, 119.80, 118.46, 112.06, 106.53, 104.67, 61.23. **HRMS**-ESI (m/z): $[M+H]^+$ calculated for $C_{29}H_{19}N_2O_6$ $[M+H]^+$ 491.1243; found, 491.1243.

4-(4-bromobenzoyl)-1-(dibenzo[*b,d*]furan-3-yl)-3-hydroxy-5-(4-nitrophenyl)-1,5-dihydro-2*H*-pyrrol-2-one (46):

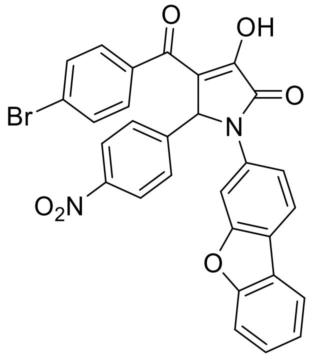

4-Nitrobenzaldehyde (25.3 mg, 0.17 mmol, 1.00 equiv.) followed by the dibenzo[*b,d*]furan-3-amine (30.6 mg, 0.17 mmol, 1.00 equiv.) were added to a suspension (3 mL, 0.06 M) of ethyl 3-(4-bromophenyl)-3-oxopropanoate (50 mg, 0.17 mmol, 1.00 equiv.) in acetic acid following the general procedure to give the desired product as a yellow solid (24.7 mg, 0.04 mmol, 26%). **^{1}H NMR** (600 MHz, DMSO-d_6) δ 12.49 (s, 1H), 8.10 – 8.02 (m, 5H), 7.84 – 7.80 (m, 2H), 7.73 – 7.64 (m, 6H), 7.49 (ddd, J = 8.4, 7.2, 1.4 Hz, 1H), 7.38 (td, J = 7.5, 0.9 Hz, 1H), 6.66 (s, 1H). **^{13}C NMR** (176 MHz, DMSO-d_6) δ 188.50, 165.06, 156.35, 155.76, 152.04, 147.64, 144.94, 137.42, 135.89, 131.77, 131.19, 129.80, 128.01, 127.15, 123.91, 123.81, 123.50, 121.66, 121.52, 119.32, 118.48, 112.07, 106.55, 61.13. **HRMS**-ESI (m/z): $[M+H]^+$ calculated for $C_{29}H_{18}N_2O_6Br$ $[M+H]^+$ 569.0348; found, 569.0350.

1-(dibenzo[*b,d*]furan-3-yl)-3-hydroxy-4-(4-methoxybenzoyl)-5-(4-nitrophenyl)-1,5-dihydro-2*H*-pyrrol-2-one (47):

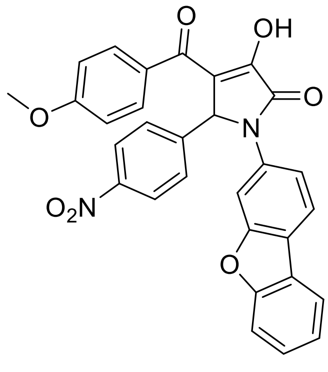

4-Nitrobenzaldehyde (30.2 mg, 0.2 mmol, 1.00 equiv.) followed by the dibenzo[*b*,*d*]furan-3-amine (36.6 mg, 0.2 mmol, 1.00 equiv.) were added to a suspension (3 mL, 0.06 M) of ethyl 3-(4-methoxyphenyl)-3-oxopropanoate (**95d**) (50 mg, 0.2 mmol, 1.00 equiv.) in acetic acid following the general procedure to give the desired product as a yellow solid (33.3 mg, 0.06 mmol, 32%).

^{1}H NMR (700 MHz, DMSO-d_6) δ 11.97 (s, 1H), 8.10 (s, 1H), 8.08 – 7.99 (m, 2H), 7.93 (dd, *J* = 15.2, 8.4 Hz, 3H), 7.73 (d, *J* = 8.6 Hz, 2H), 7.63 (d, *J* = 8.3 Hz, 1H), 7.58 (d, *J* = 8.4 Hz, 1H), 7.51 – 7.44 (m, 1H), 7.36 (t, *J* = 7.5 Hz, 1H), 7.04 – 6.70 (m, 3H), 6.41 (s, 1H), 3.75 (s, 3H). **^{13}C NMR** (176 MHz, DMSO-d_6) δ 184.61, 171.09, 161.34, 156.26, 155.89, 150.17, 146.58, 137.26, 133.57, 131.18, 129.21, 127.71, 123.70, 123.65, 123.21, 121.51, 121.38, 121.37, 121.32, 120.69, 117.72, 113.65, 112.78, 112.30, 111.98, 105.60, 61.52, 55.59. **HRMS**-ESI (m/z): $[M+H]^+$ calculated for $C_{30}H_{21}N_2O_7$ $[M+H]^+$ 521.1349; found, 521.1348.

1-(dibenzo[*b*,*d*]furan-3-yl)-4-(4-fluorobenzoyl)-3-hydroxy-5-(4-nitrophenyl)-1,5-dihydro-2*H*-pyrrol-2-one (48):

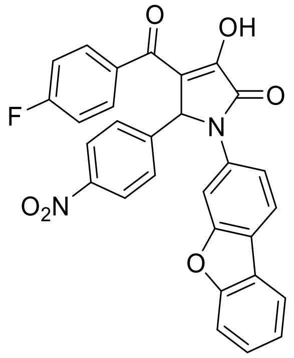

4-Nitrobenzaldehyde (31.7 mg, 0.21 mmol, 1.00 equiv.) followed by the dibenzo[*b*,*d*]furan-3-amine (38.5 mg, 0.21 mmol, 1.00 equiv.) were added to a suspension (3 mL, 0.06 M) of ethyl 3-(4-fluorophenyl)-3-oxopropanoate (**95h**) (50 mg, 0.17 mmol, 1.00 equiv.) in acetic acid following the general procedure to give the desired product as a yellow solid (34.2 mg, 0.07 mmol, 32%).

^{1}H NMR (600 MHz, DMSO-d_6) δ 12.36 (s, 1H), 8.09 – 8.02 (m, 5H), 7.87 – 7.80 (m, 4H), 7.71 (dd, *J* = 8.5, 1.8 Hz, 1H), 7.67 (d, *J* = 8.2 Hz, 1H), 7.49 (ddd, *J* = 8.4, 7.2, 1.4 Hz, 1H), 7.38 (td, *J* = 7.5, 1.0 Hz, 1H), 7.34 – 7.27 (m, 2H), 6.67 (s, 1H). **^{13}C NMR** (151 MHz, DMSO-d_6) δ 188.04, 166.10, 165.11, 164.43, 156.35, 155.77, 151.51, 147.64, 144.93, 135.92, 134.90, 132.23, 132.17, 129.79, 128.00, 123.92, 123.80, 123.50, 121.65, 121.50, 121.49, 119.58, 118.47, 115.84, 115.70, 112.06, 106.53, 61.21. **HRMS**-ESI (m/z): $[M+H]^+$ calculated for $C_{29}H_{18}FN_2O_6$ $[M+H]^+$ 509.1104; found, 509.1148.

1-(dibenzo[*b*,*d*]furan-3-yl)-4-(furan-2-carbonyl)-3-hydroxy-5-(4-nitrophenyl)-1,5-dihydro-2*H*-pyrrol-2-one (49):

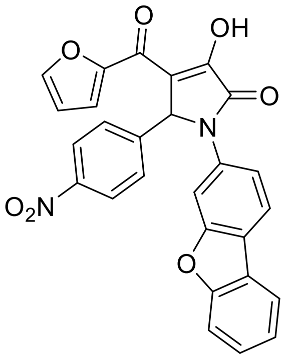

4-Nitrobenzaldehyde (35.9 mg, 0.24 mmol, 1.00 equiv.) followed by the dibenzo[*b*,*d*]furan-3-amine (43.6 mg, 0.24 mmol, 1.00 equiv.) were added to a suspension (3 mL, 0.06 M) of ethyl 3-(furan-2-yl)-3-oxopropanoate (**95c**) (50 mg, 0.24 mmol, 1.00 equiv.) in acetic acid following the general procedure to give the desired product as a yellow solid (39.0 mg, 0.08 mmol, 34%). **^{1}H NMR** (700 MHz, DMSO-d_6) δ 11.96 (s, 1H), 8.61 (d, *J* = 41.1 Hz, 1H), 8.30 – 7.95 (m, 4H), 7.94 – 7.76 (m, 2H), 7.75 – 7.69 (m, 1H), 7.65 (s, 1H), 7.58 (d, *J* = 8.8 Hz, 2H), 7.47 (s, 1H), 7.40 – 7.31 (m, 1H), 6.99 – 6.60 (m, 1H), 6.59 – 6.27 (m, 1H). **^{13}C NMR** (176 MHz, DMSO-d_6) δ 172.48, 162.78, 156.29, 155.85, 154.12, 152.54, 146.94, 146.53, 144.87, 137.31, 136.29, 129.66, 129.38, 128.03, 127.69, 123.70, 123.07, 121.39, 120.62, 117.73, 116.24, 112.90, 112.01, 111.73, 111.53, 105.58, 46.08. **HRMS**-ESI (m/z): $[M+H]^+$ calculated for $C_{27}H_{16}N_2O_7$ $[M+H]^+$ 481.1036; found, 481.1033.

General Procedure for Butanoate Synthesis

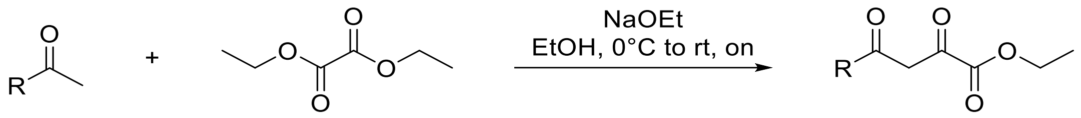

Scheme S2.

Ketone (1.00 equiv.) followed by diethyl oxalate (1.00 equiv.) were added to a solution of sodium ethoxide (1.00M, 1.10 equiv.) in EtOH (3.5 mL) at 0°C. The resulting suspension was stirred, overnight, at room temperature. Pentane was added and the mixture was filtered. The residue was washed with pentane and if necessary purified by recrystallisation flash column chromatography.

Ethyl 2,4-dioxo-4-(pyridin-2-yl)butanoate (95a)

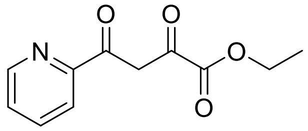

In a round bottom flask, 1-(pyridin-2-yl)ethan-1-one (2.78 mL, 24.8 mmol) was dissolved in THF (50 mL) and the resulting solution cooled to 0 °C. Sodium ethoxide (13.9 mL, 37.2 mmol) was then added dropwise and the reaction allowed to stir for 15 min at 0 °C. Diethyl oxalate (3.70 mL, 27.2 mmol) was finally added dropwise, the cooling bath removed, and the reaction allowed to stir overnight at rt. The reaction was quenched with 1M HCl (50 mL). The resulting suspension was poured into a separatory funnel and the crude product was extracted with DCM (3x). The combined organic layers were dried using a phase separator and solvent was removed under reduced pressure. The residue was purified by automated flash chromatography on a Biotage® KP-SIL 10g column. A gradient from 0 to 10% of EtOAc in heptane over 20 CV was used as mobile phase. Collected fractions were dried under reduced pressure, to give ethyl 2,4-dioxo-4-(pyridin-2-yl)butanoate (2.015 g, 35.8 %) as a yellow solid.

Ethyl-4-(3,4-dimethoxyphenyl)-2,4-dioxobutanoate (95b):

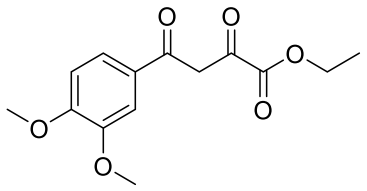

3',4'-Dimethoxyacetophenone (200.00 mg, 1.11 mmol, 1.00 equiv.) was used according to the general procedure and purified by flash column chromatography to yield the desired product

as white powder (279.90 mg, 67%) **LCMS-ESI** (m/z) calculated for $C_{14}H_{17}O_6$ $[M+H]^+$: 281.1; found: 281.1.

Ethyl-4-(furan-2-yl)-2,4-dioxobutanoate (95c):

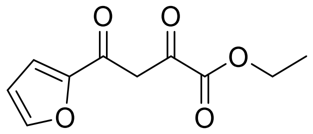

2-Acetylfuran (111.31 µl, 1.11 mmol, 1.00 equiv.) was used according to the general procedure and purified by flash column chromatography to yield the desired product as white powder (151.00 mg, 65%) **LCMS-ESI** (m/z) calculated for $C_{10}H_{11}O_5$ $[M+H]^+$: 211.1; found: 211.1.

Ethyl-4-(4-methoxyphenyl)-2,4-dioxobutanoate (95d):

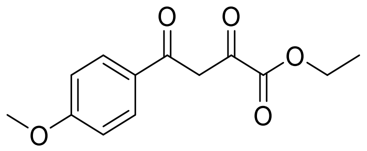

4-Methoxy-Acetophenone (500.00 mg, 3.33 mmol, 1.00 equiv.) was used according to the general procedure and purified by flash column chromatography to yield the desired product as white powder (318.00 mg, 21%) **LCMS-ESI** (m/z) calculated for $C_{13}H_{15}O_5$ $[M+H]^+$: 251.1; found: 251.0.

Ethyl-4-cyclohexyl-2,4-dioxobutanoate (95e):

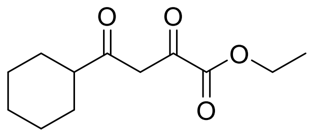

1-Cyclohexylethan-1-one (432.78 µl, 3.33mmol, 1.00 equiv.) was used according to the general procedure and gave the desired product as clear liquid (371.70 mg, 49%) **LCMS-ESI** (m/z) calculated for $C_{12}H_{19}O_4$ $[M+H]^+$: 227.1; found: 227.0.

Ethyl-4-cyclopropyl-2,4-dioxobutanoate (95f):

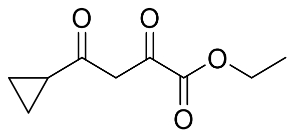

Cyclopropylmethylketone (311,924 µl 3,33mmol 1.00 equiv) was used according to the general procedure and gave the desired product as clear liquid (252.50 mg, 41%) **LCMS-ESI** (m/z) calculated for $C_9H_{13}O_4$ $[M+H]^+$: 207.1; found: 207.1.

Ethyl-4-(4-fluoro-2-hydroxyphenyl)-2,4-dioxobutanoate (95g):

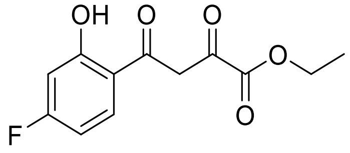

4'-Fluoro-2'hydroxyacetophenone (513.20 mg, 3.33mmol 1.00 equiv.) was used according to the general procedure and purified by flash column chromatography to yield the desired product as white solid (771.40 mg, 36%) **LCMS-ESI** (m/z) calculated for $C_{12}H_{12}FO_5$ $[M+H]^+$: 255.1; found: 255.1.

Ethyl-4-(4-fluorophenyl)-2,4-dioxobutanoate (95h):

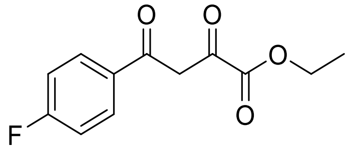

4-Fluoro-acetophenone (403.09 mg, 3.33mmol 1.00 equiv.) was used according to the general procedure and was used without further purification (460.20 mg, 37%) **LCMS-ESI** (m/z) calculated for $C_{12}H_{12}FO_4$ $[M+H]^+$: 239.1; found: 239.3.

Ethyl-4-(3-hydroxy-4-nitrophenyl)-2,4-dioxobutanoate (95i):

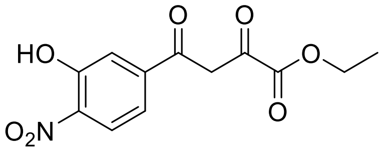

4'-Hydroxy-3'-nitroacetophenone (603.22, 3.33mmol 1.00 equiv.) was used according to the general procedure and was used without further purification (490.20 mg, 26%) **LCMS-ESI** (m/z) calculated for $C_{12}H_{12}NO_7$ $[M+H]^+$: 282.1; found: 282.7.

Ethyl-4-(4-(methylsulfonyl)phenyl)-2,4-dioxobutanoate (95j):

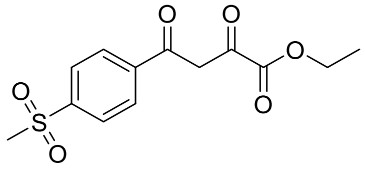

4'-Methylsulphonylacetophenone (660.14, 3.33mmol 1.00 equiv.) was used according to the general procedure and was used without further purification (184.10 mg, 14%) **LCMS-ESI** (m/z) calculated for $C_{13}H_{15}O_6S$ $[M+H]^+$: 299.1; found: 299.5.

Ethyl-4-cyclobutyl-2,4-dioxobutanoate (95k):

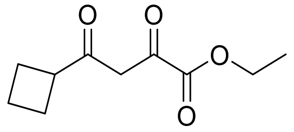

1-Cyclobutylethanone (363.13 µl, 3.33mmol 1.00 equiv.) was used according to the general procedure and was used without further purification (503.10 mg, 76%) **LCMS-ESI** (m/z) calculated for $C_{10}H_{14}O_4Na$ $[M+Na]^+$: 221.1; found: 221.5.

Ethyl-2,4-dioxo-4-(thiazol-2-yl)butanoate (95l):

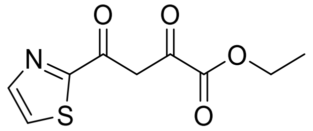

2-Acetylthiazole (345.12 µl, 3.33mmol 1.00 equiv.) according to the general procedure and was used without further purification (524.40 mg, 49%) **LCMS-ESI** (m/z) calculated for $C_9H_{10}NO_4S$ $[M+H]^+$: 228.0; found: 228.0.

Ethyl-4-(5-methylfuran-2-yl)-2,4-dioxobutanoate (95m):

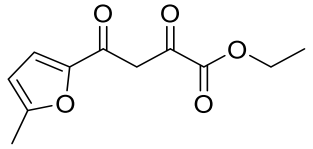

5-Methyl-2-acetylfuran (387.06 µl, 3.33mmol, 1.00 equiv.) according to the general procedure and was used without further purification (672.20 mg, 72%) **LCMS-ESI** (m/z) calculated for $C_{11}H_{13}O_5$ $[M+H]^+$: 225.1; found: 224.8.

Ethyl-4-(1-methyl-1*H*-pyrrol-2-yl)-2,4-dioxobutanoate (95n):

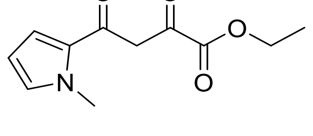

2-Acetyl-1-methylpyrrole (394.33 µl, 3.33mmol, 1.00 equiv.) according to the general procedure and was used without further purification (620.60 mg, 75%) **LCMS-ESI** (m/z) calculated for $C_{11}H_{14}NO_4$ $[M+H]^+$: 224.1; found: 224.0.

Ethyl-4-(2-fluorophenyl)-2,4-dioxobutanoate (95o):

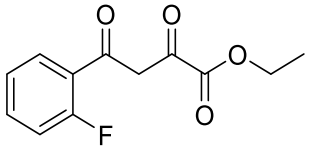

2-Fluoroacetophenone (404.58 µl, 3.33mmol 1.00 equiv.) according to the general procedure and was used without further purification (669.60 mg, 59%) **LCMS-ESI** (m/z) calculated for $C_{12}H_{12}FO_4$ $[M+H]^+$: 239.1; found: 239.2.

Ethyl-2,4-dioxo-4-(4-(trifluoromethyl)phenyl)butanoate (95p):

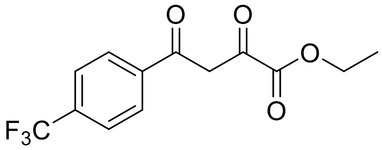

4'-(Trifluoromethyl)acetophenone (522.11 µl, 3.33mmol 1.00 equiv.) according to the general procedure and was used without further purification (669.60 mg, 59%) **LCMS-ESI** (m/z) calculated for $C_{13}H_{12}F_3O_4$ $[M+H]^+$: 289.1; found:289.5.

NMR Spectra

^{1}H NMR of 3 (500 MHz, DMSO-d_6):

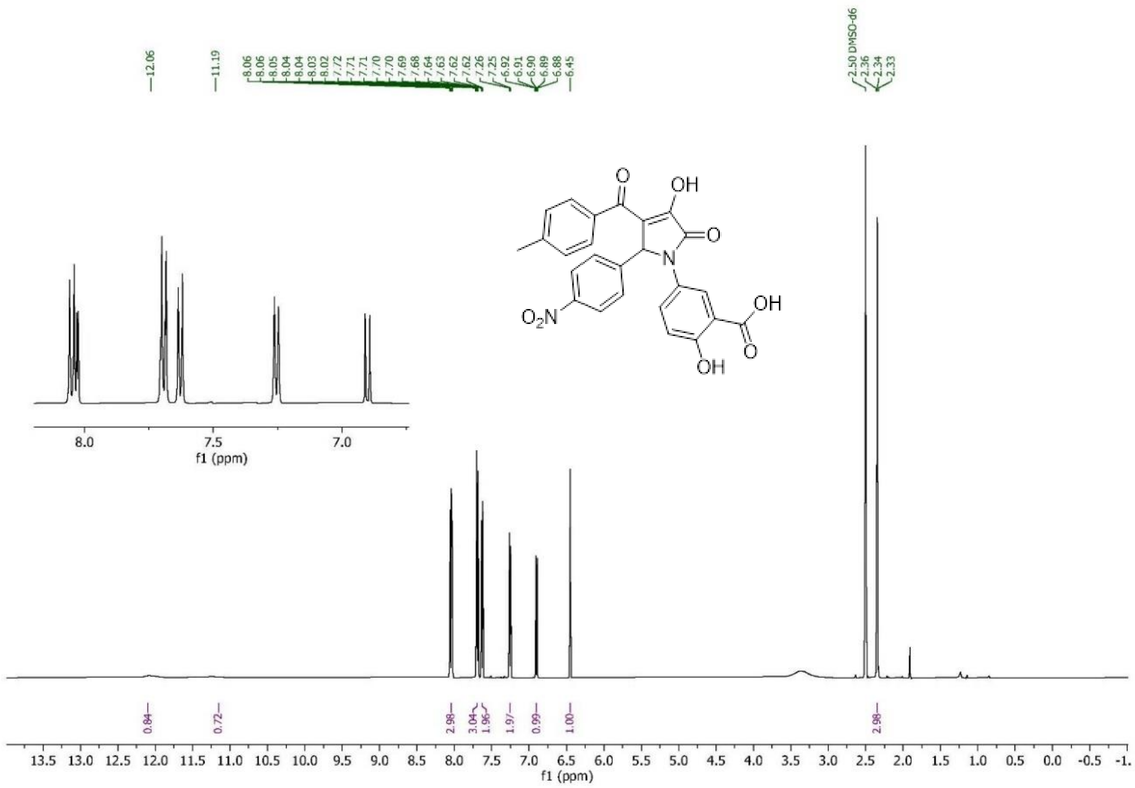

^{13}C NMR of 3 (126 MHz, DMSO-d_6):

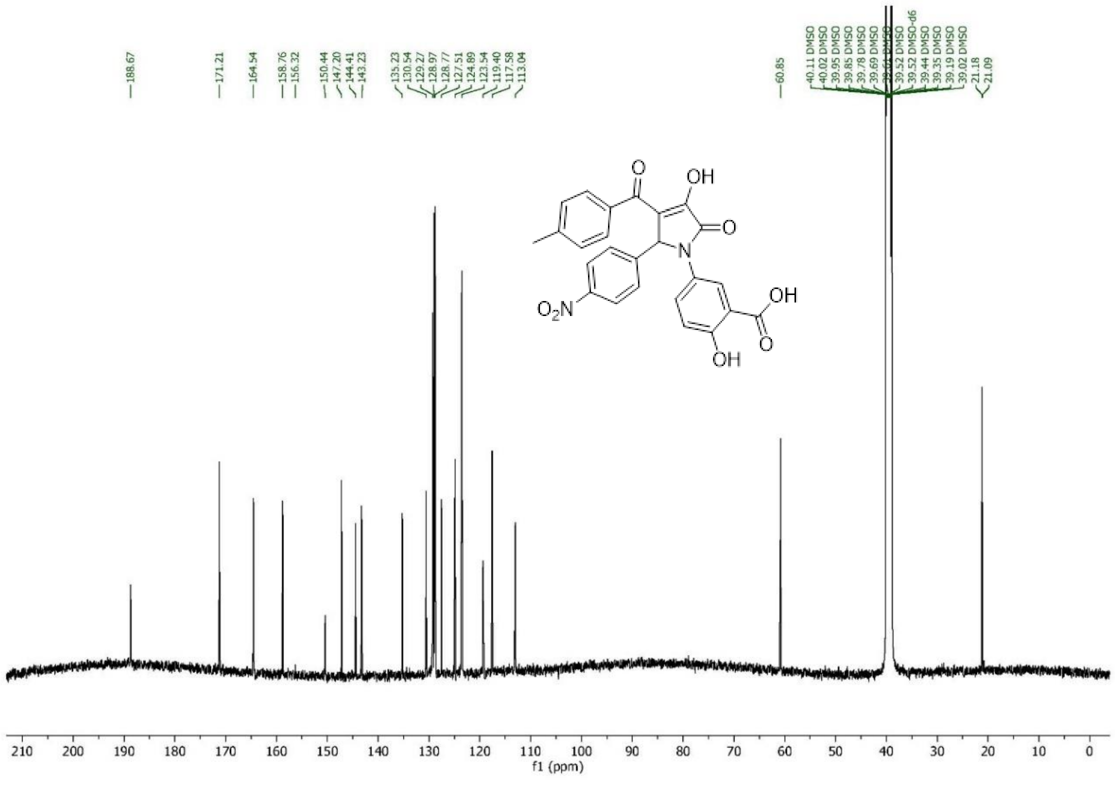

^{1}H NMR of 4 (500 MHz, DMSO-d_6):

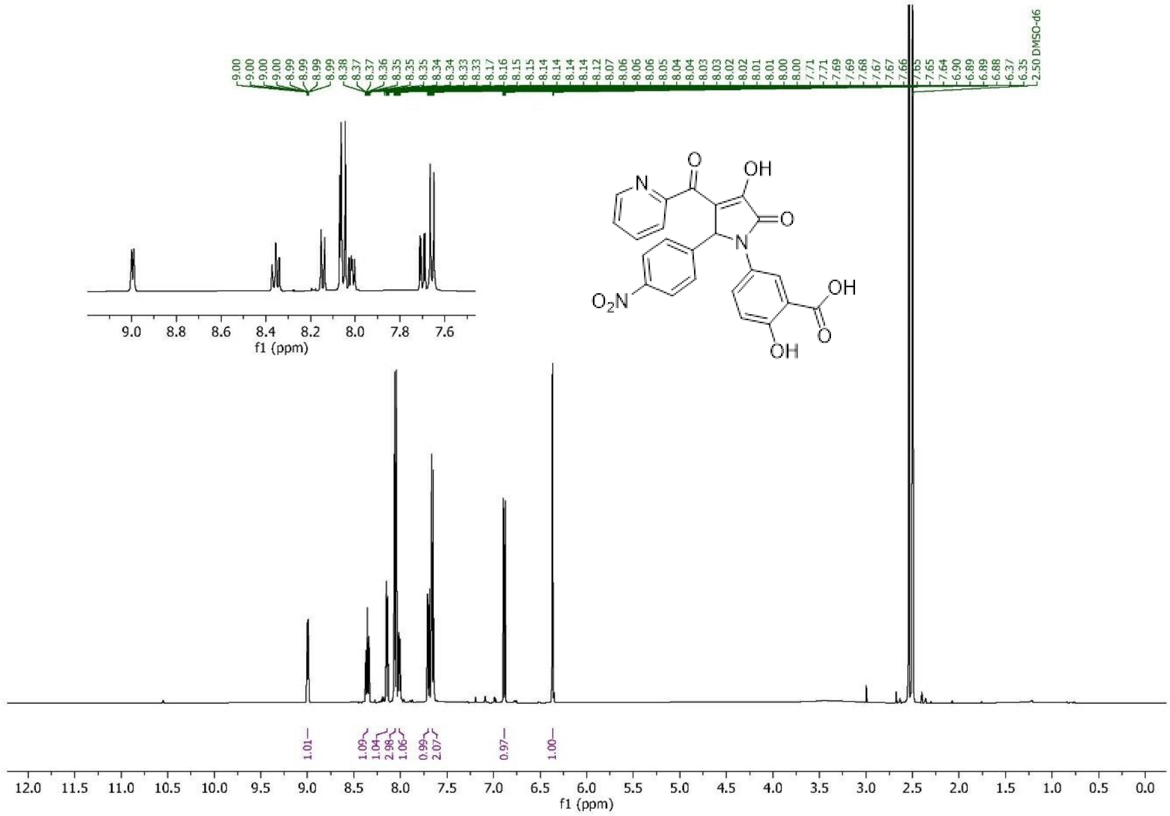

^{13}C NMR of 4 (126 MHz, DMSO-d_6):

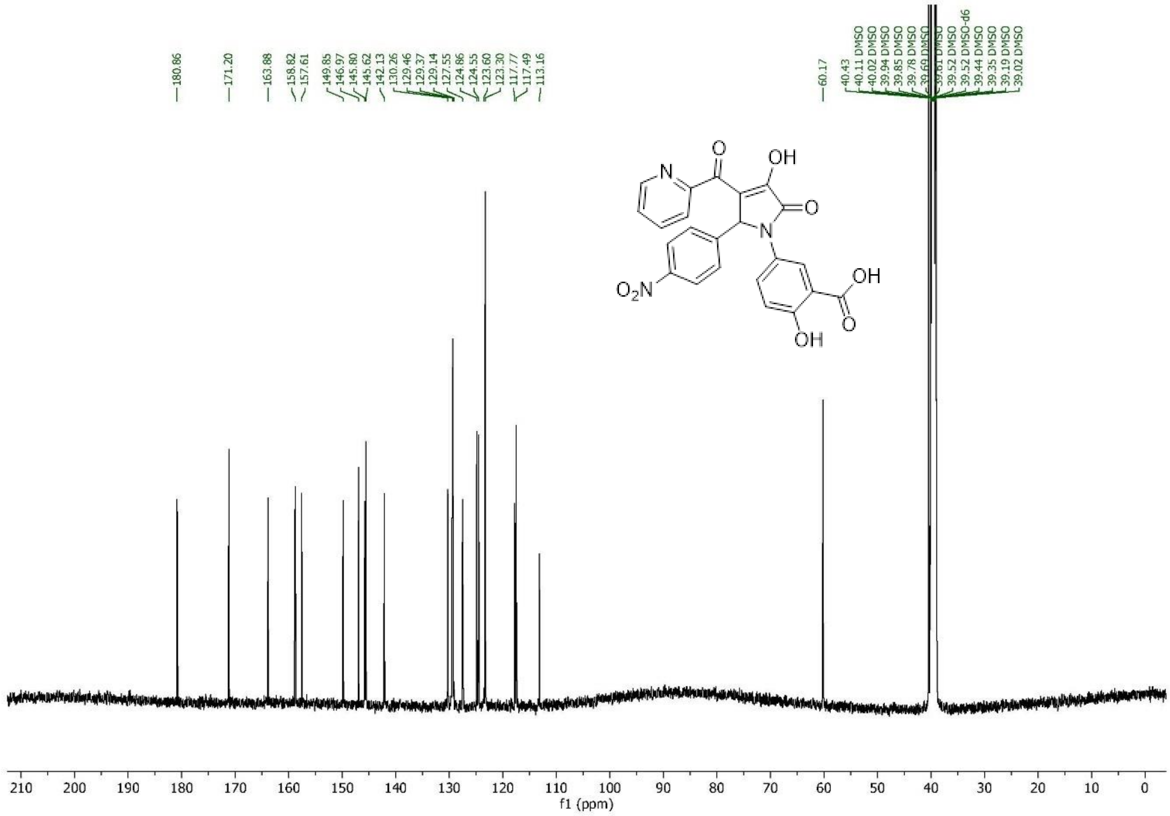

^{1}H NMR of 5 (500 MHz, DMSO-d_6):

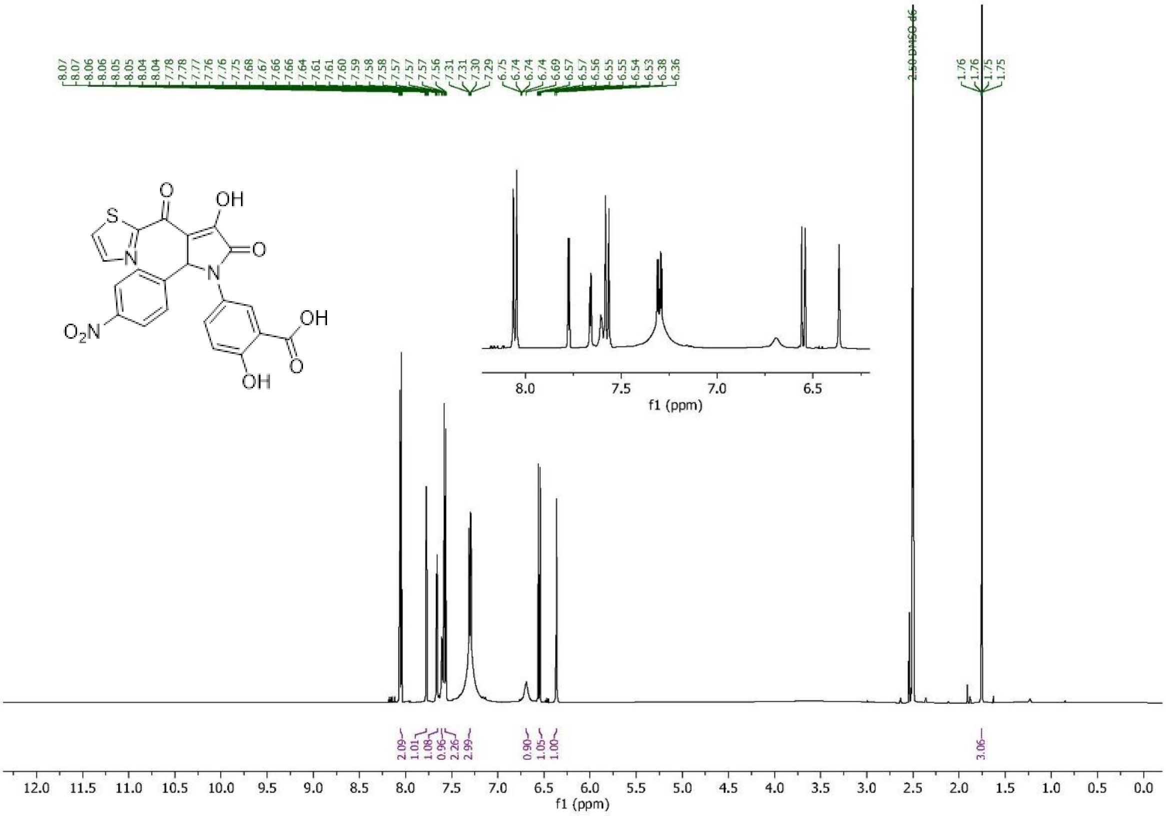

^{13}C NMR of 5 (126 MHz, DMSO-d_6):

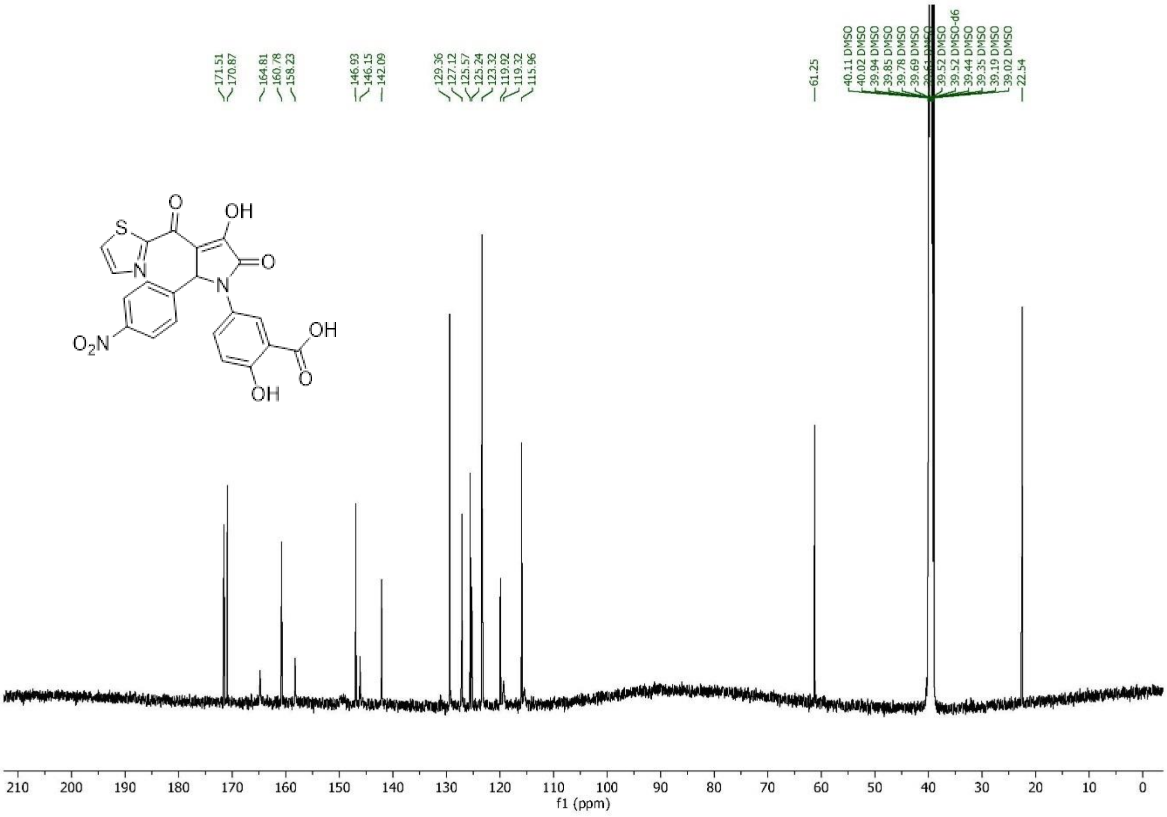

¹H NMR of 6 (500 MHz, DMSO-d_6):

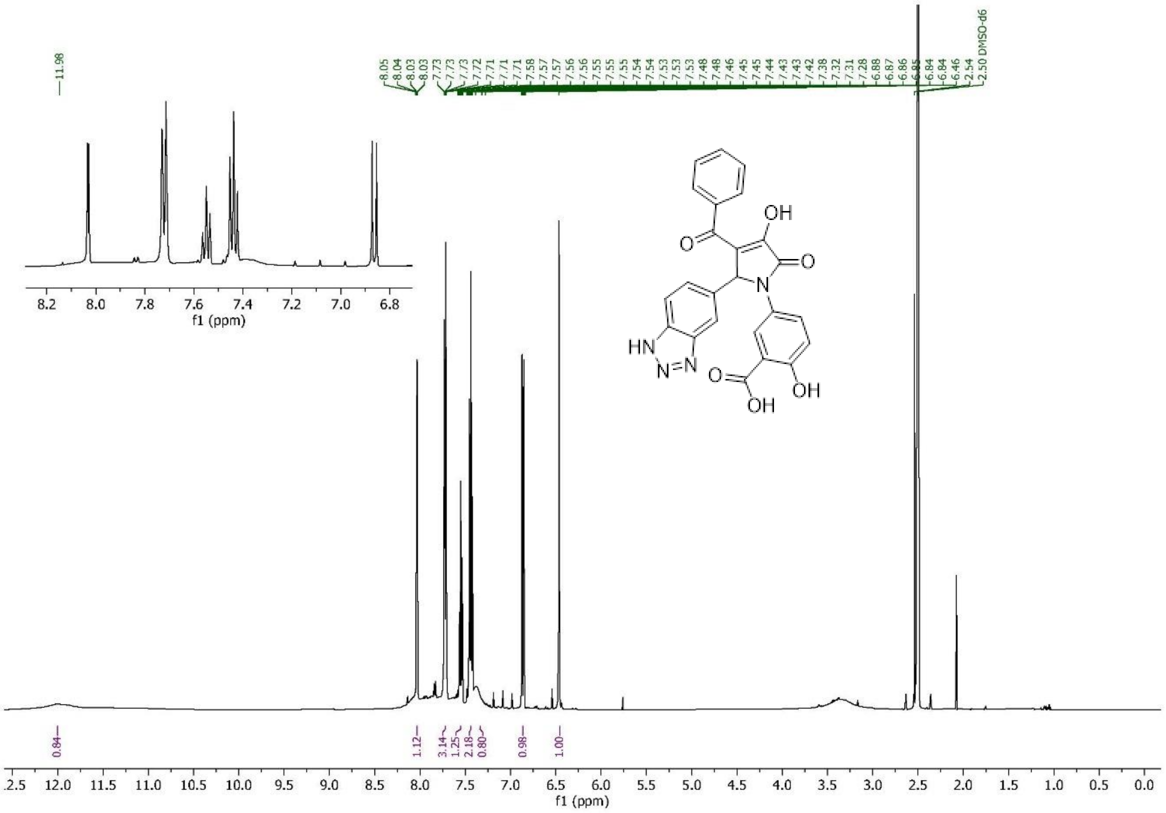

¹³C NMR of 6 (126 MHz, DMSO-d_6):

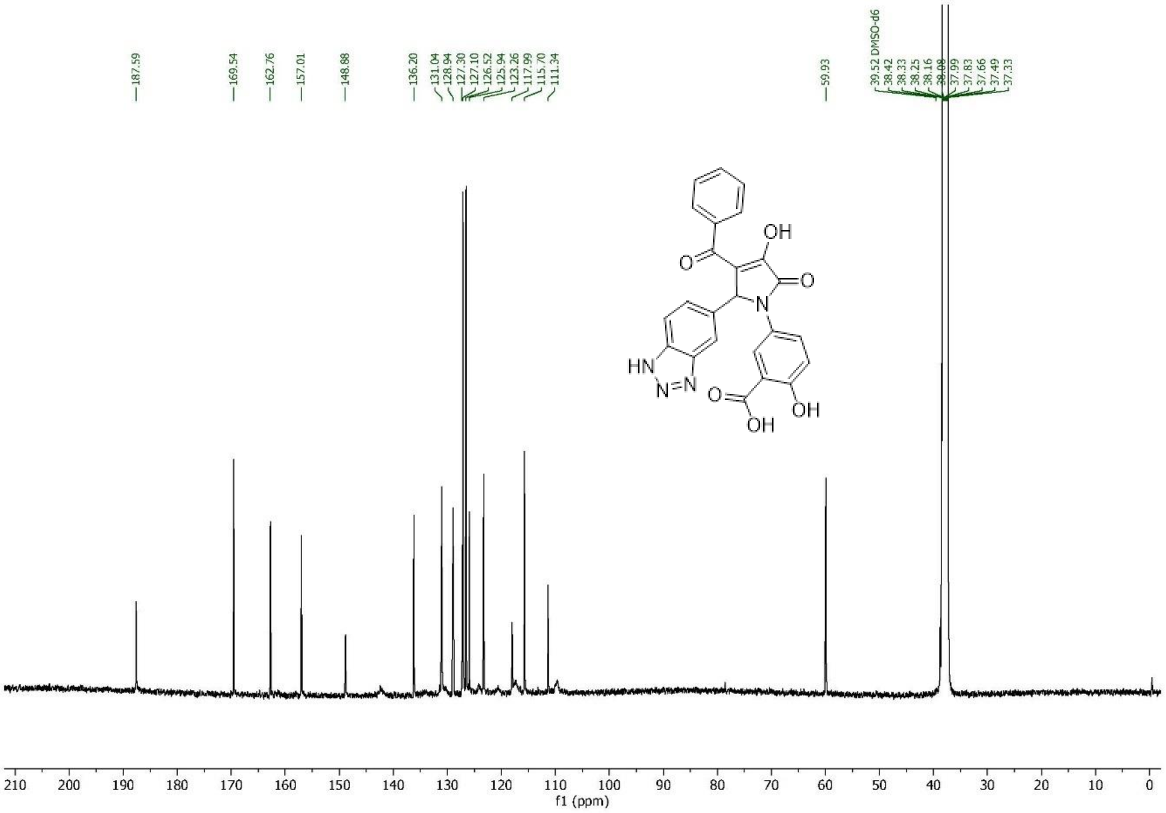

^{1}H NMR of 7 (500 MHz, DMSO-d_6):

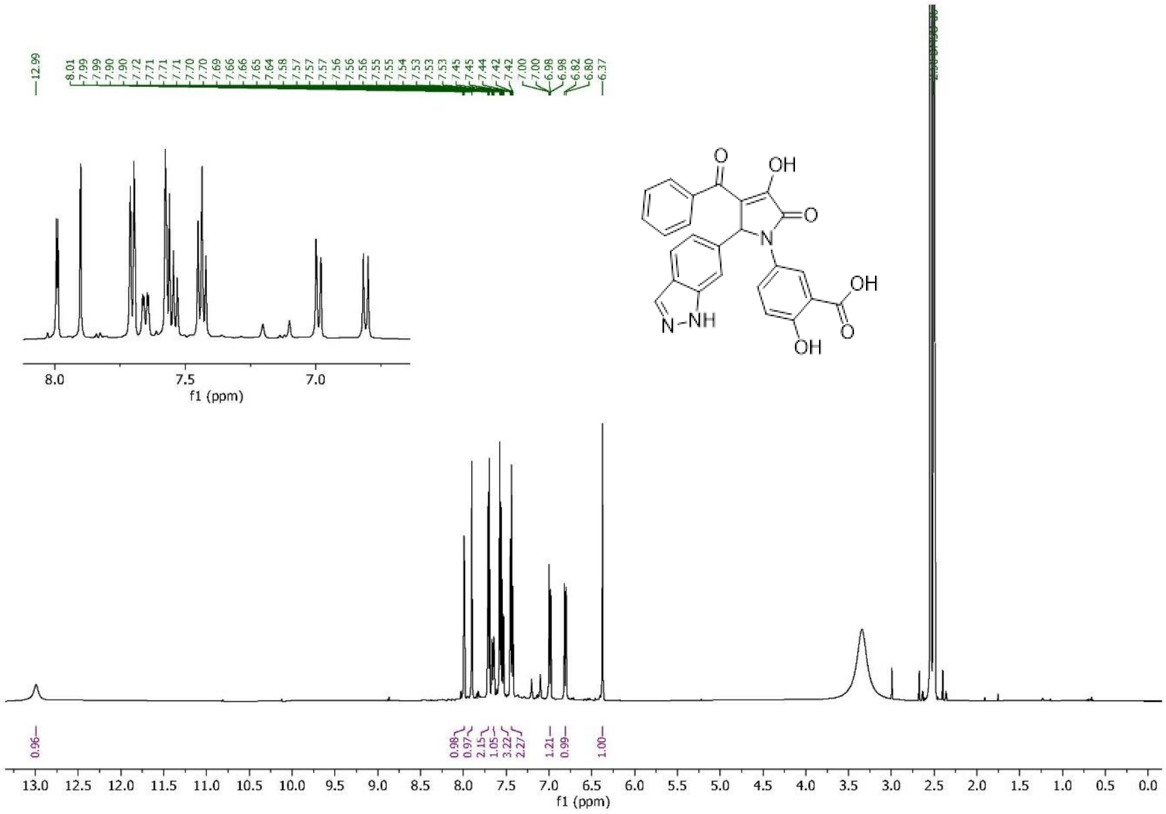

^{13}C NMR of 7 (126 MHz, DMSO-d_6):

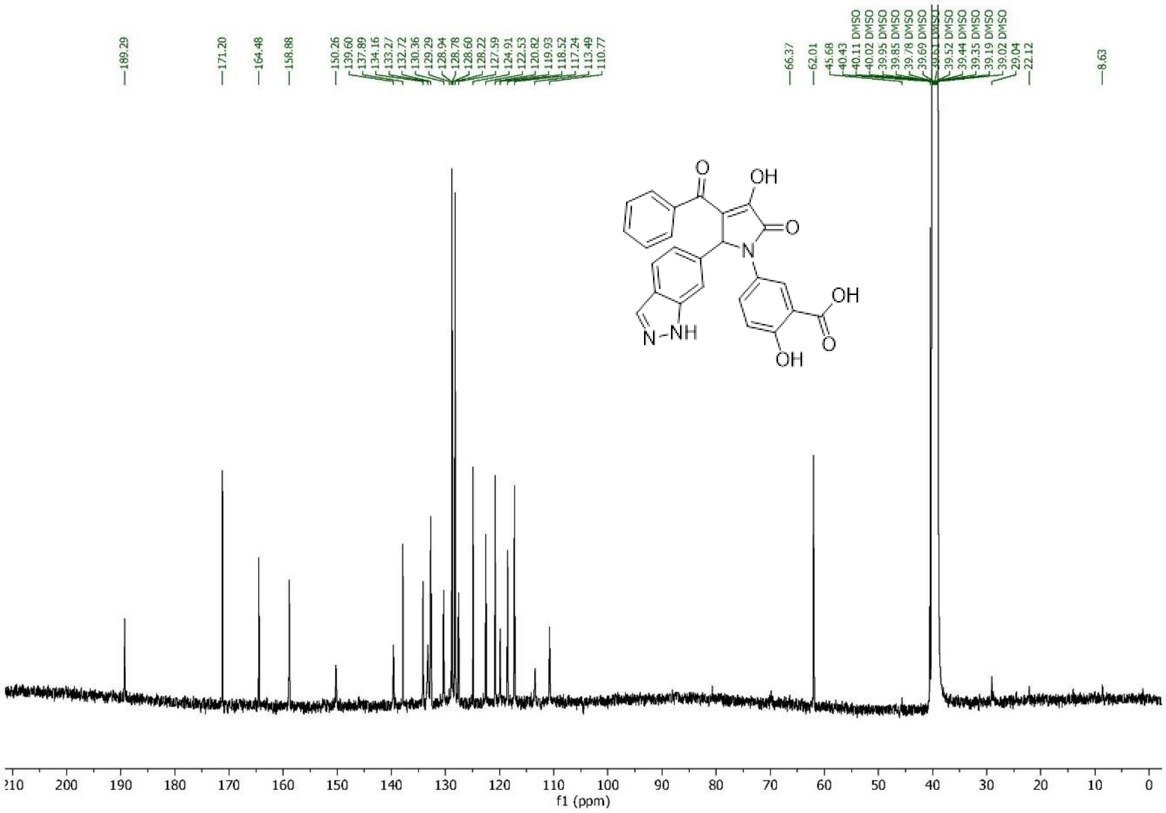

^{1}H NMR of 8 (500 MHz, DMSO-d_6):

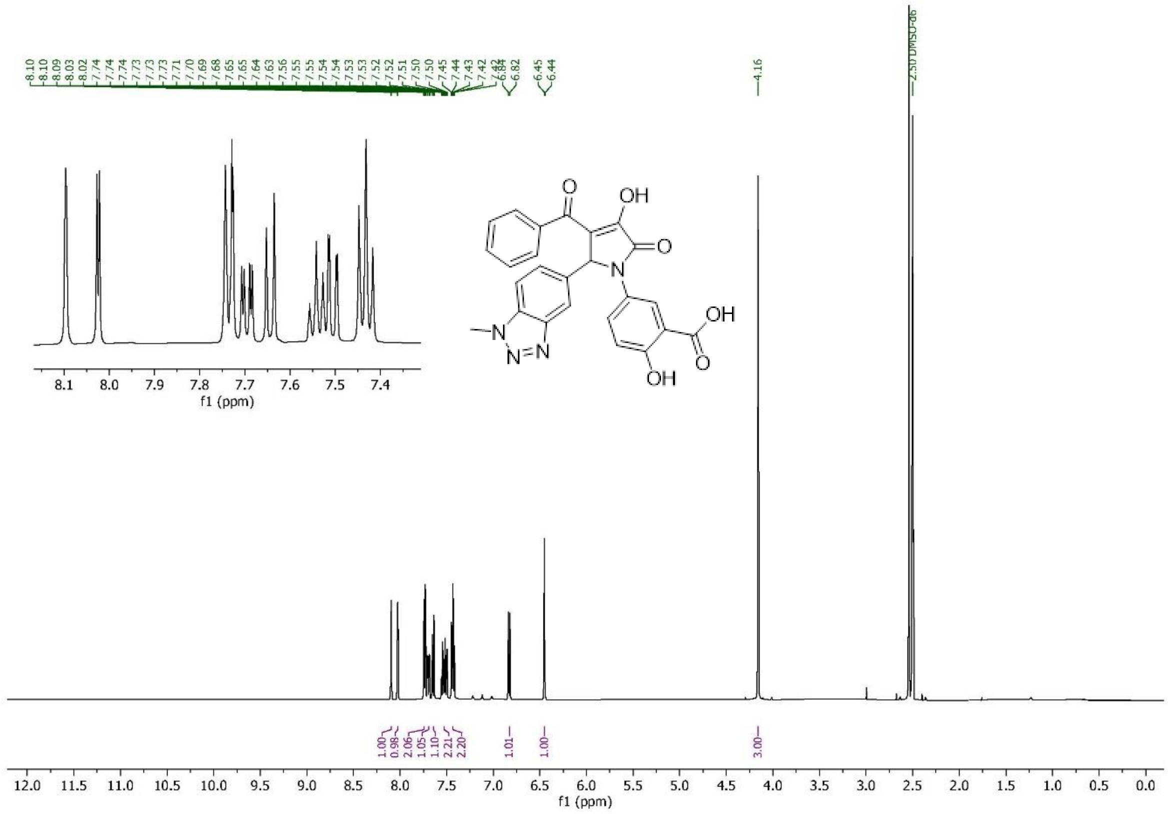

^{13}C NMR of 8 (126 MHz, DMSO-d_6):

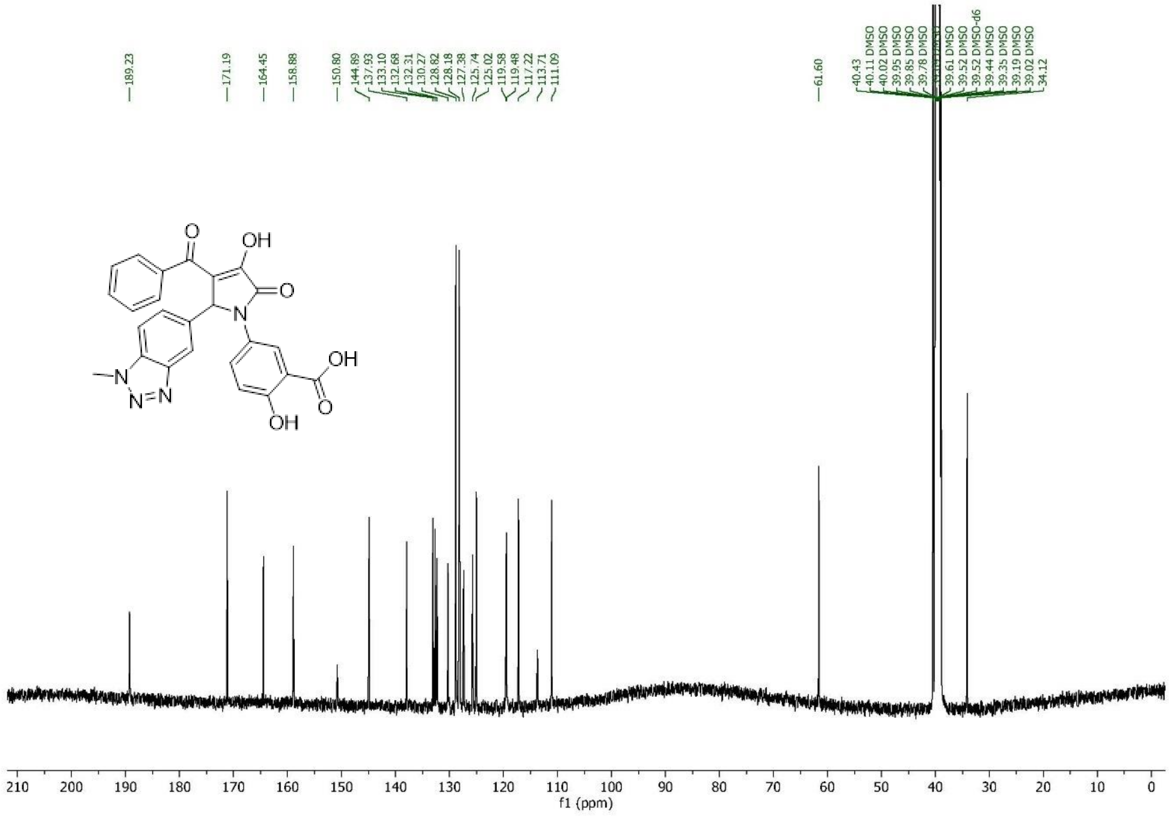

^{1}H NMR of 9 (500 MHz, DMSO-d_6):

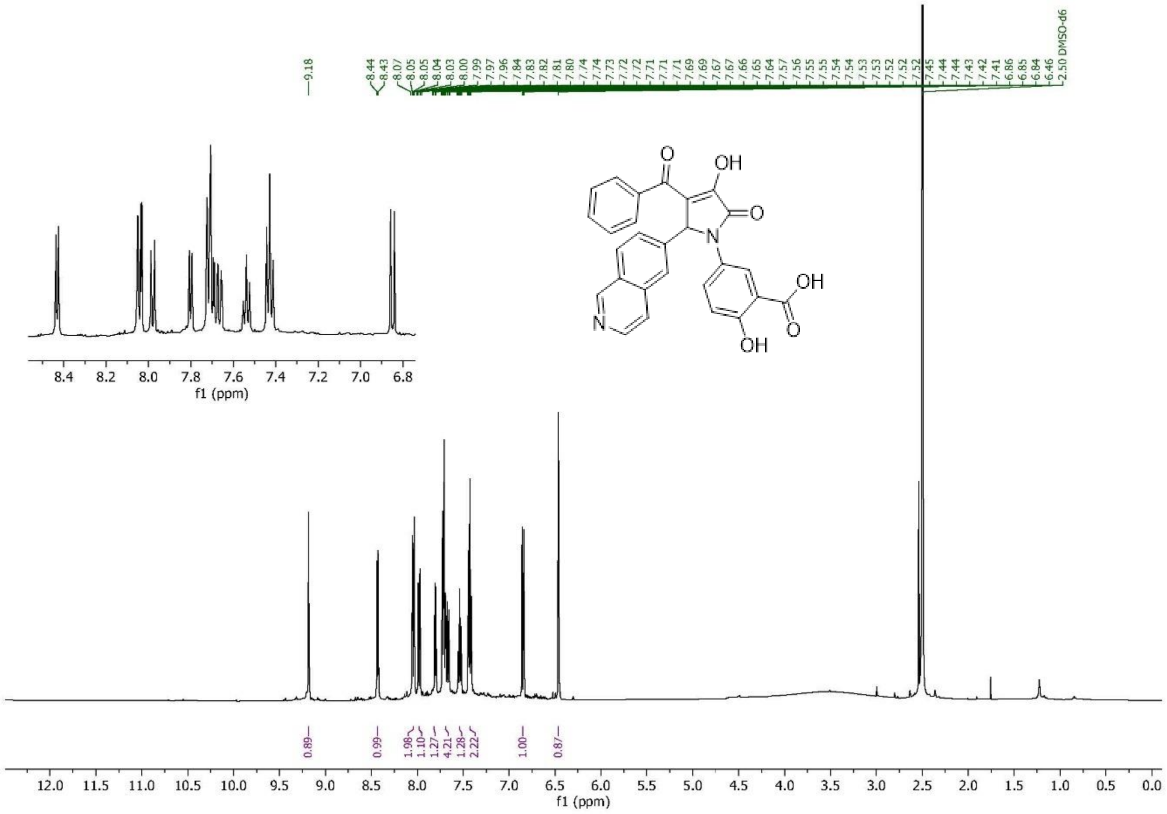

^{13}C NMR of 9 (126 MHz, DMSO-d_6):

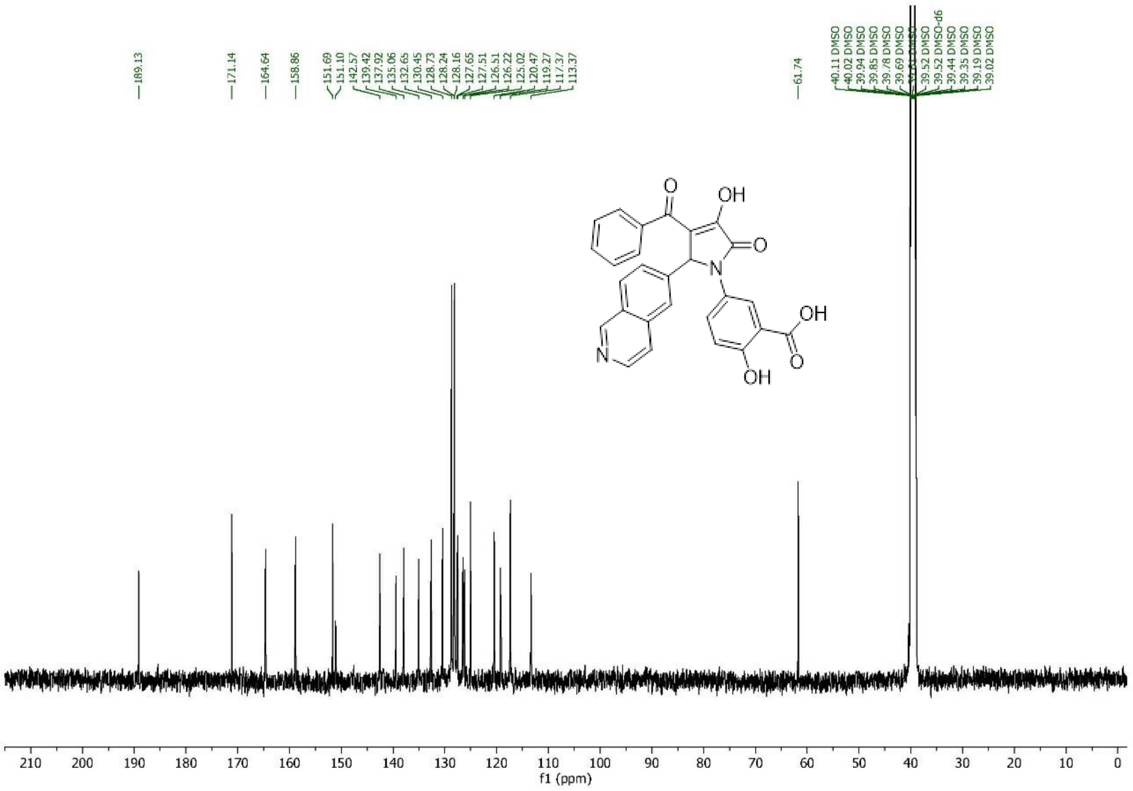

^{1}H NMR of 11 (700 MHz, DMSO-d_6):

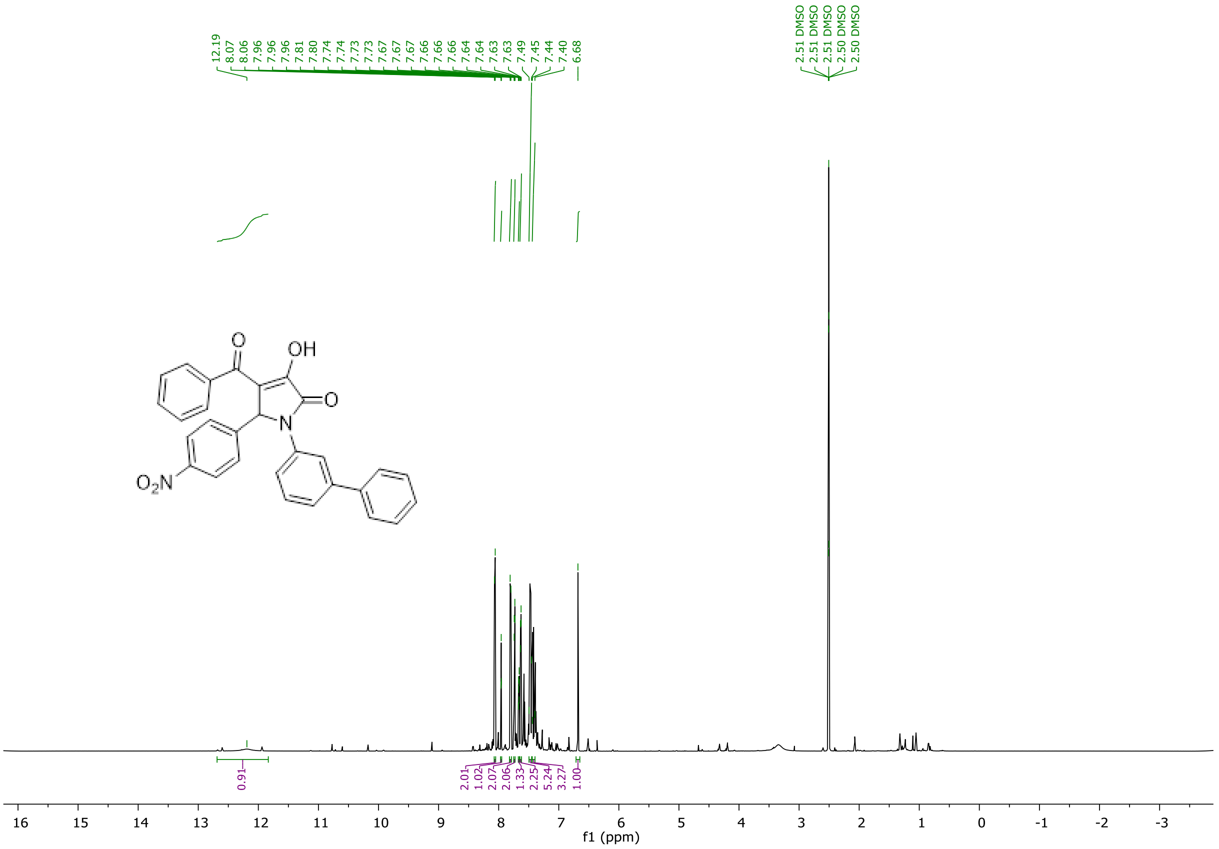

^{13}C NMR of 11 (174 MHz, DMSO-d_6):

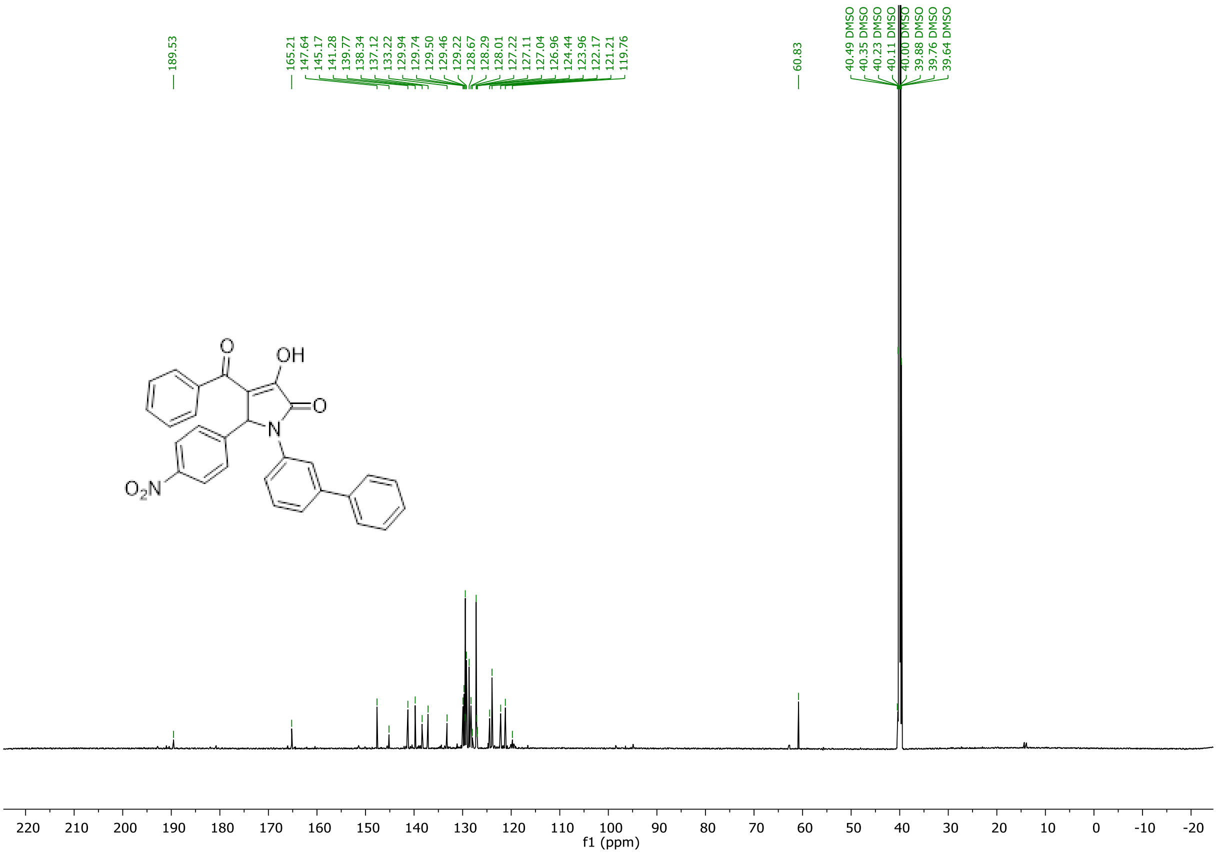

^{1}H NMR of 12 (500 MHz, DMSO-d_6):

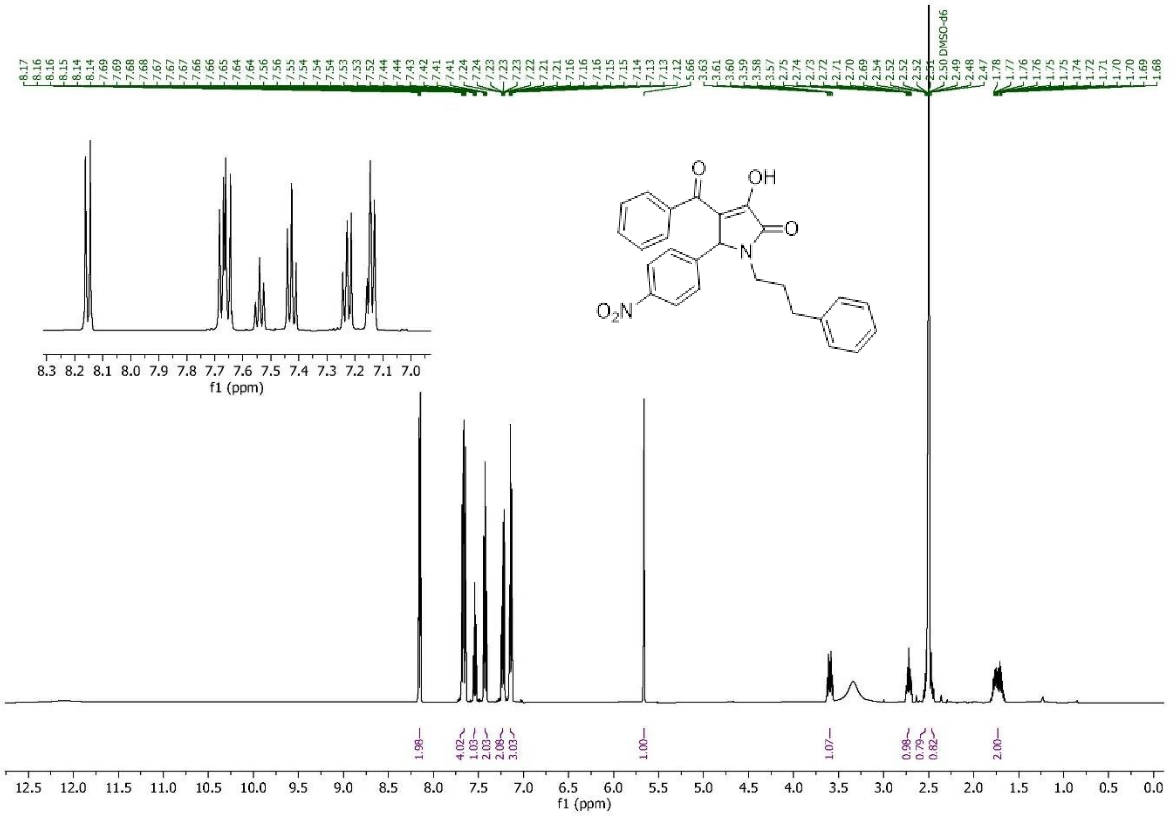

^{13}C NMR of 12 (126 MHz, DMSO-d_6):

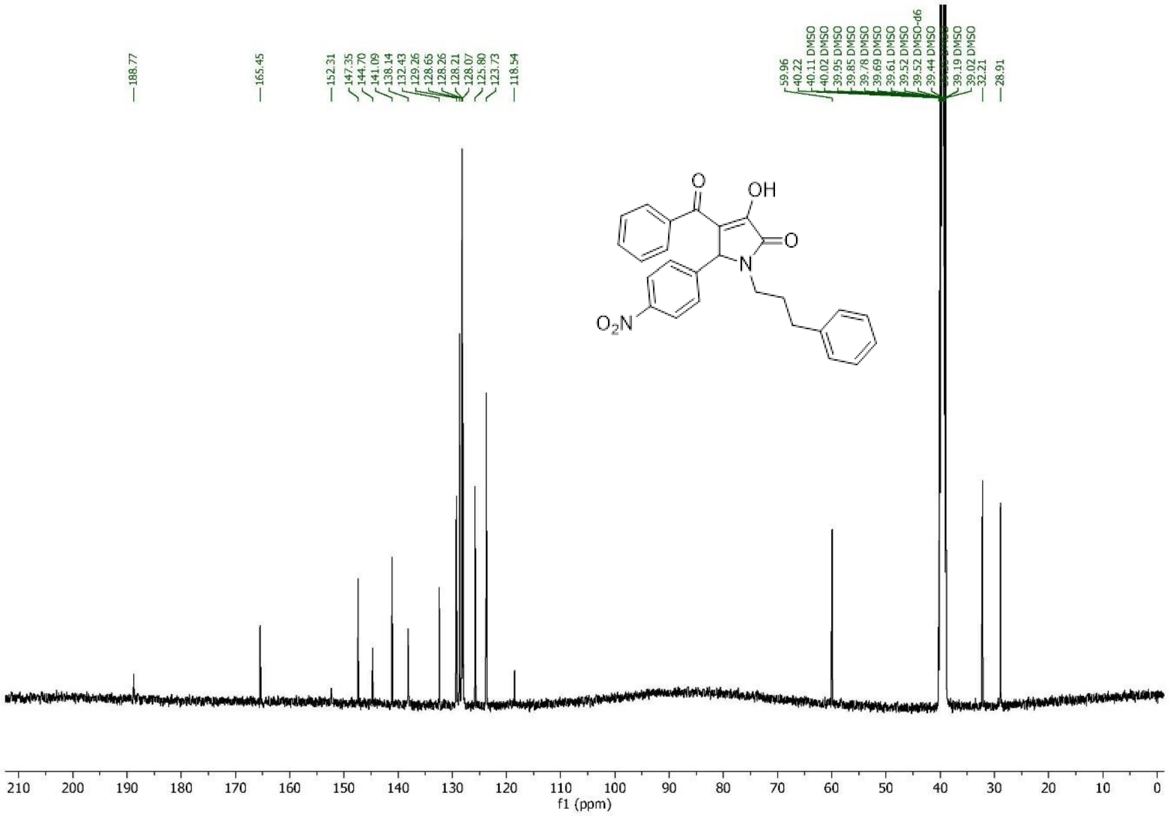

^{1}H NMR of 26 (600 MHz, DMSO-d_6):

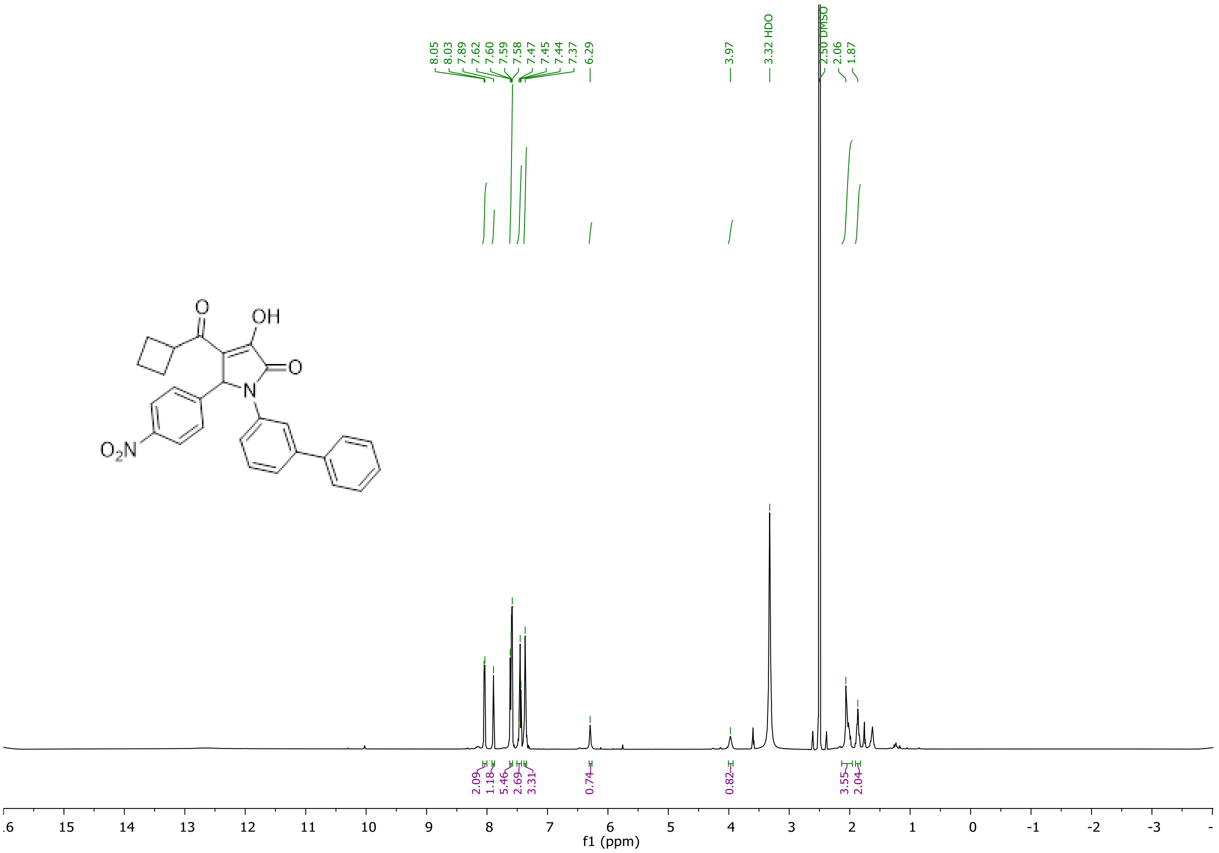

^{13}C NMR of 26 (151 MHz, DMSO-d_6):

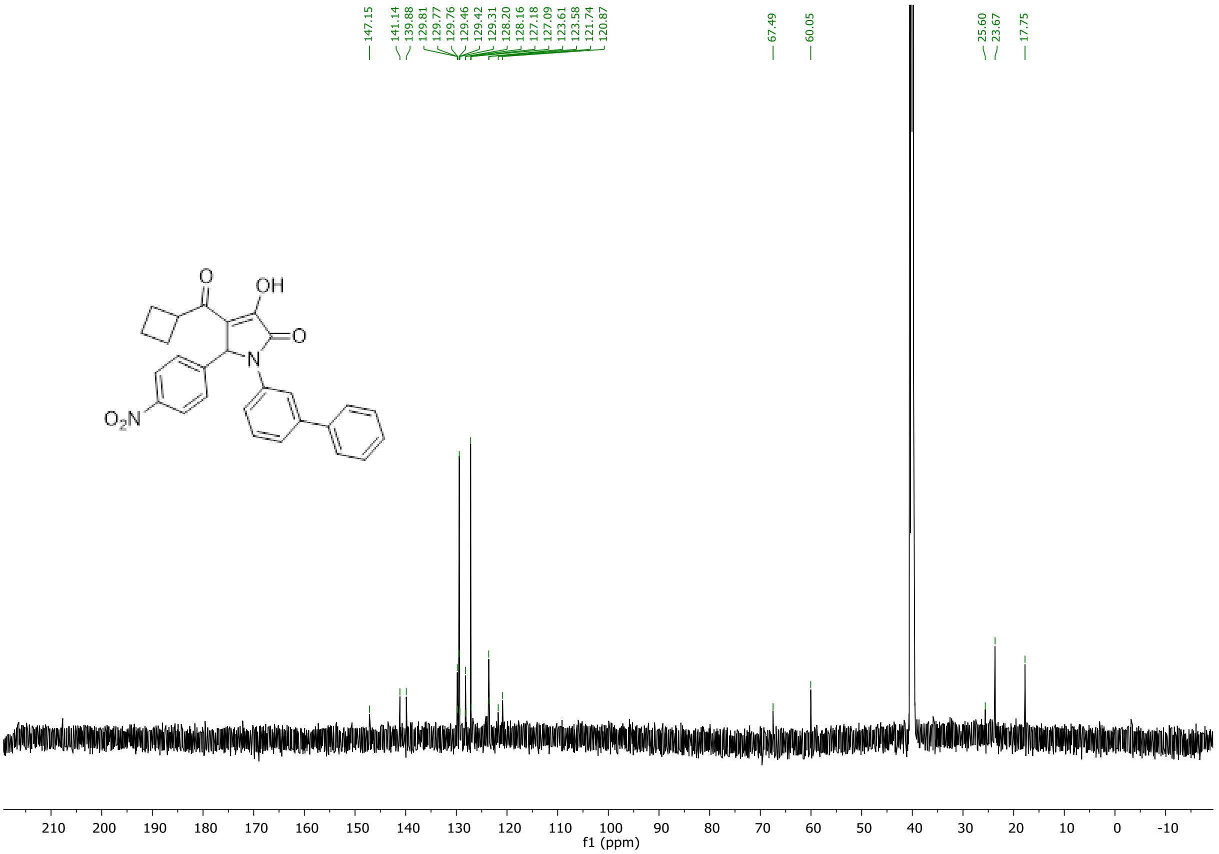

^{1}H NMR of 27 (600 MHz, DMSO-d_6), 16 ppm to -4 ppm:

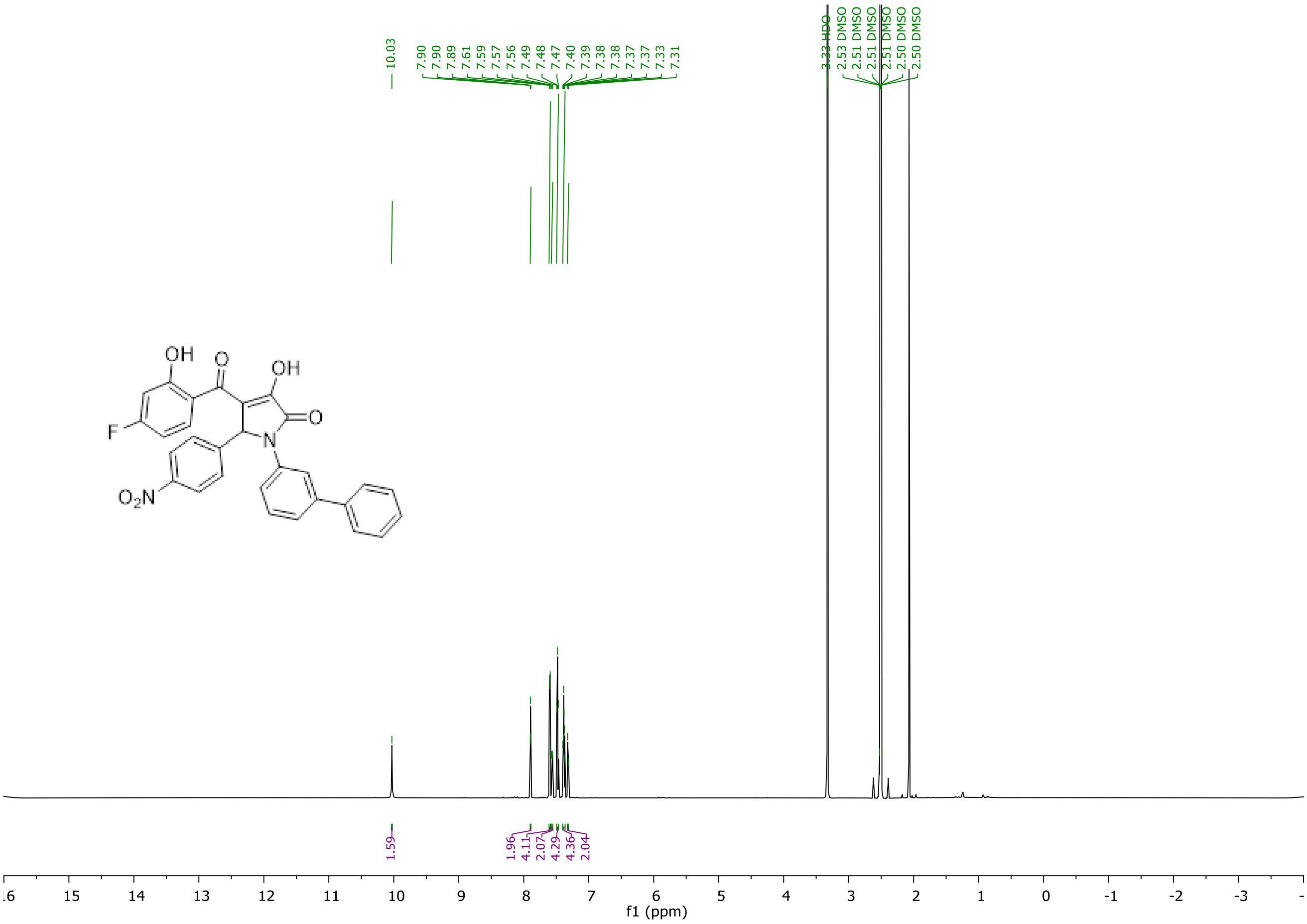

^{1}H NMR of 30 (600 MHz, DMSO-d_6):

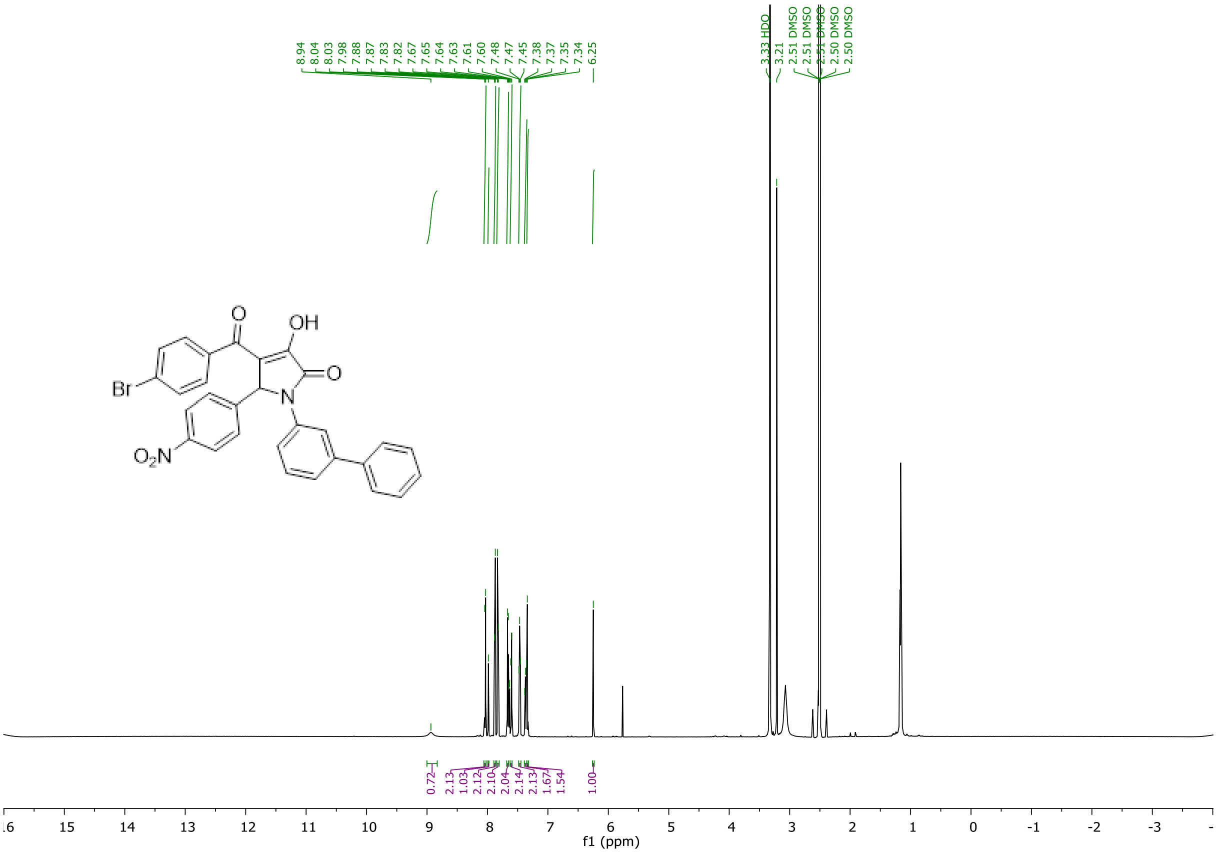

^{13}C NMR of 30 (151 MHz, DMSO-d_6):

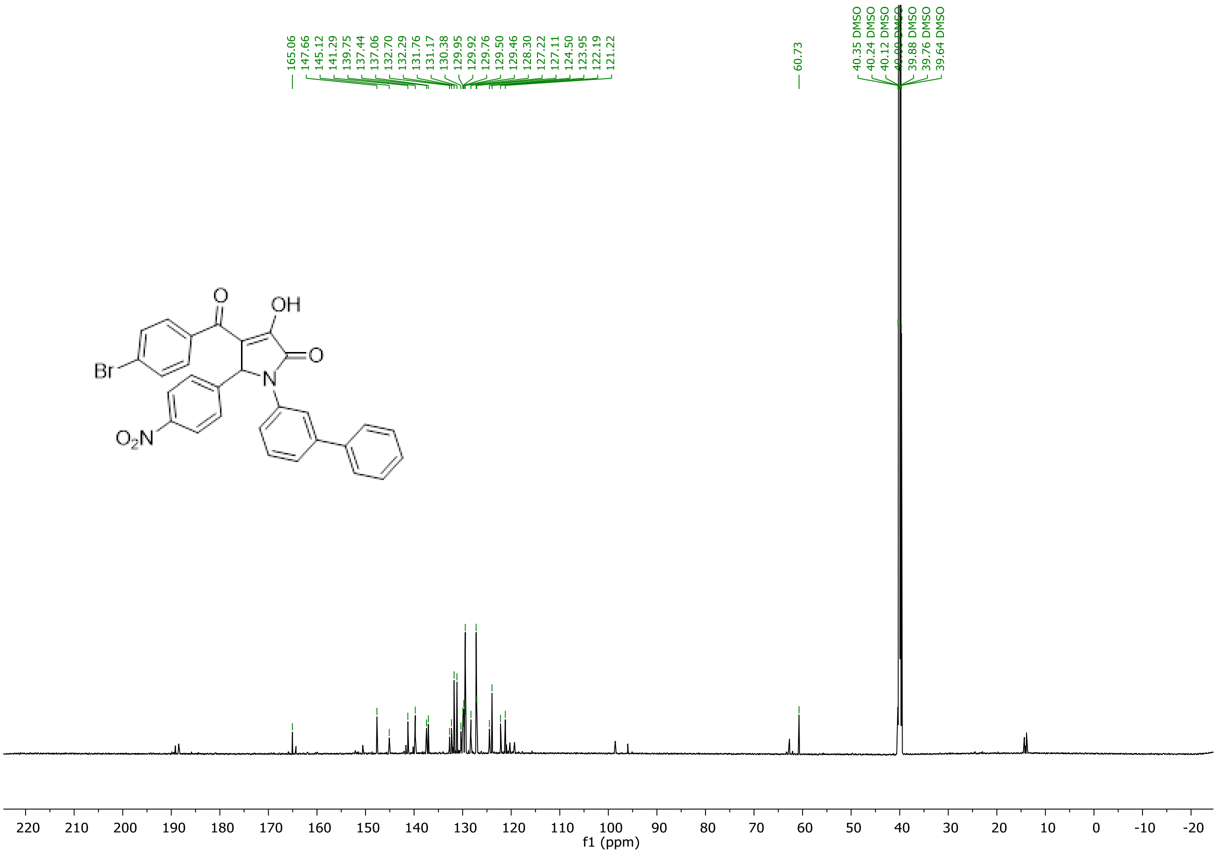

^{1}H NMR of 31 (700 MHz, DMSO-d_6):

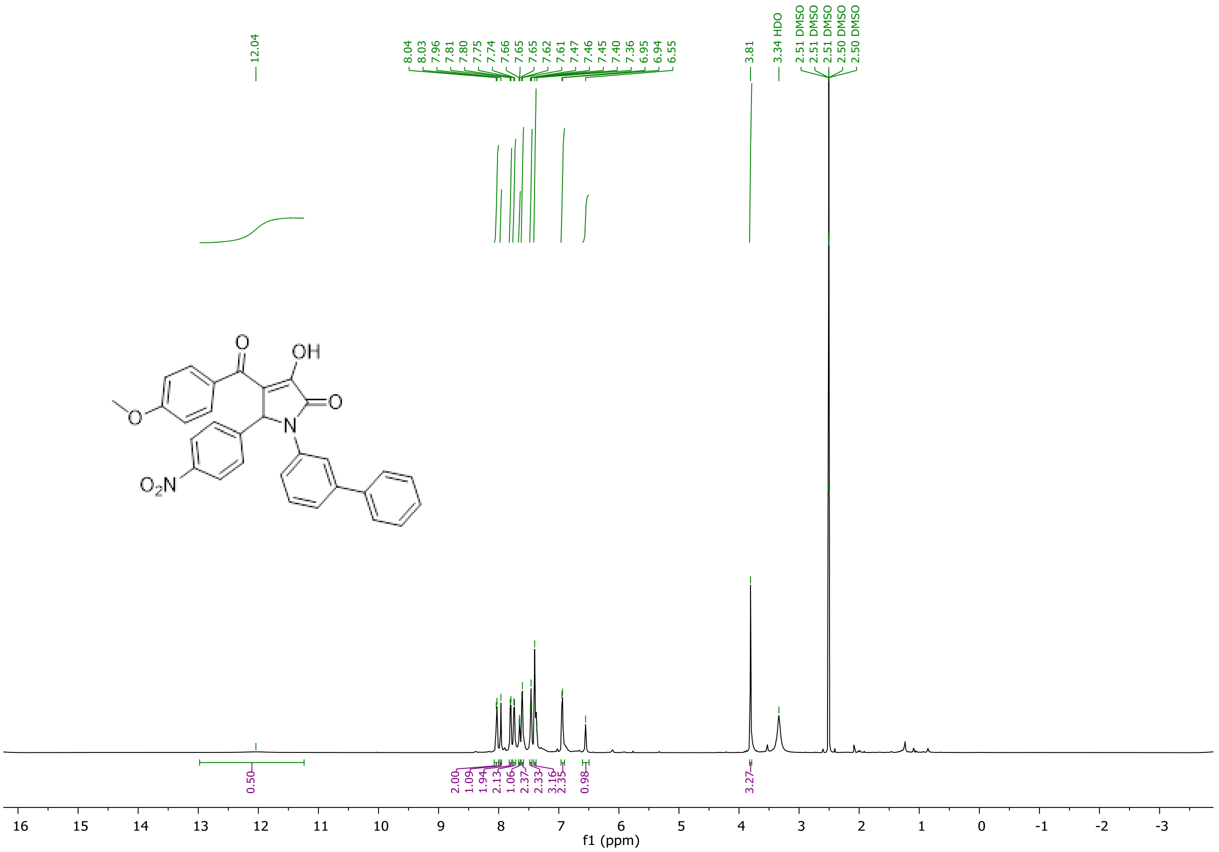

^{13}C NMR of 31 (176 MHz, DMSO-d_6):

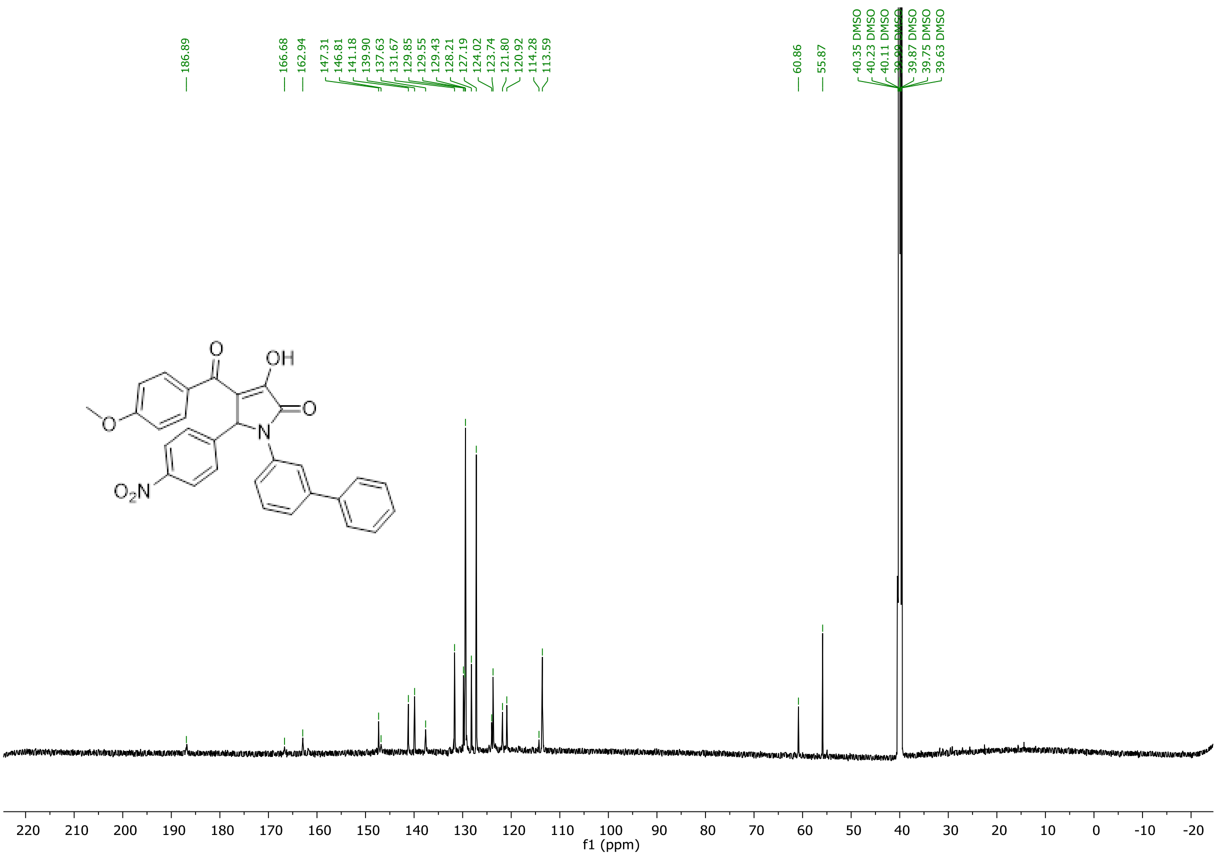

^{1}H NMR of 32 (700 MHz, DMSO-d_6):

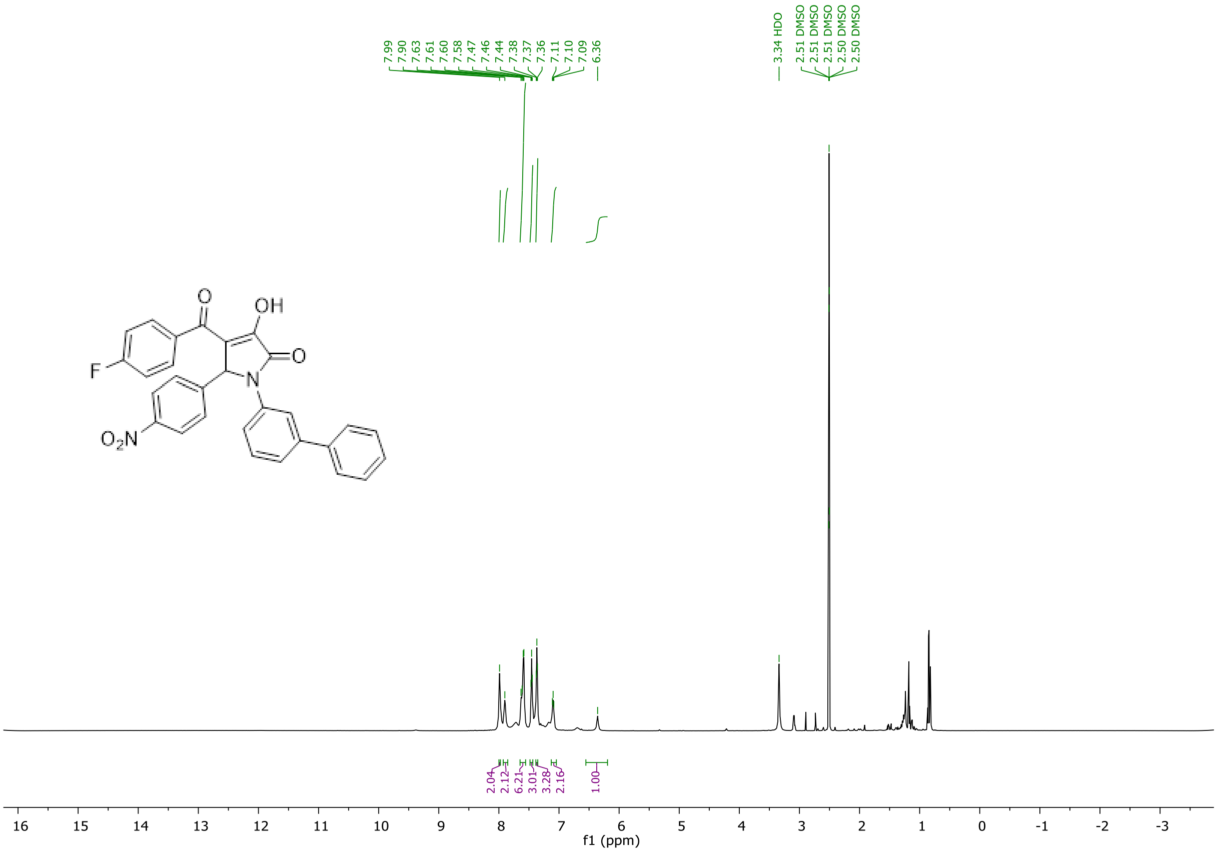

^{13} C NMR of 32 (176 MHz, DMSO-d_6):

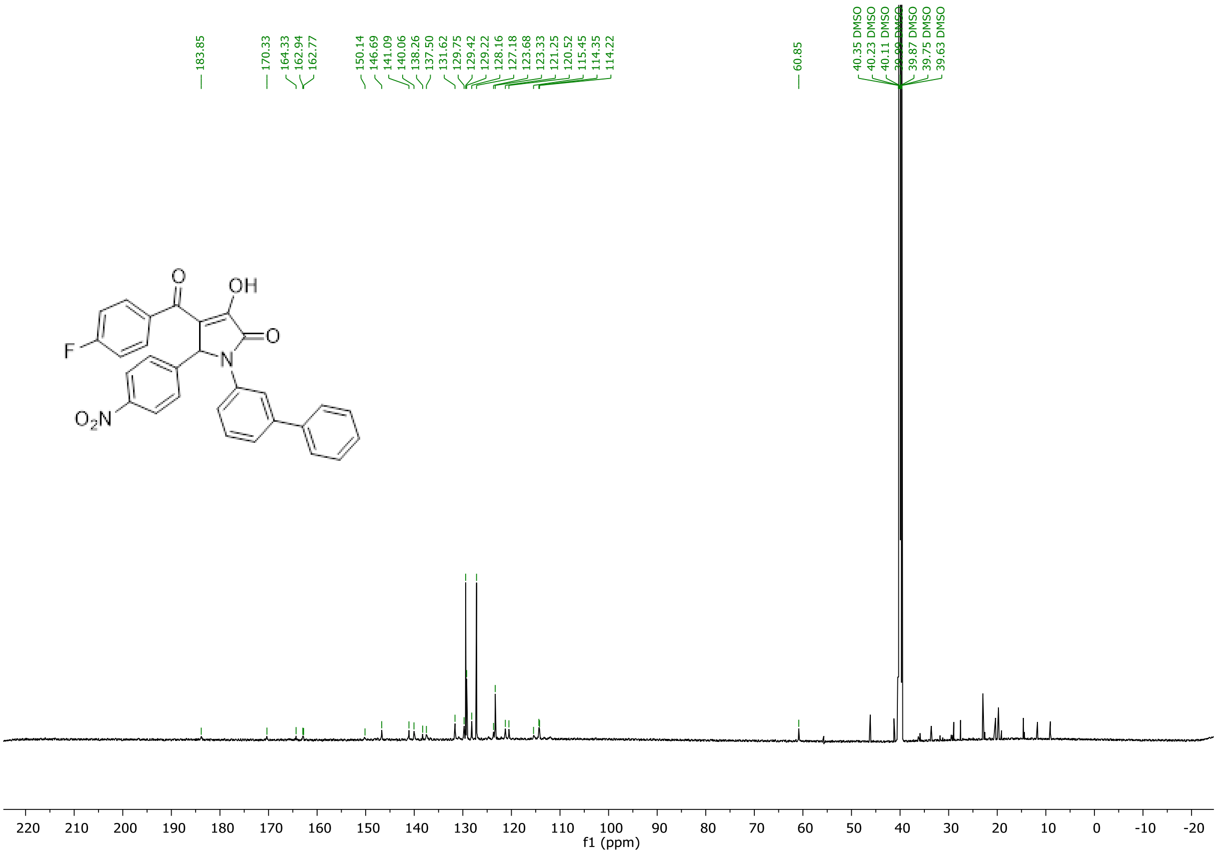

^{1}H NMR of 33 (700 MHz, DMSO-d_6):

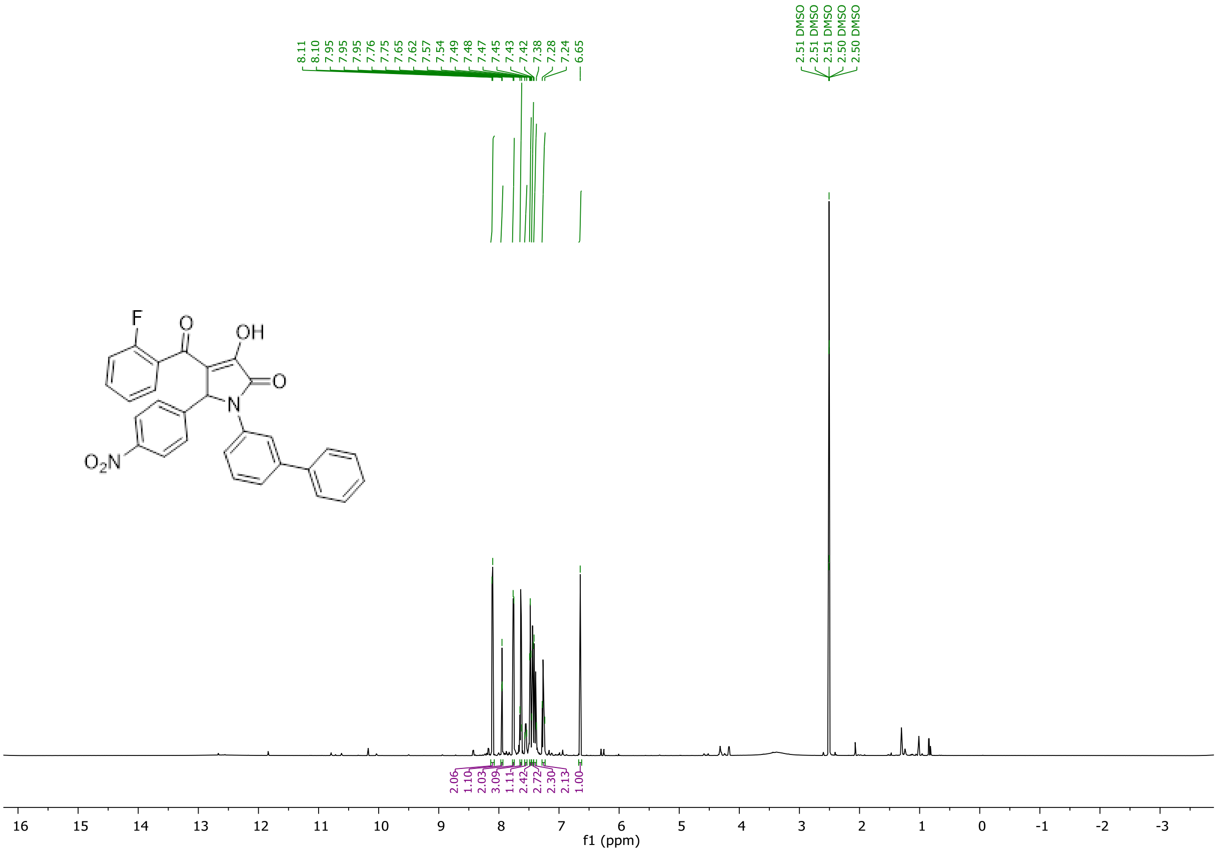

^{13}C NMR of 33 (174 MHz, DMSO-d_6):

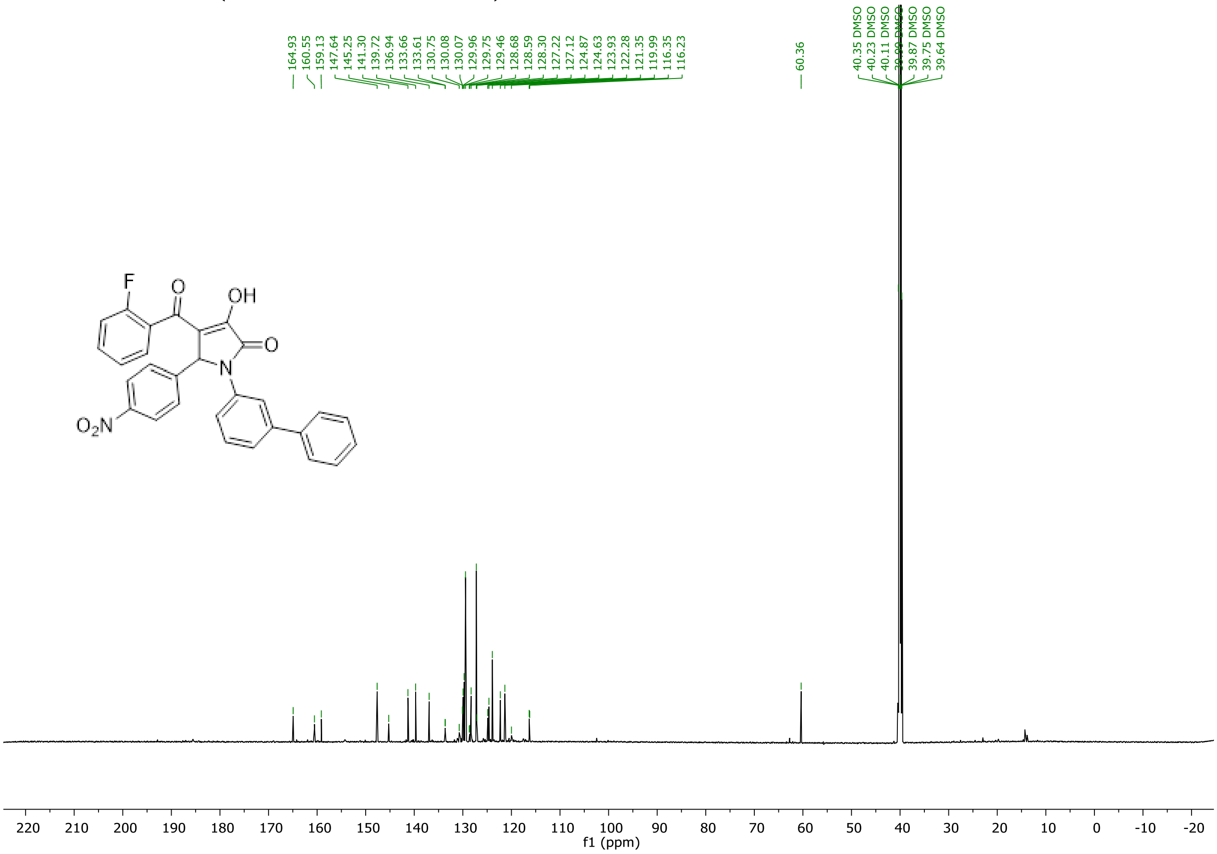

^{1}H NMR of 35 (700 MHz, DMSO-d_6):

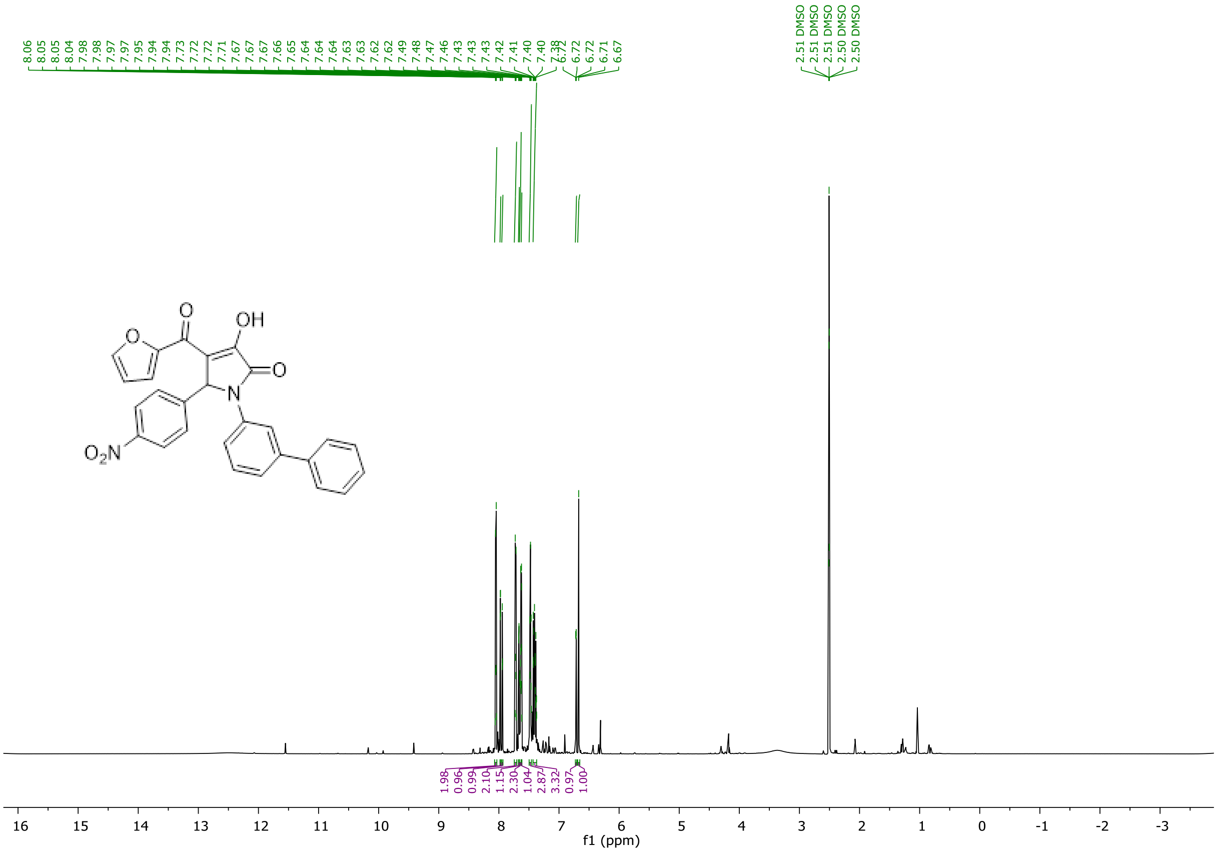

^{13}C NMR of 35 (176 MHz, DMSO-d_6):

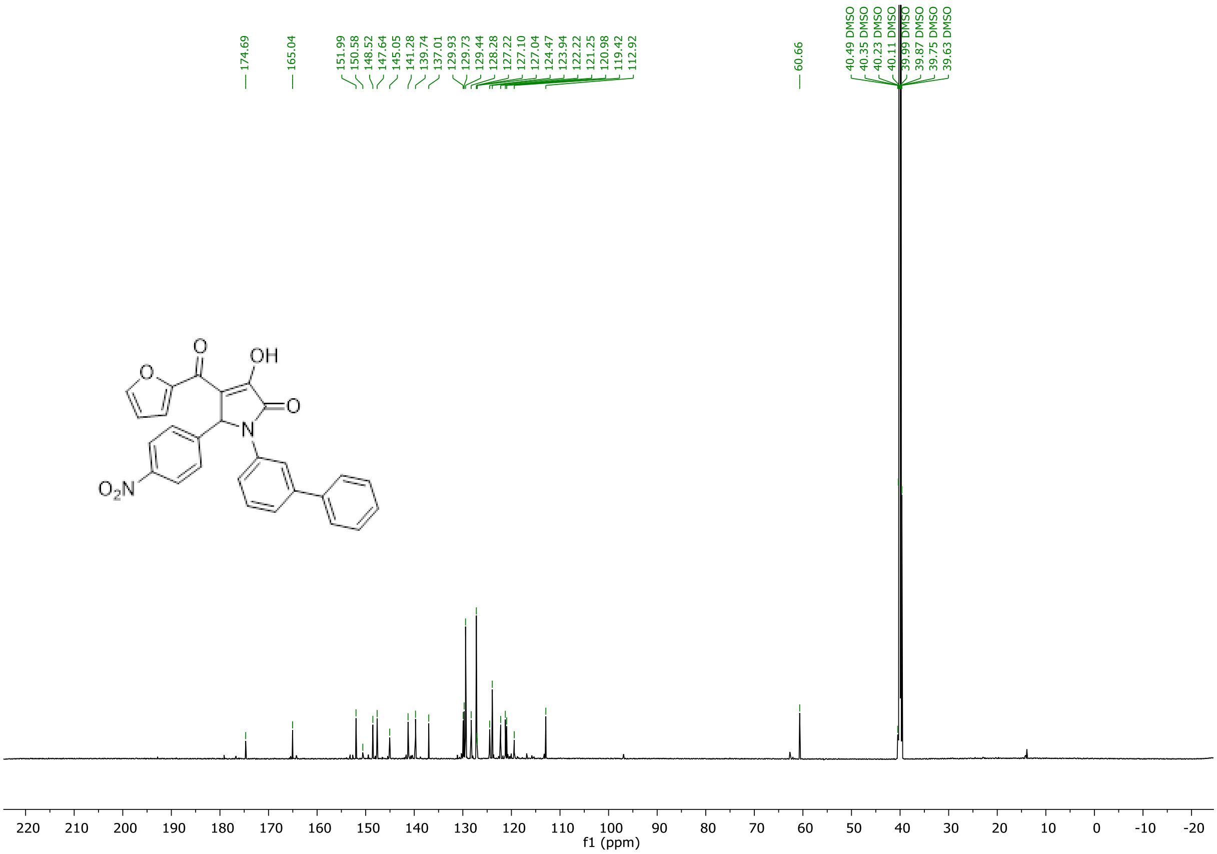

^{1}H NMR of 36 (700 MHz, DMSO-d_6):

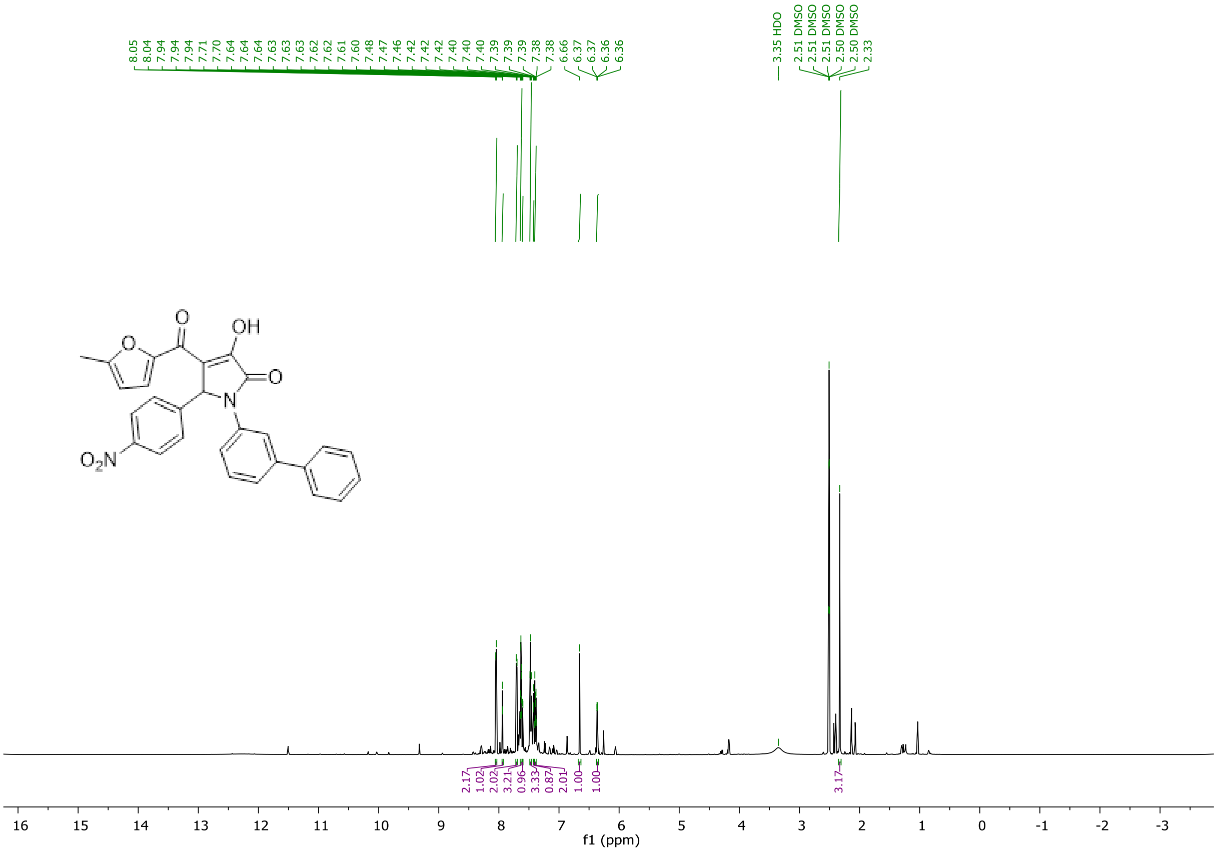

^{13}C NMR of 36 (174 MHz, DMSO-d_6):

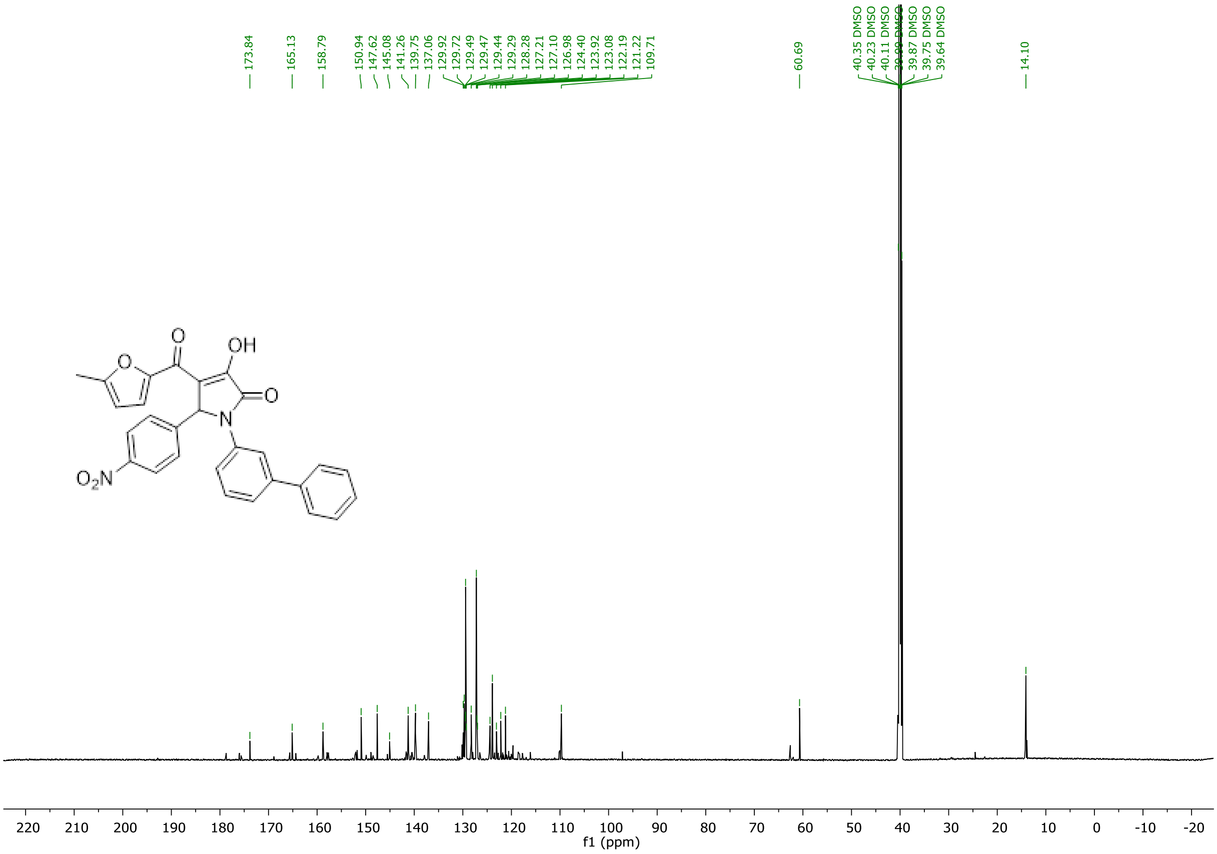

^{1}H NMR of 37 (700 MHz, DMSO-d_6):

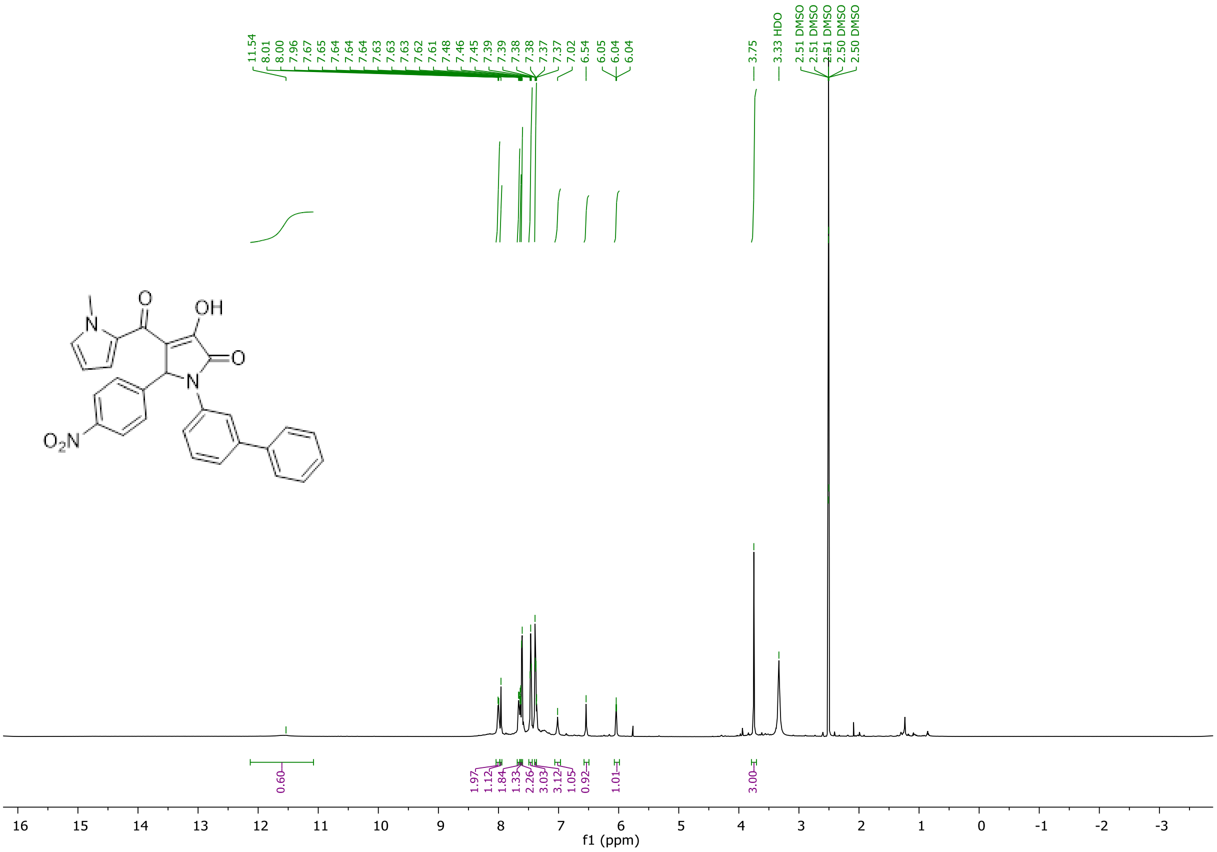

^{13} C NMR of 37 (176 MHz, DMSO-d_6):

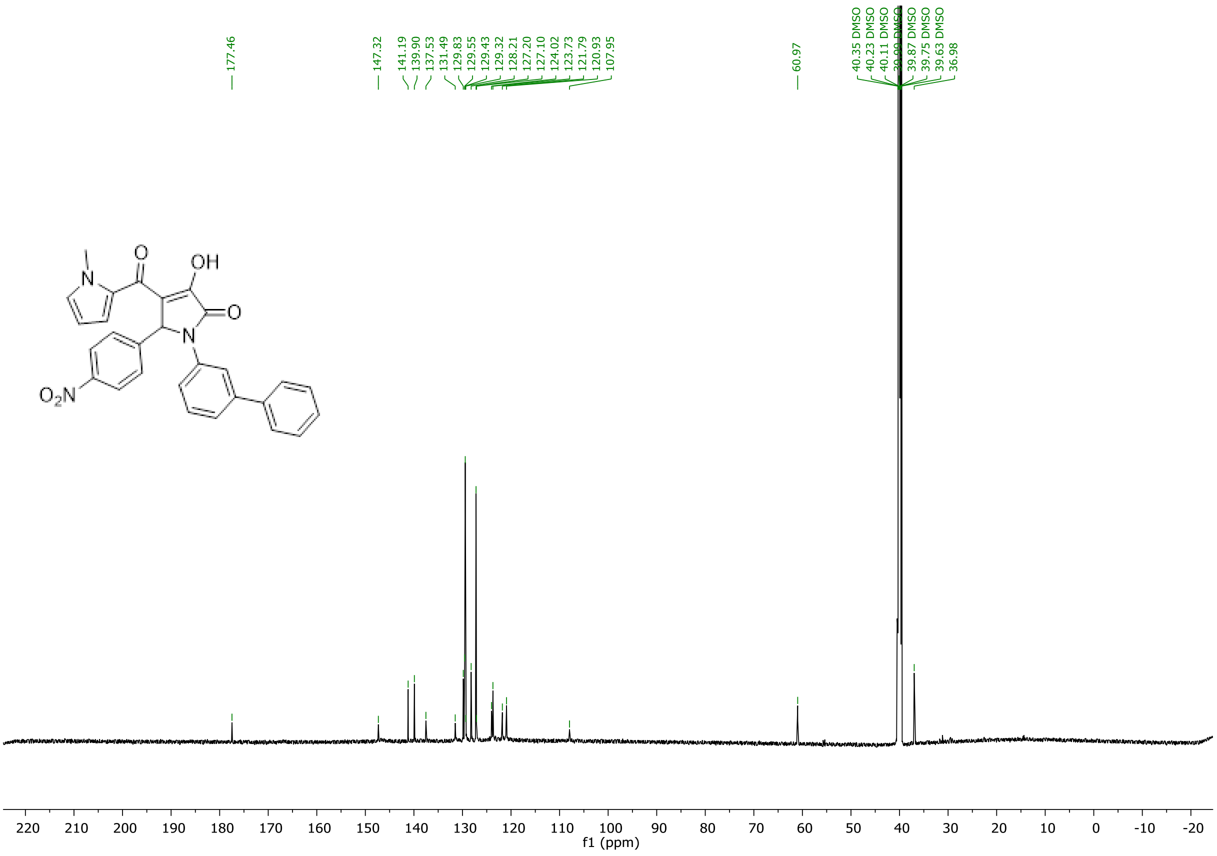

^{1}H NMR of 38 (600 MHz, DMSO-d_6):

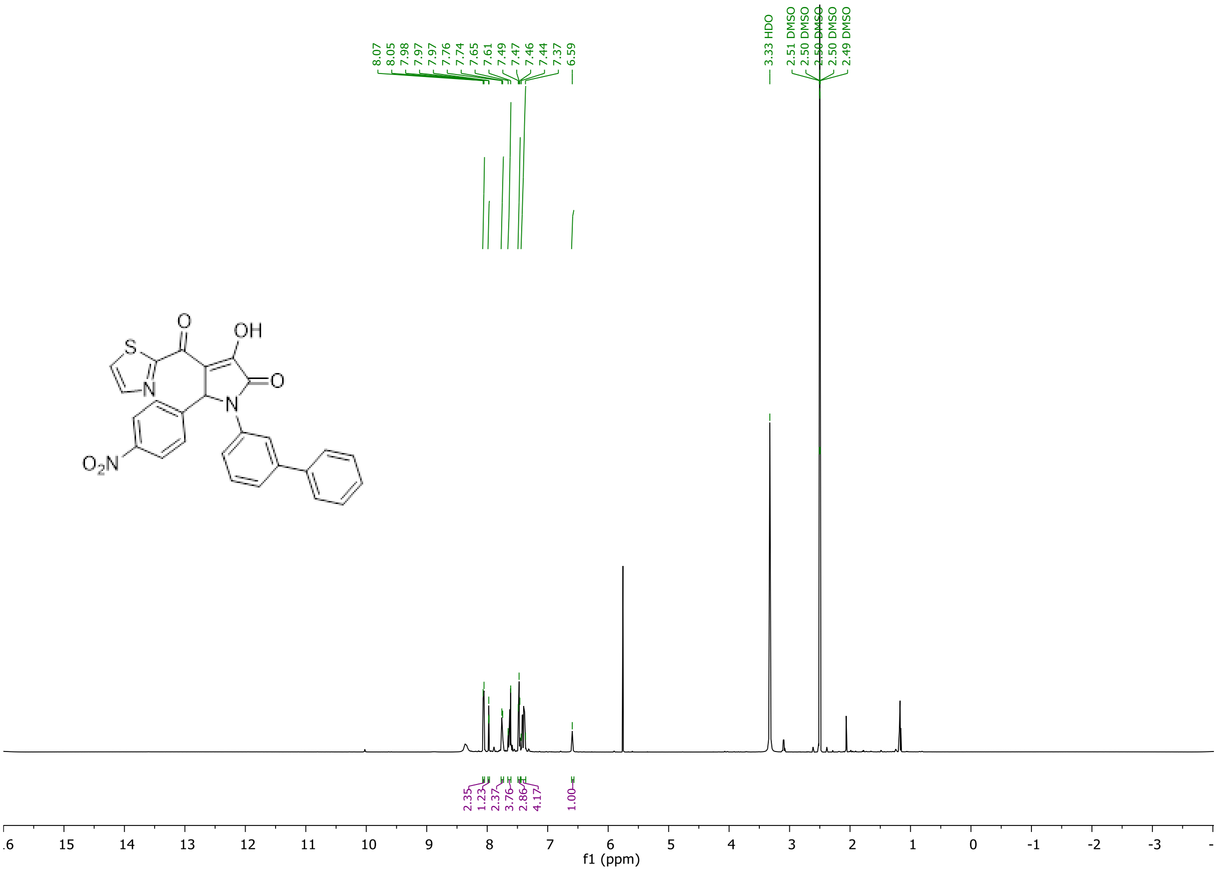

^{13}C NMR of 38 (151 MHz, DMSO-d_6):

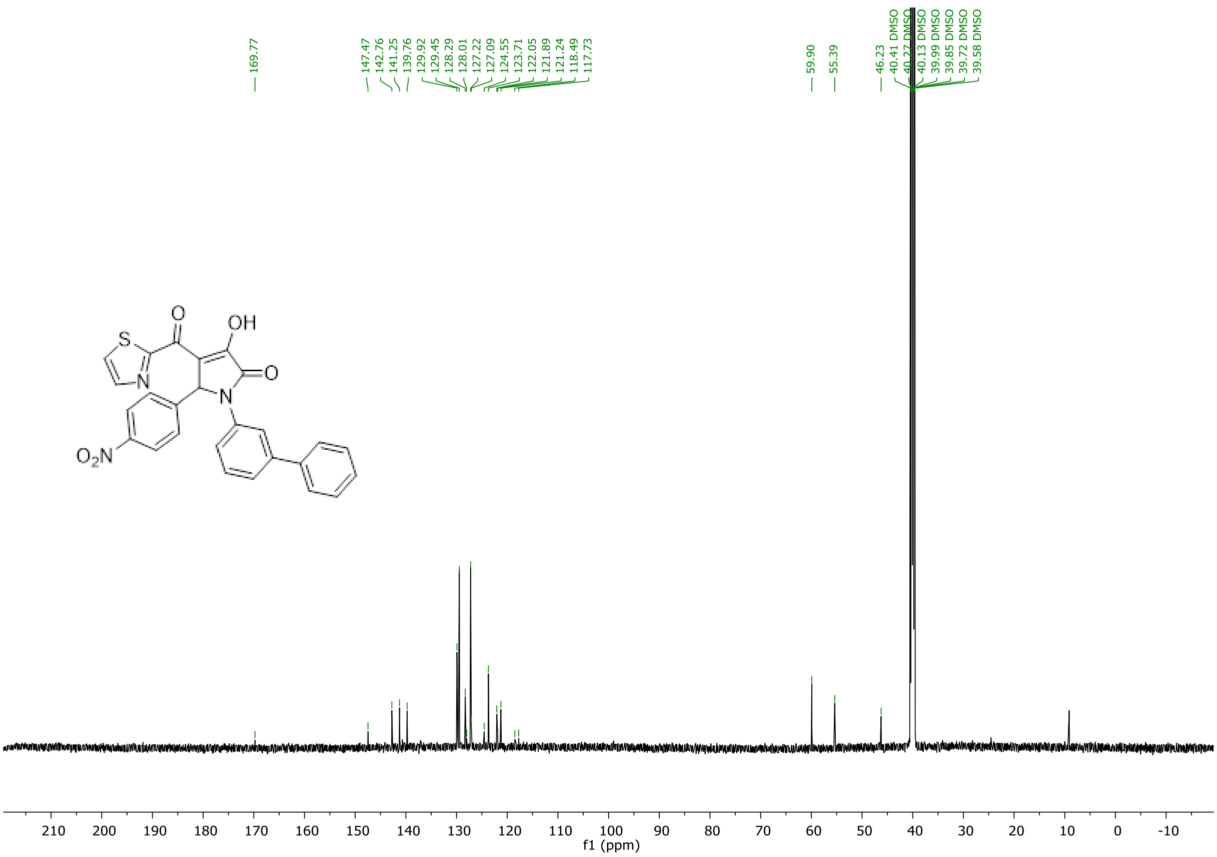

^{1}H NMR of 40 (700 MHz, DMSO-d_6):

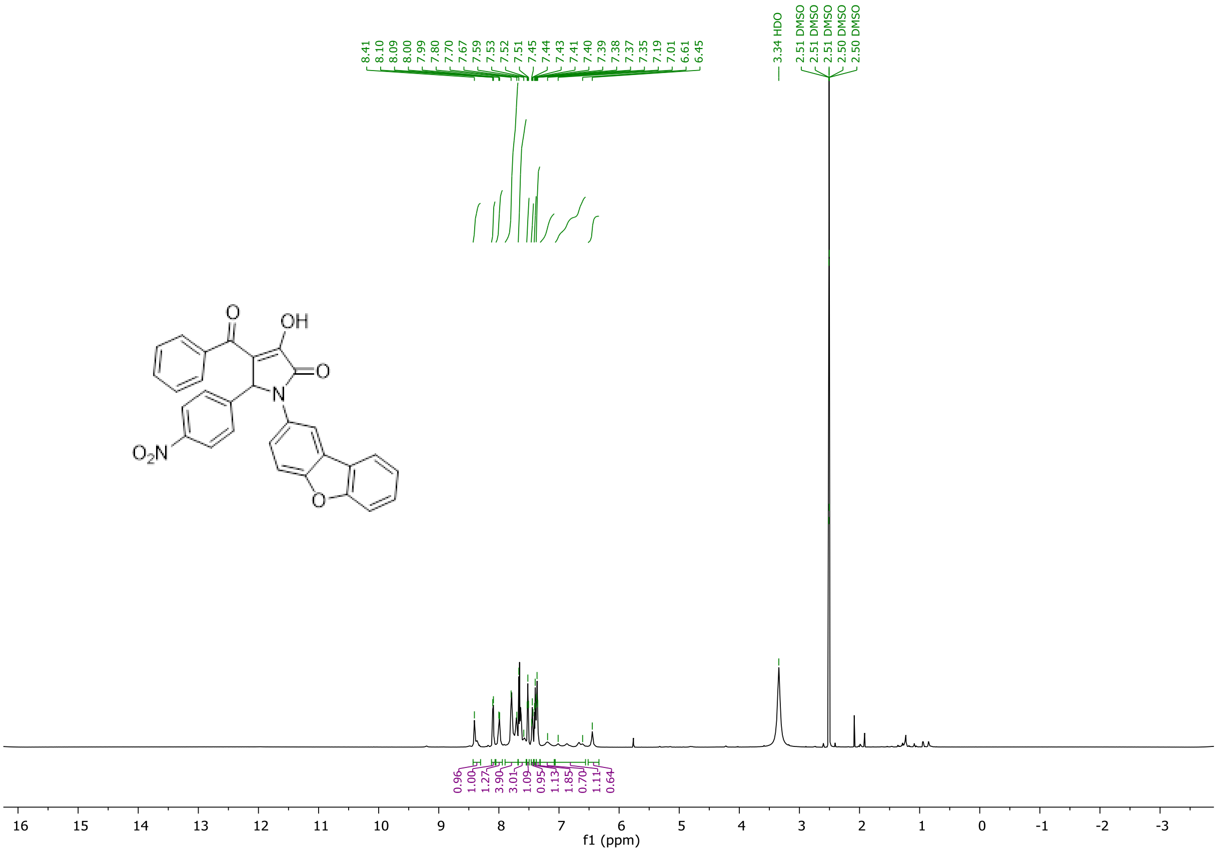

^{13}C NMR of 40 (176 MHz, DMSO-d_6):

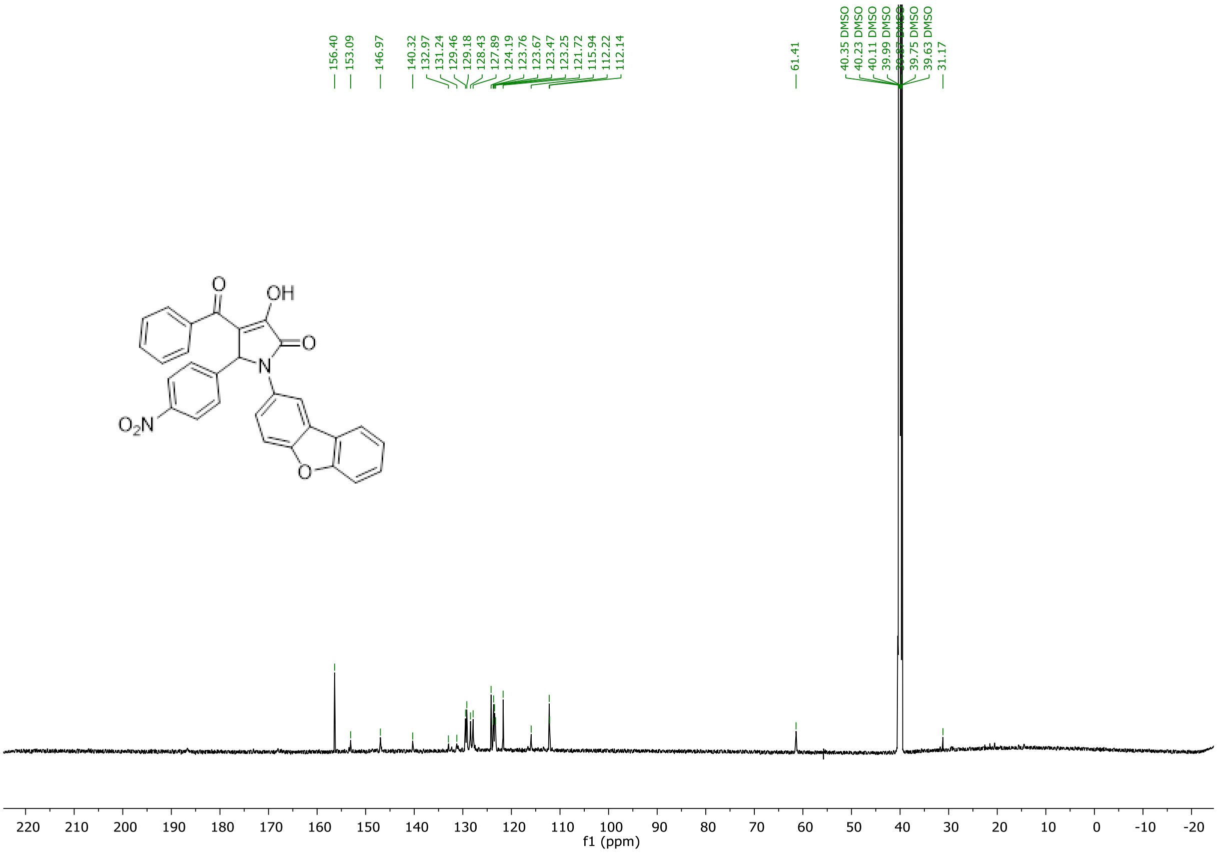

^{1}H NMR of 41 (700 MHz, DMSO-d_6):

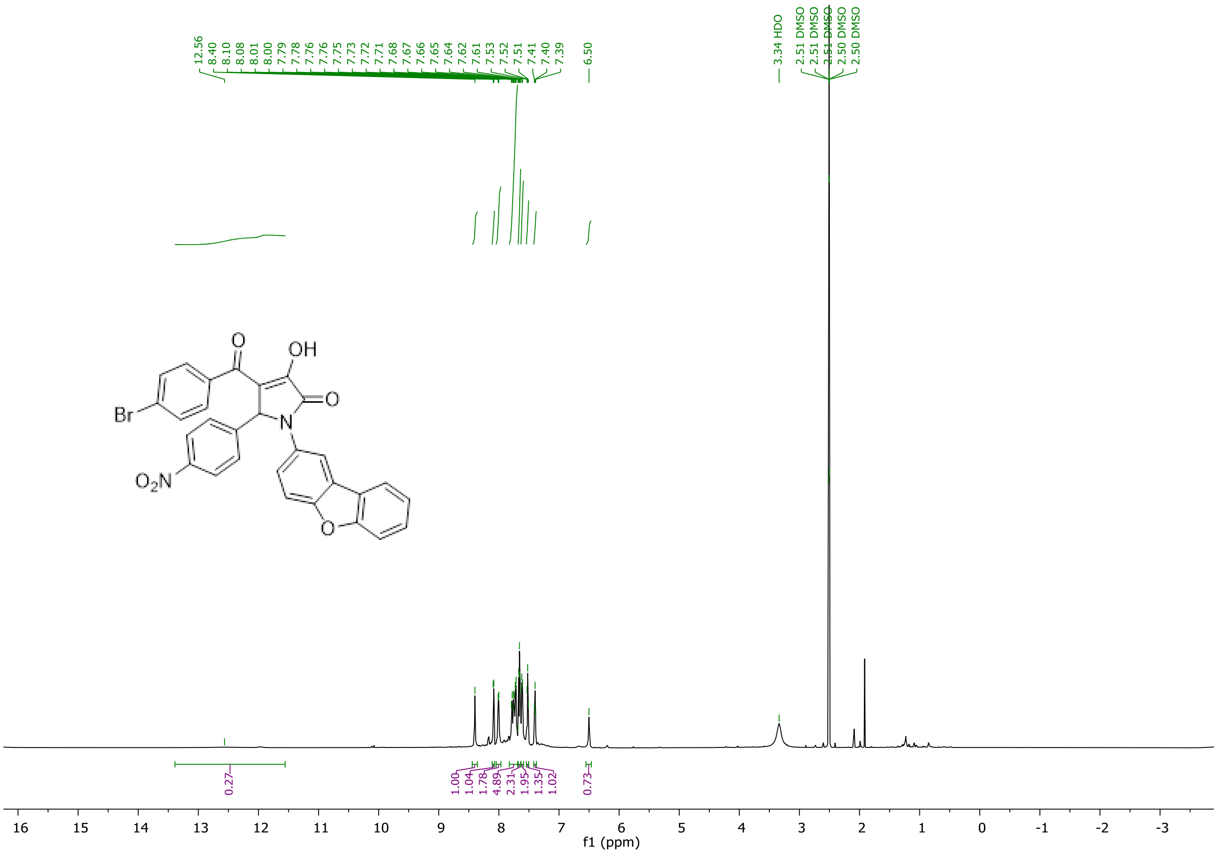

^{13}C NMR of 41 (176 MHz, DMSO-d_6):

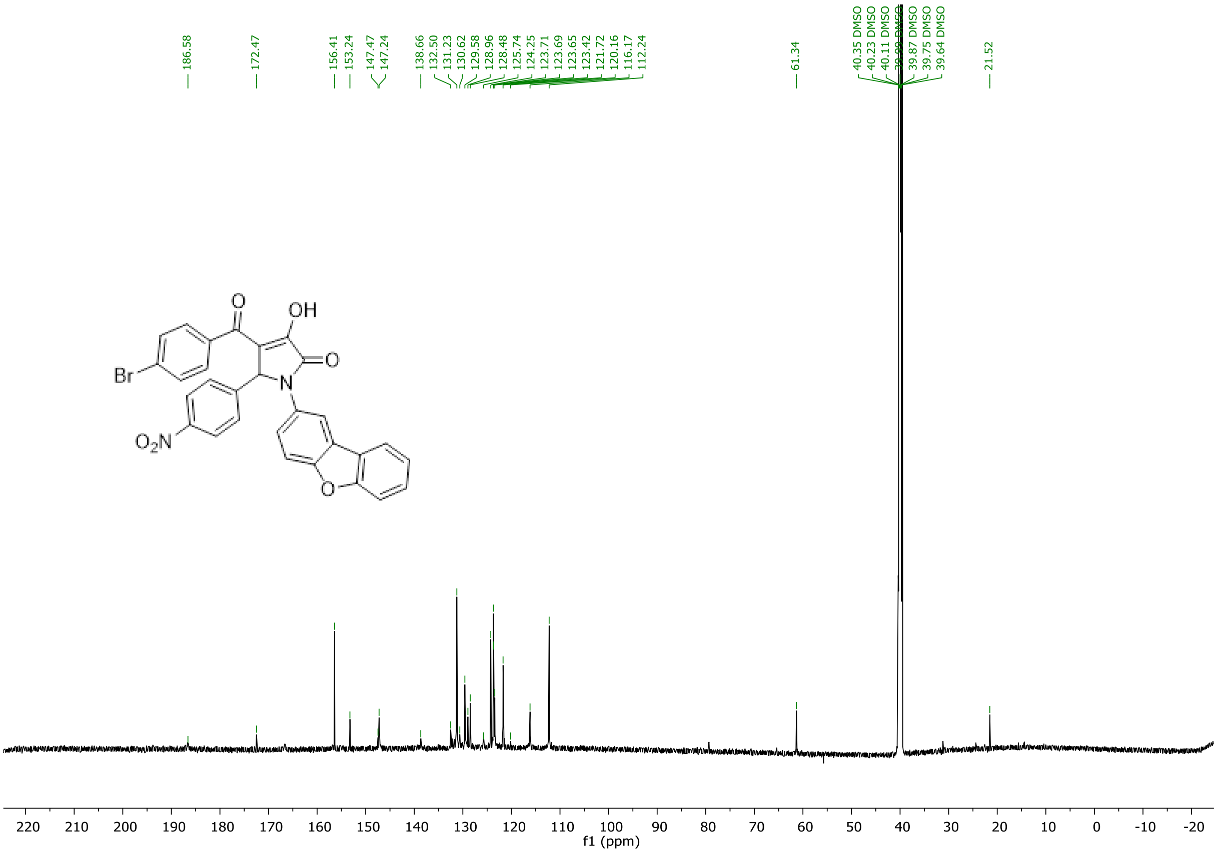

^{1}H NMR of 42 (700 MHz, DMSO-d_6):

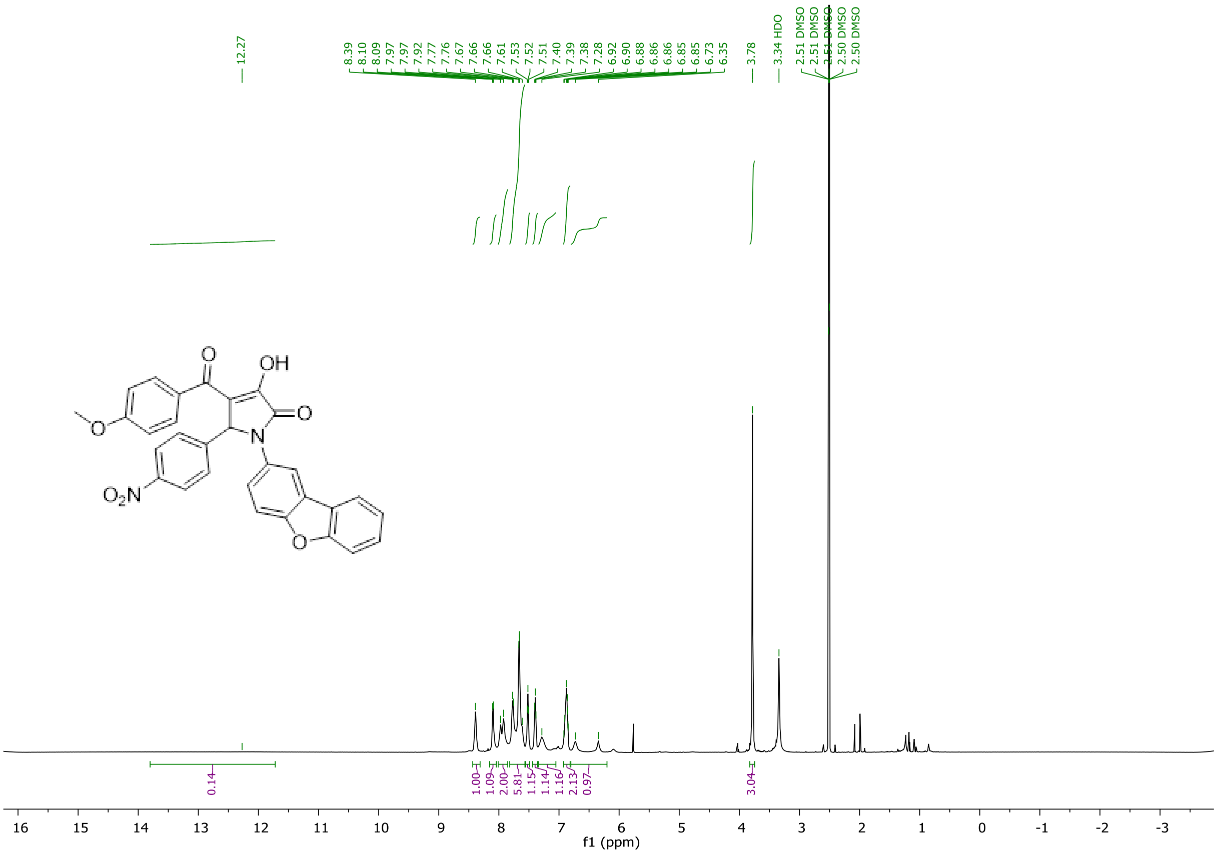

^{13}C NMR of 42 (176 MHz, DMSO-d_6):

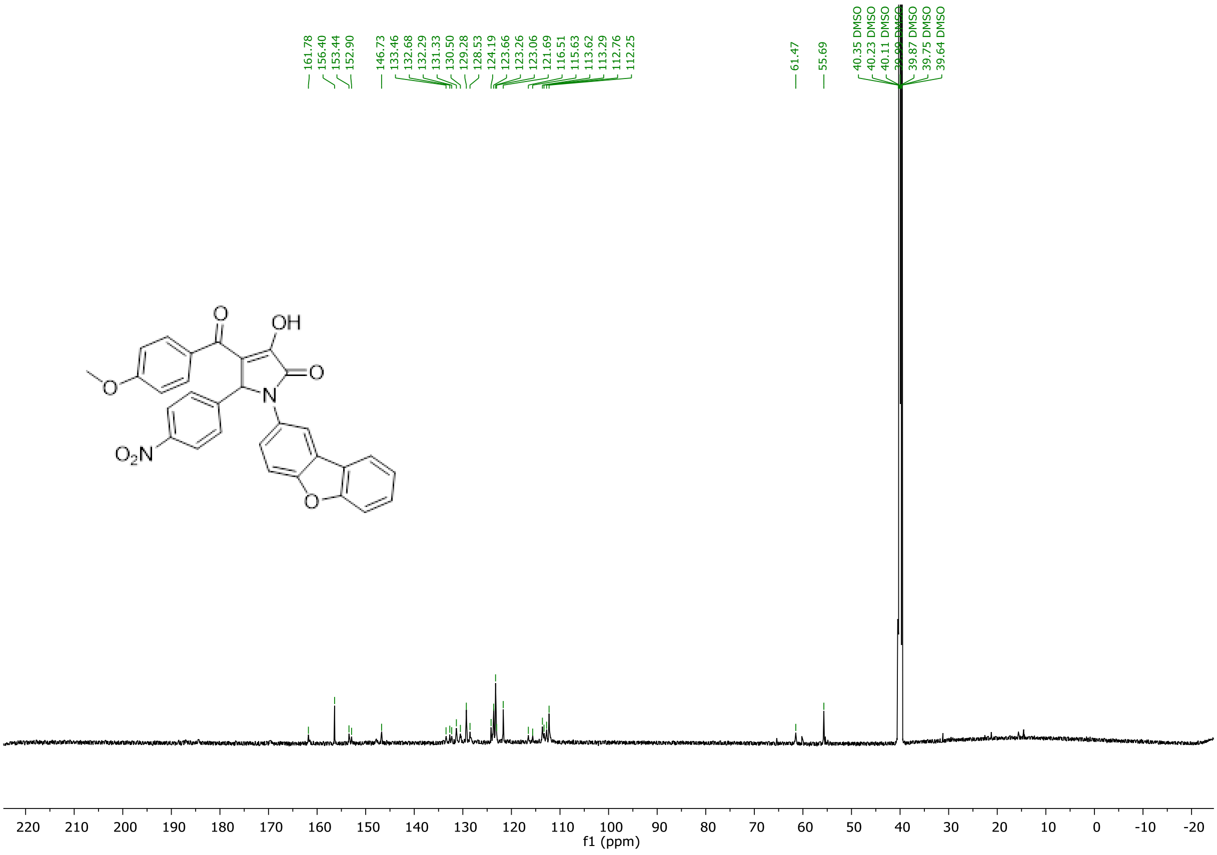

^{1}H NMR of 43 (700 MHz, DMSO-d_6):

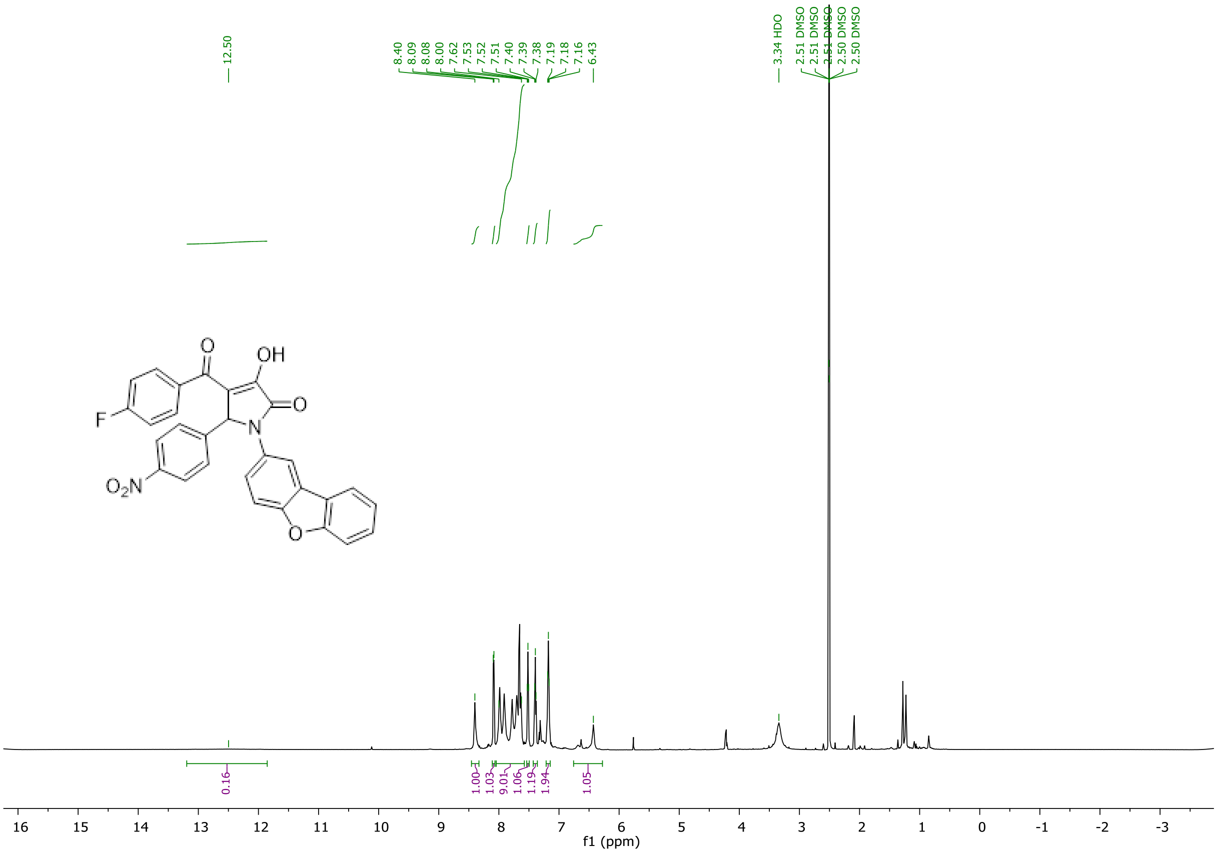

^{13}C NMR of 43 (176 MHz, DMSO-d_6):

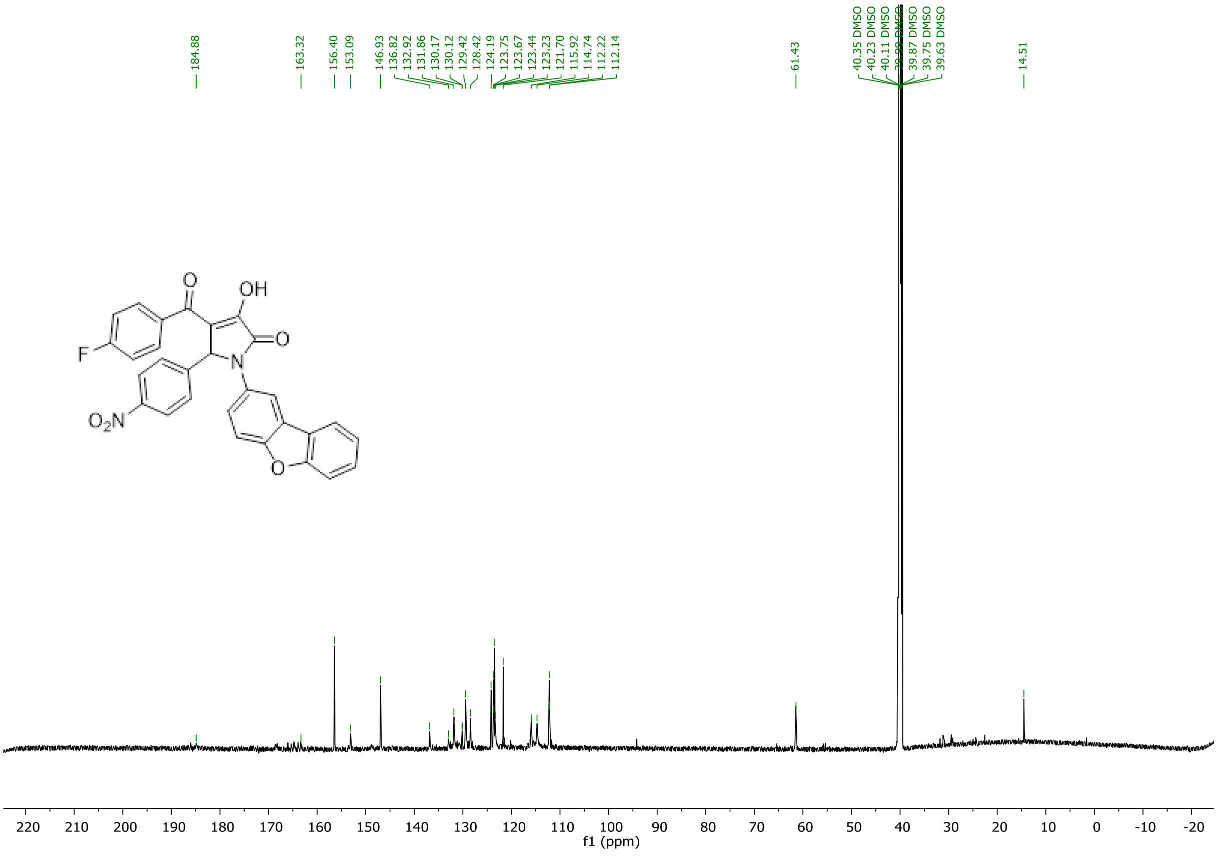

^{1}H NMR of 44 (700 MHz, DMSO-d_6):

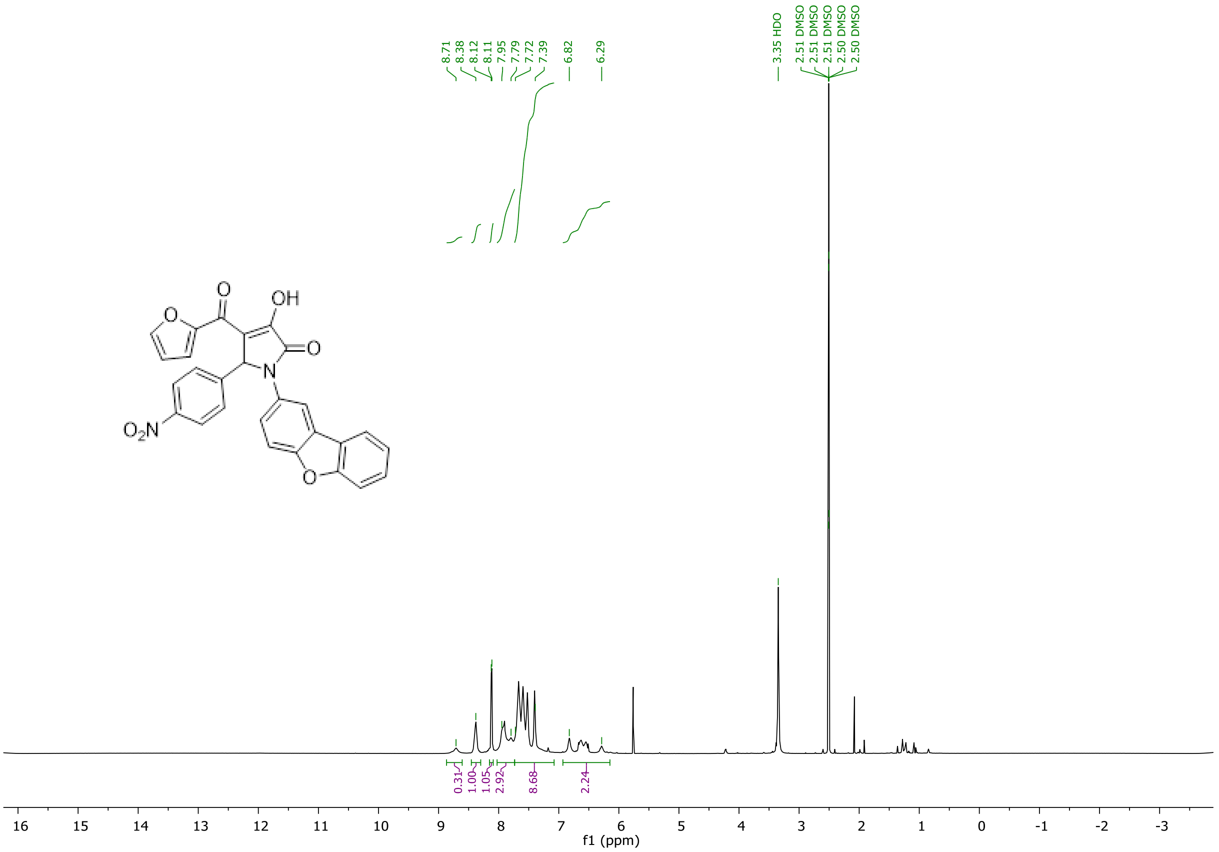

^{13}C NMR of 44 (176 MHz, DMSO-d_6):

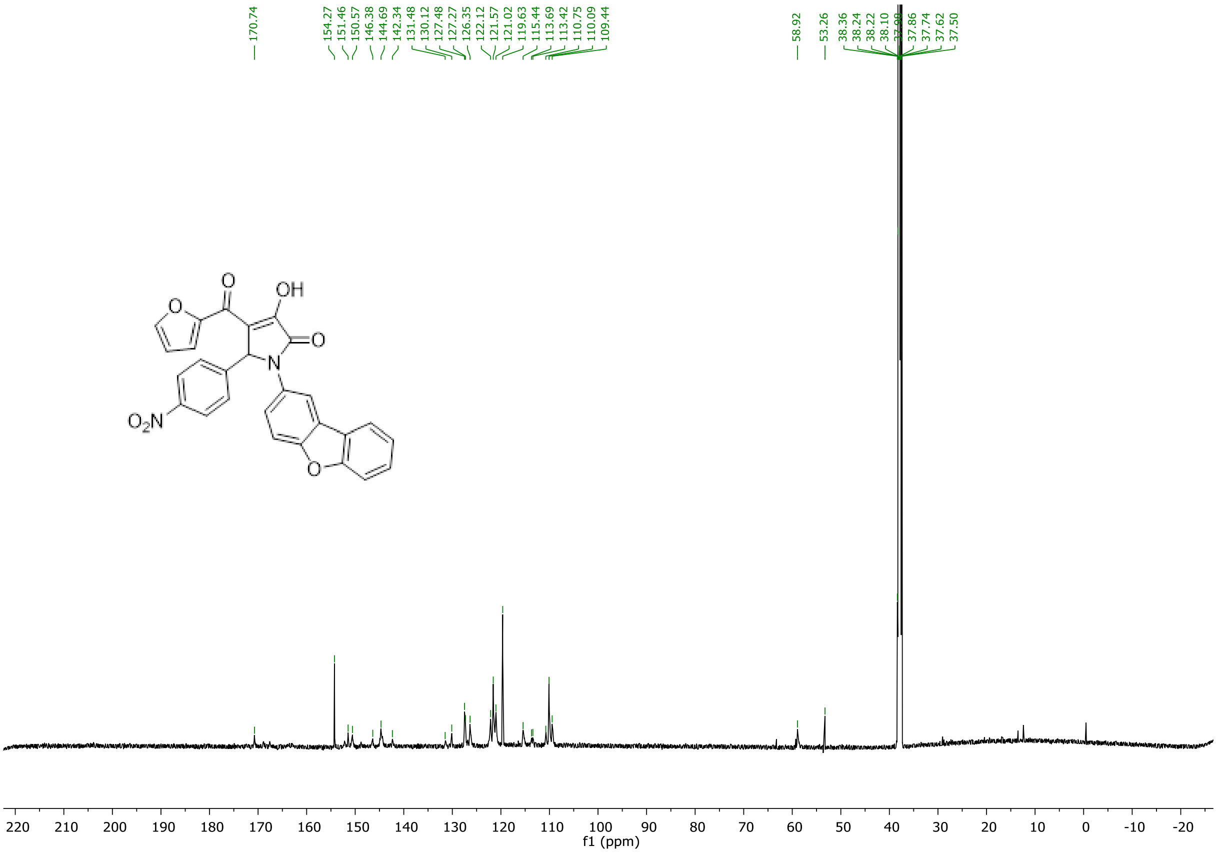

^{1}H NMR of 45 (700 MHz, DMSO-d_6):

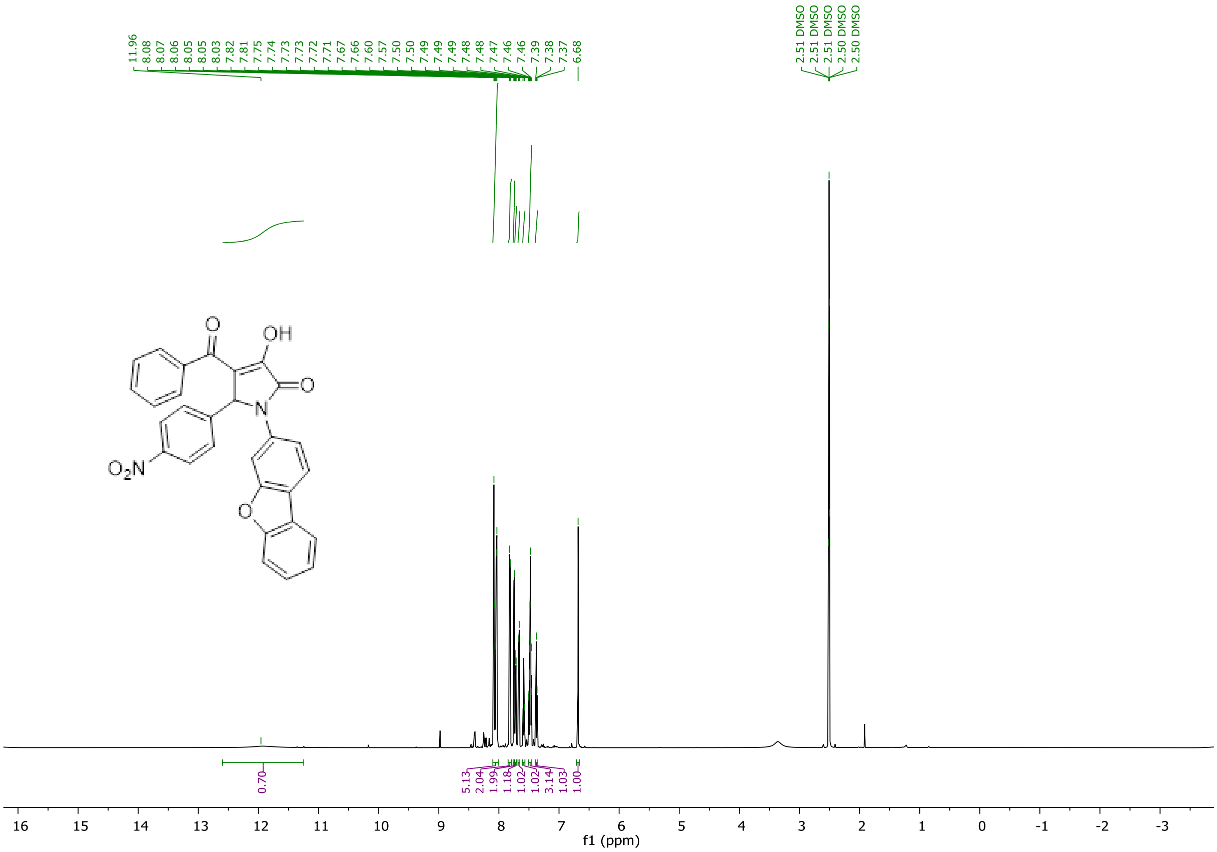

^{13}C NMR of 45 (176 MHz, DMSO-d_6):

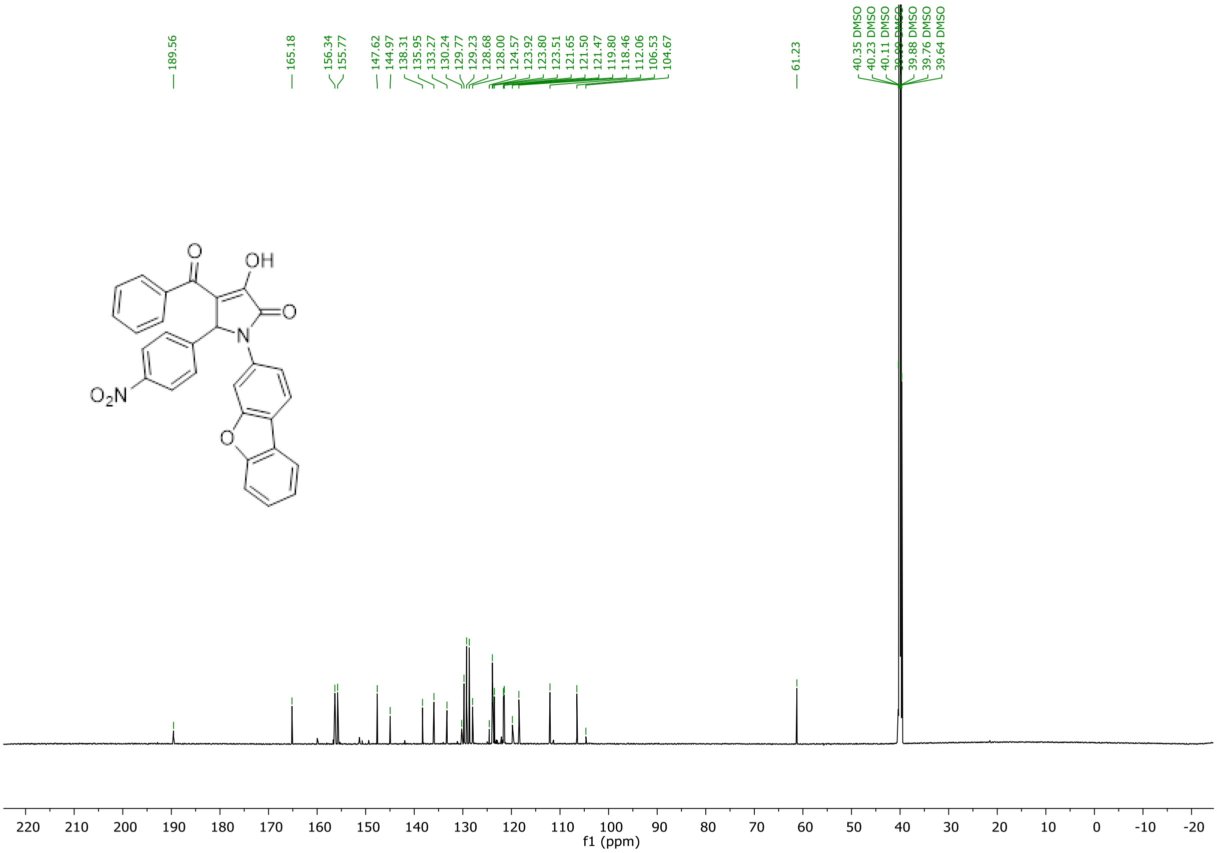

^{1}H NMR of 46 (700 MHz, DMSO-d_6):

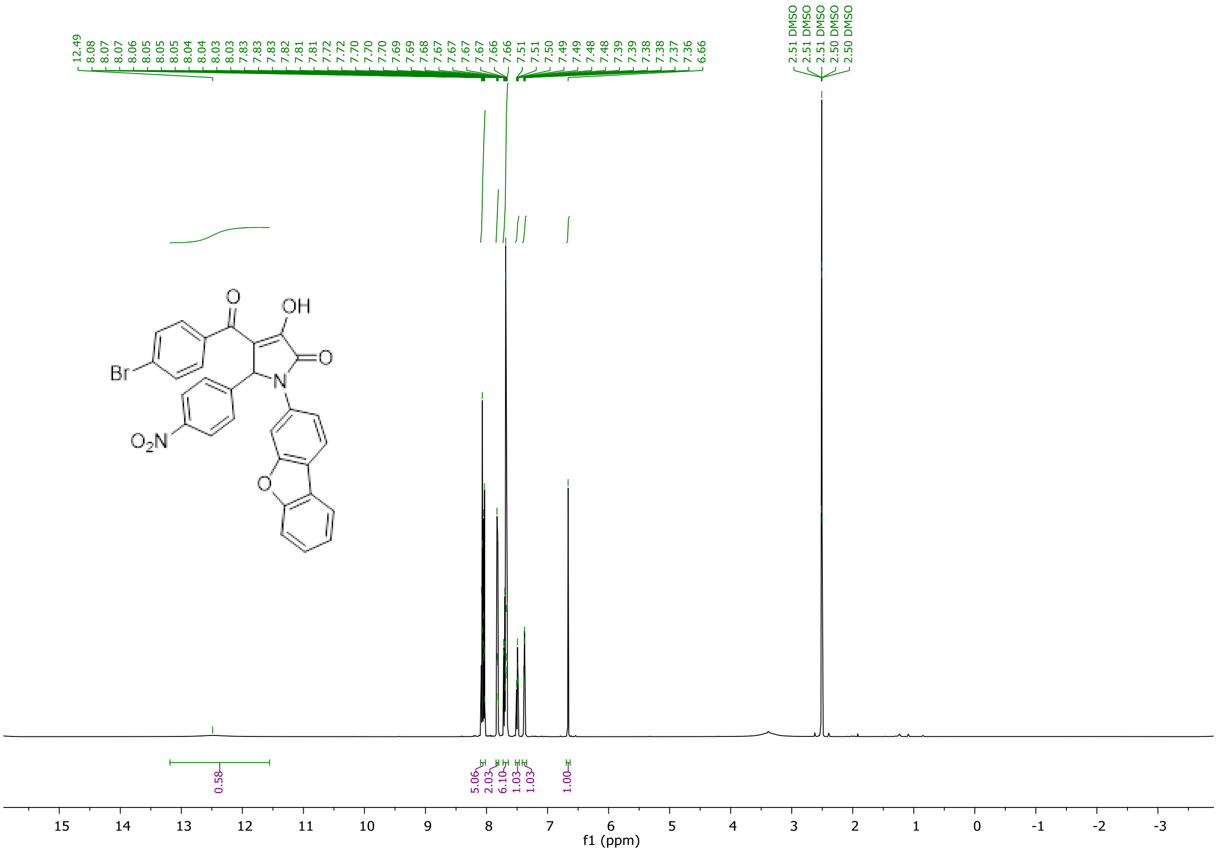

^{13}C NMR of 46 (176 MHz, DMSO-d_6):

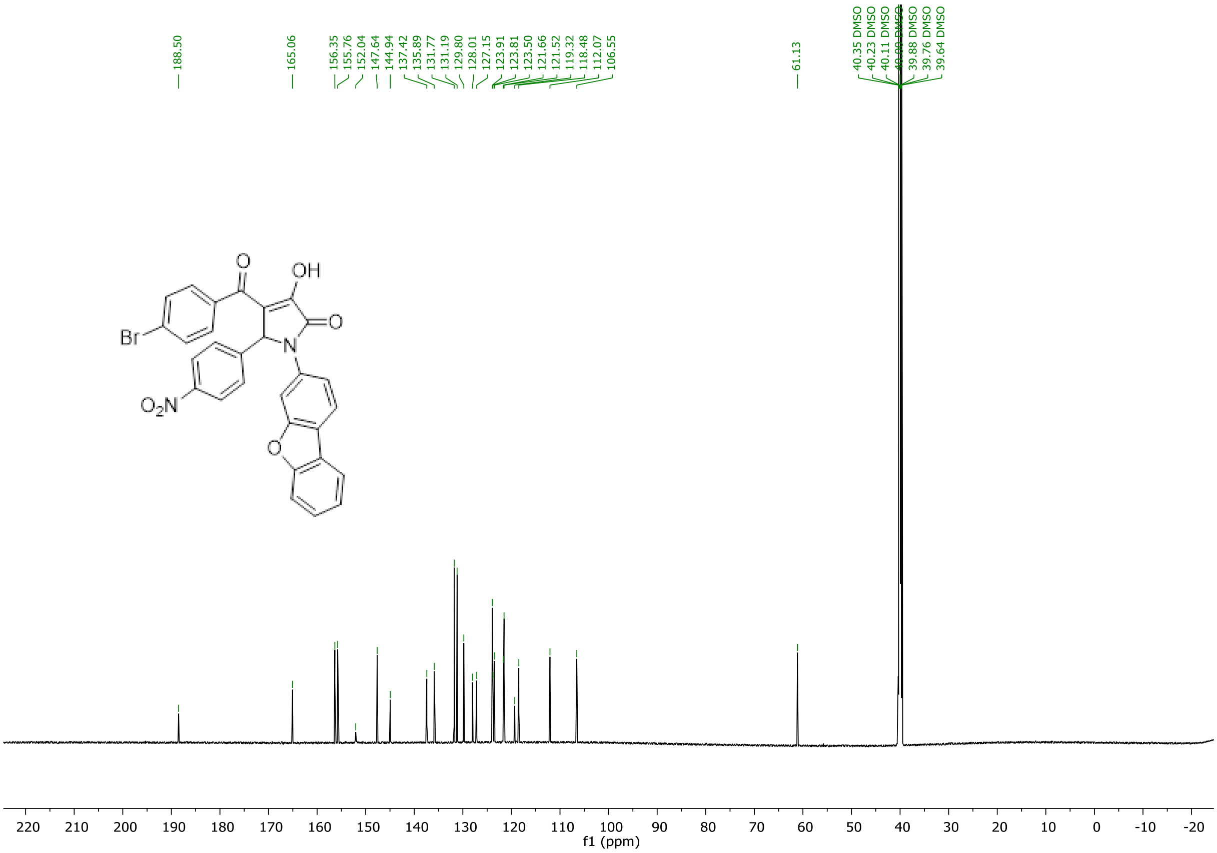

^{1}H NMR of 47 (700 MHz, DMSO-d_6):

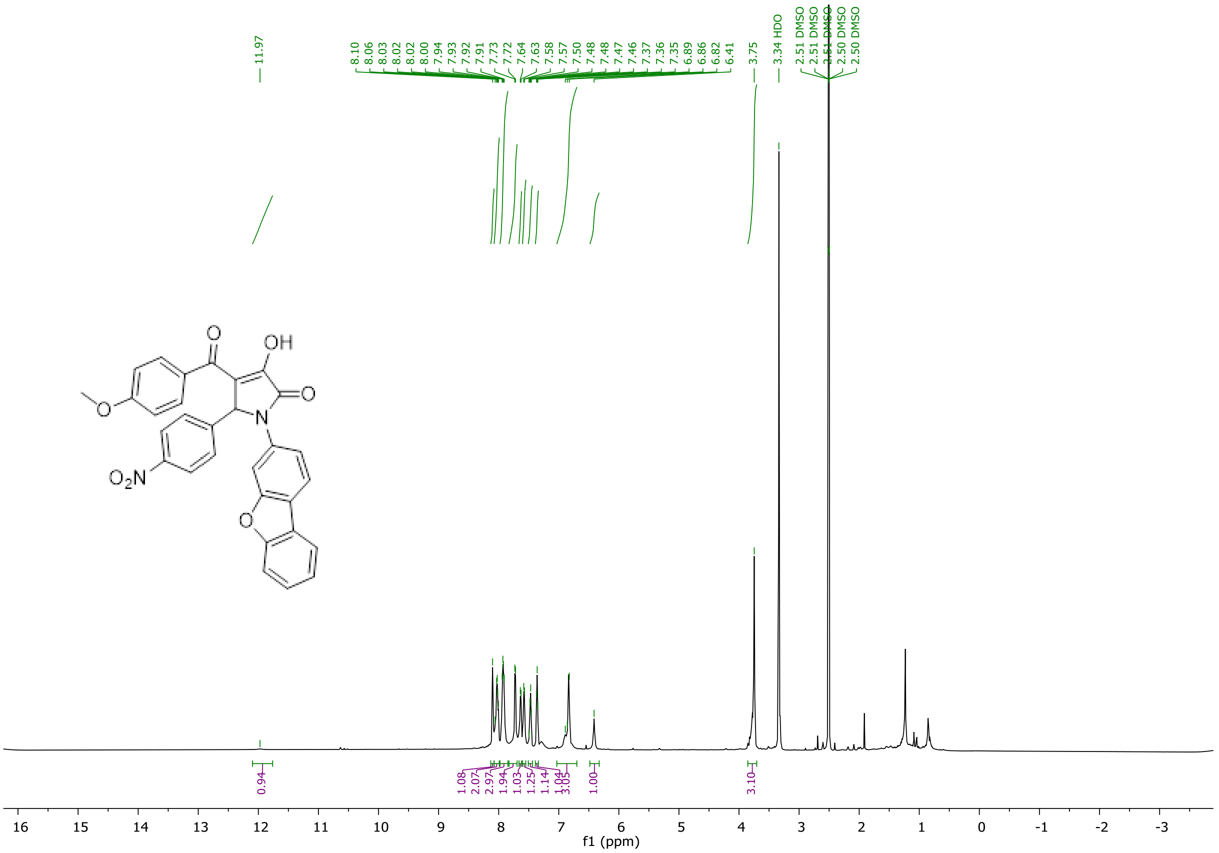

^{13}C NMR of 47 (176 MHz, DMSO-d_6):

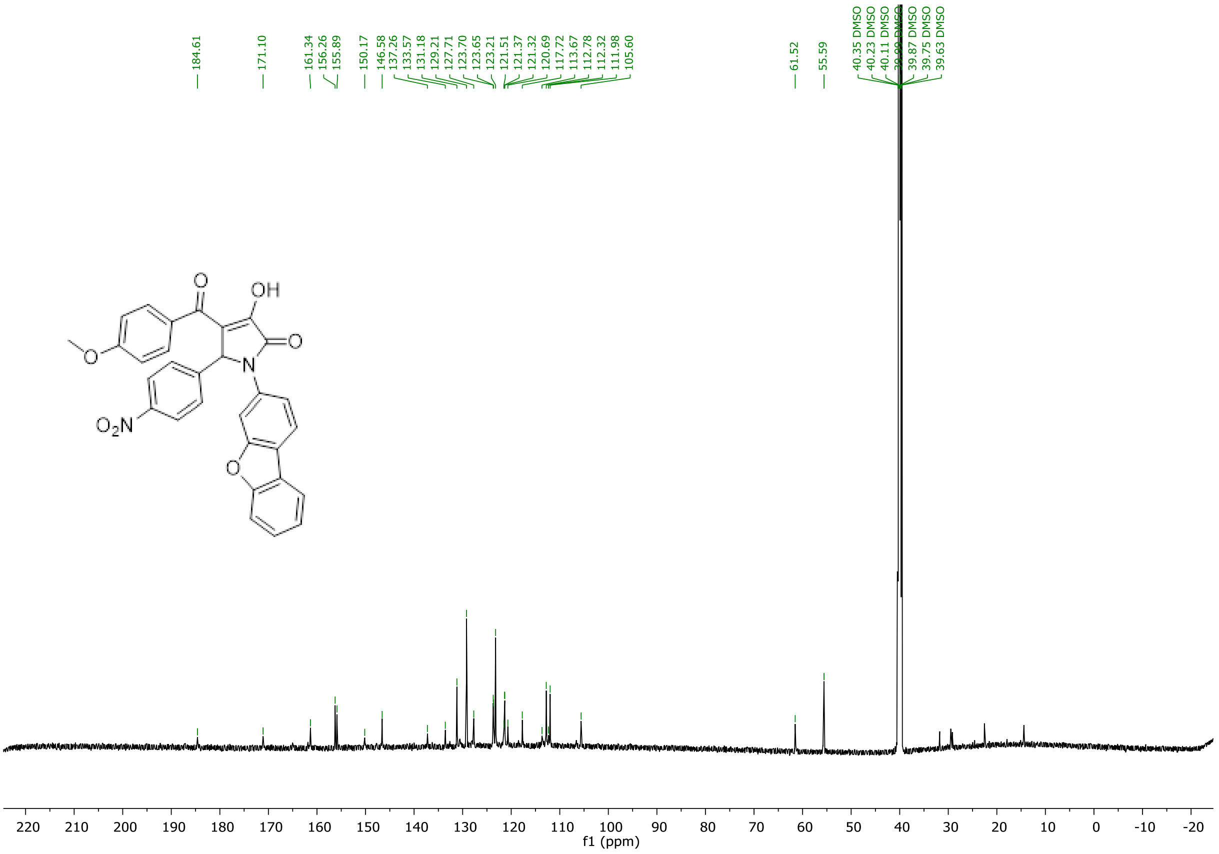

^{1}H NMR of 48 (600 MHz, DMSO-d_6):

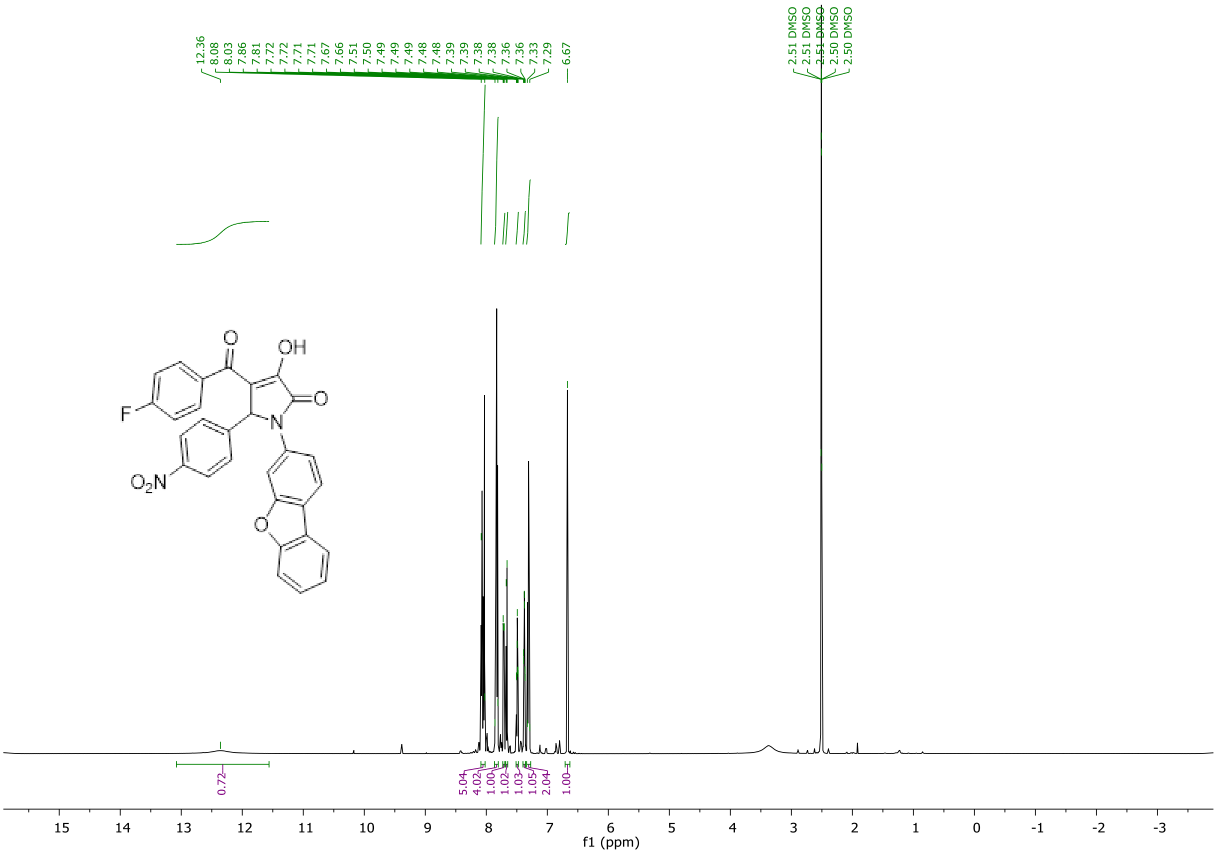

^{13}C NMR of 48 (151 MHz, DMSO-d_6)

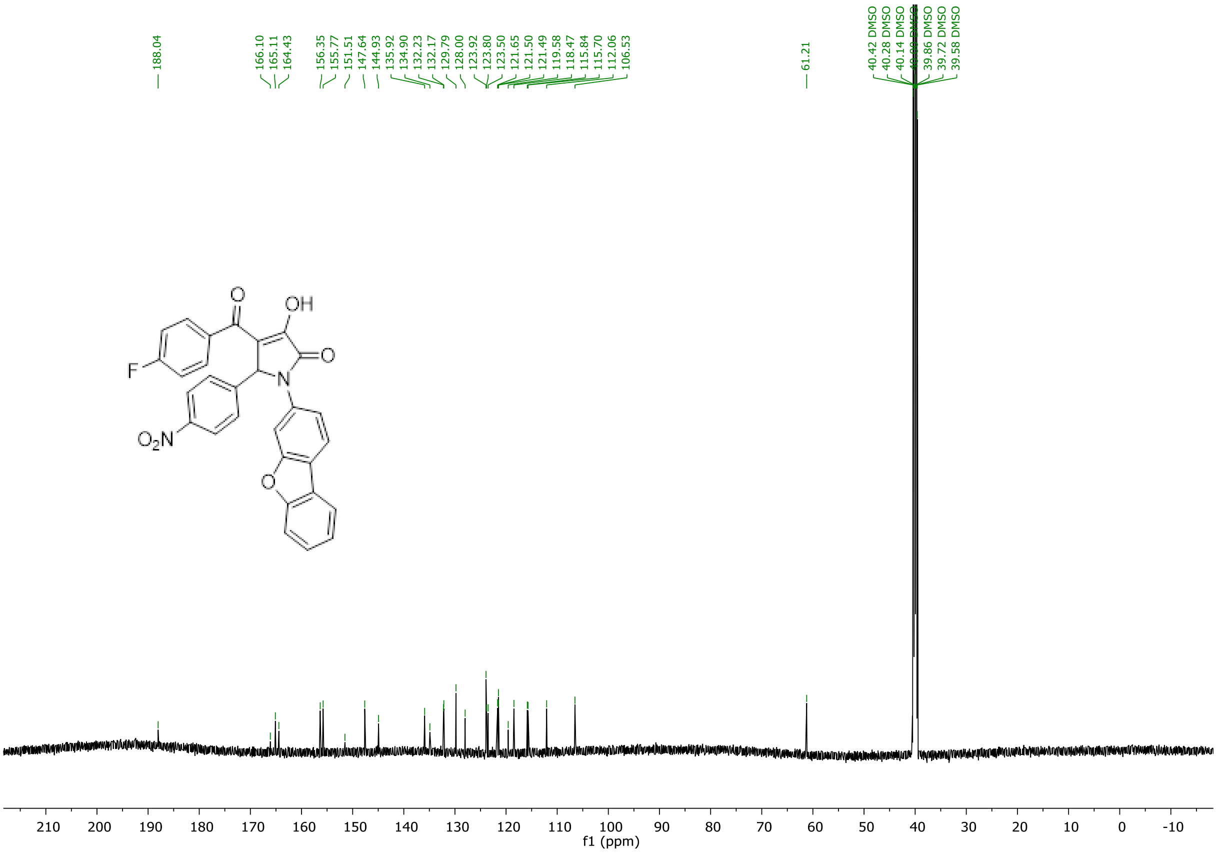

^{1}H NMR of 49 (700 MHz, DMSO-d_6):

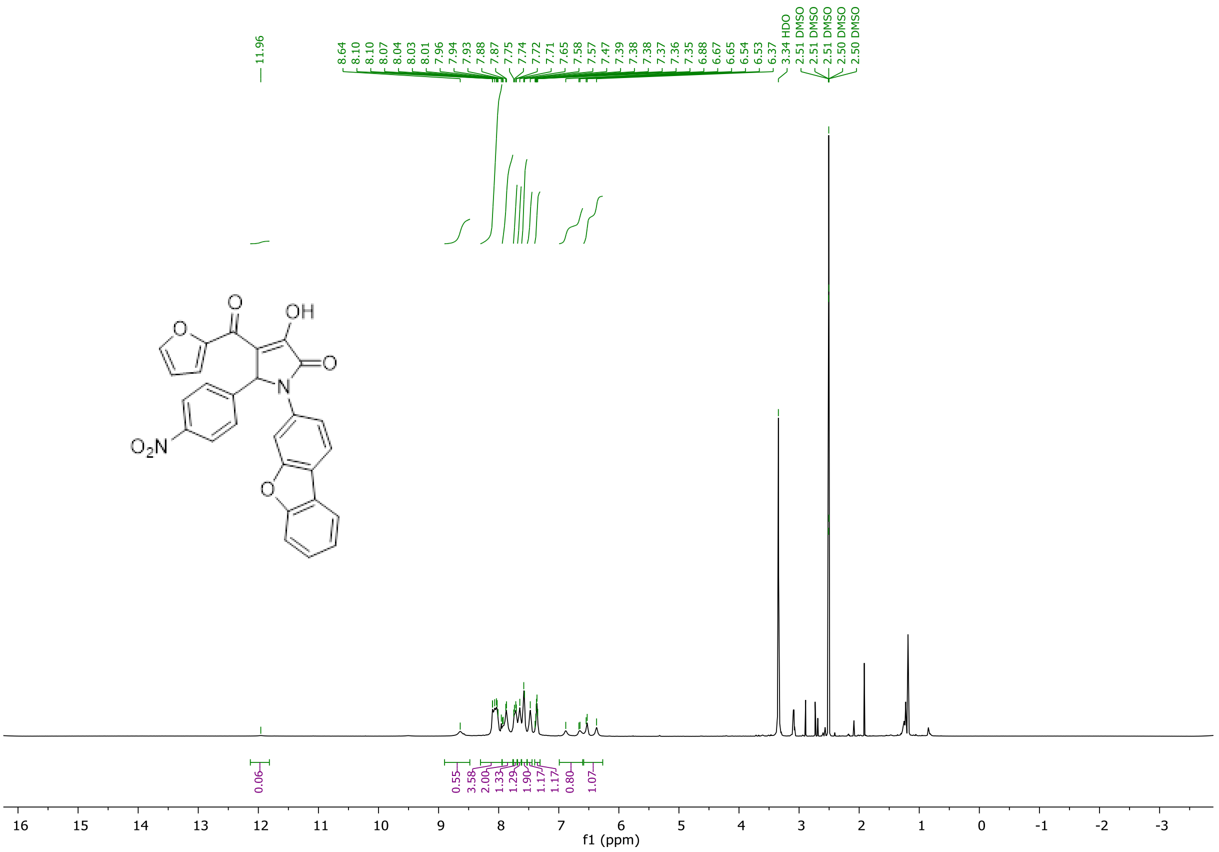

^{13}C NMR of 49 (176 MHz, DMSO-d_6):

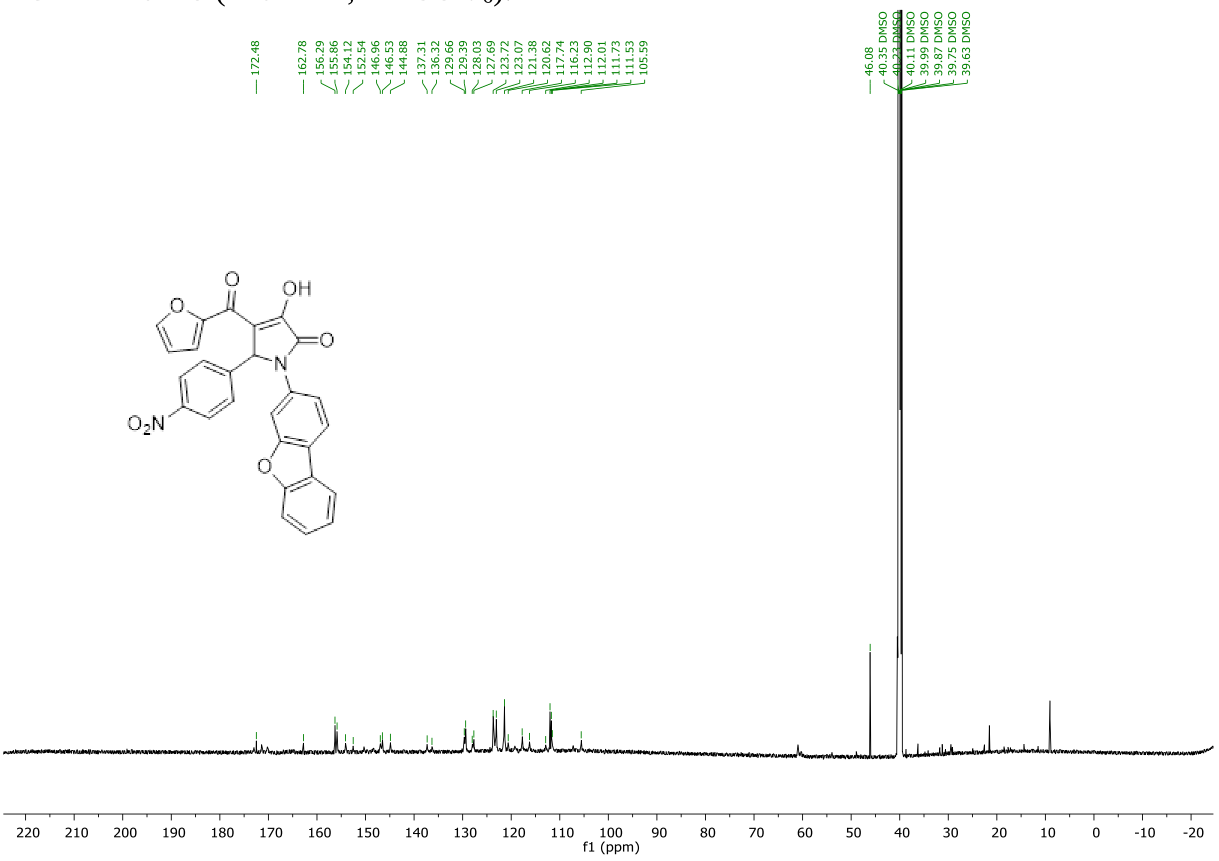

Chiral separation

Chiral separation of **11**: (15 mg, 0.032 mmol) were separated by chiral column chromatography on a Chiralpak IC (250 mm × 20 mm, 5 μm) column. A solution of **11** (30 mg/mL in DCM) was injected and eluted with 40% IPA/DEA 100/20 mM in CO_2, 40 °C, a flow rate of 100 mL/min, and detected at 254 nm. The first eluted compound was collected and evaporated to afford **11*S*** (2.1 mg, 99.3% ee) and the second eluted compound was collected and evaporated to afford **11*R*** (3.3 mg, 87.9% ee).

11*S*:

**Column:**	Chiralpak IH	**Column ID:**	
**Mobile Phase A:**	CO2	**Column Dimension:**	150 * 4.6
**Mobile Phase B:**	IPA/DEA 100/20mM	**Particle Size:**	3
**Gradient:**	40% B, 120 bar	**Injection volume:**	10.00 ul
		**Flow:**	3.5 ml/min
**Temperature:**	40°C	**Wavelength:**	PDA Spectrum PDA 254.0
**Sample Concentration:**	Sample in EtOH	**Vial:**	2:d,3

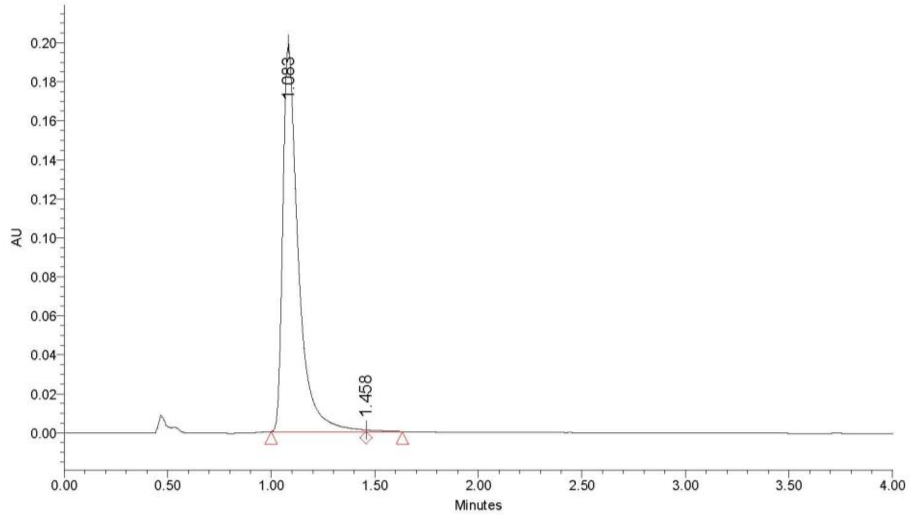

	Retention Time	Area	% Area	k'	N	USP Resolution
1	1.083	1026916	99.65	0.000	1143.3	
2	1.458	3592	0.35	0.346		

ee = 99.3

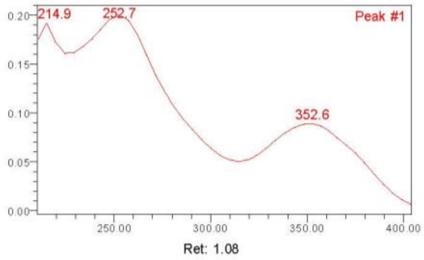

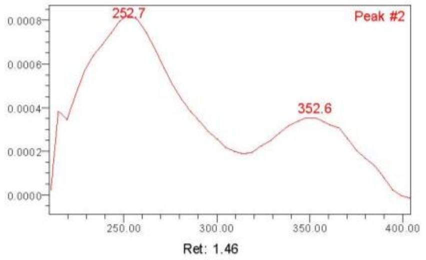

11*R*:

**Column:**	Chiralpak IH	**Column ID:**	
**Mobile Phase A:**	CO2	**Column Dimension:**	150 * 4.6
**Mobile Phase B:**	IPA/DEA 100/20mM	**Particle Size:**	3
**Gradient:**	40% B, 120 bar	**Injection volume:**	10.00 ul
		**Flow:**	3.5 ml/min
**Temperature:**	40°C	**Wavelength:**	PDA Spectrum PDA 254.0
**Sample Concentration:**	Sample in EtOH	**Vial:**	2:D,4

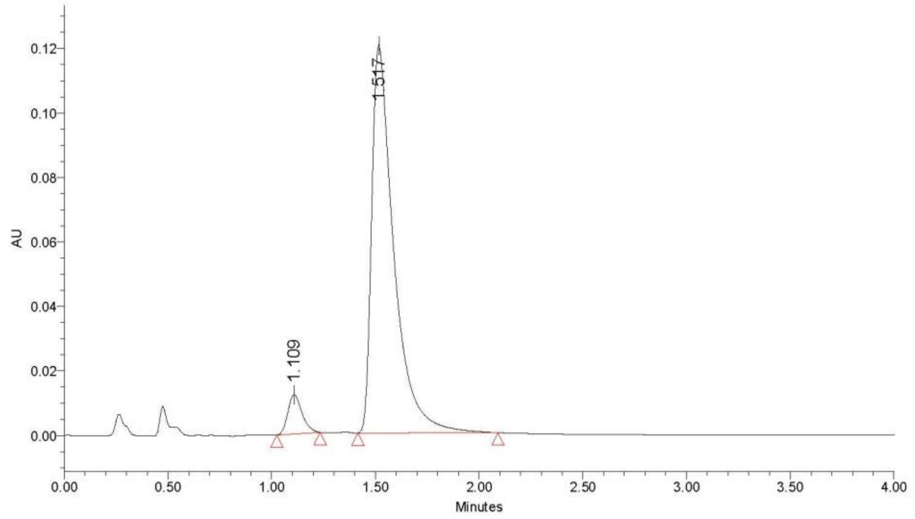

	Retention Time	Area	% Area	k'	N	USP Resolution
1	1.109	57415	6.06	0.000	1312.8	
2	1.517	890157	93.94	0.369	1057.3	2.568

ee = 87.9

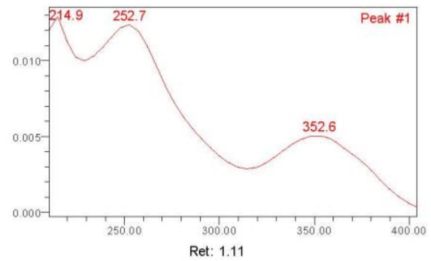

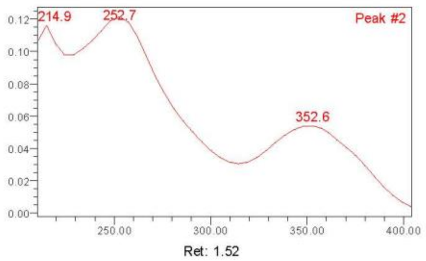

racemic mixture of **11**:

**Column:**	Chiralpak IH	**Column ID:**	
**Mobile Phase A:**	CO2	**Column Dimension:**	150 * 4.6
**Mobile Phase B:**	IPA/DEA 100/20mM	**Particle Size:**	3
**Gradient:**	40% B, 120 bar	**Injection volume:**	5.00 ul
		**Flow:**	3.5 ml/min
**Temperature:**	40°C	**Wavelength:**	PDA Spectrum PDA 254.0
**Sample Concentration:**	Sample in EtOH	**Vial:**	1:d,4

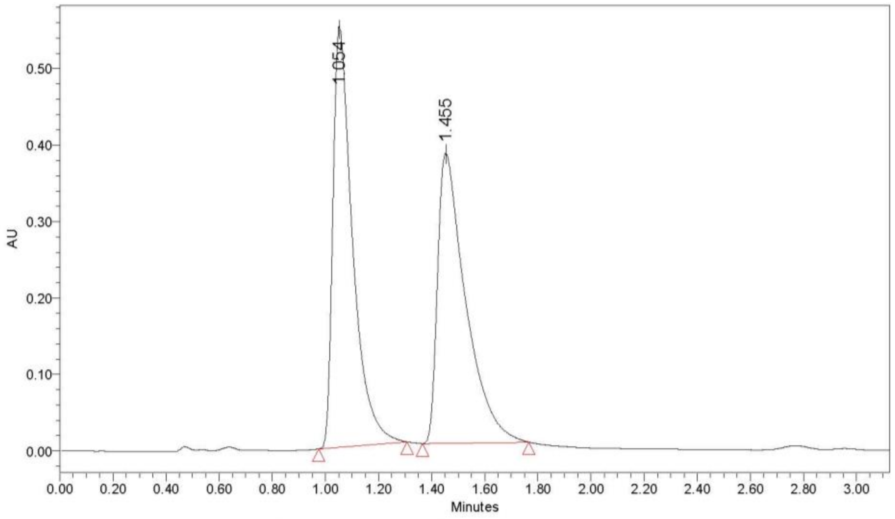

	Retention Time	Area	% Area	k'	N	USP Resolution	Width @ 50%
1	1.054	2833414	50.45	0.000	1010.5		7.537327e-02
2	1.455	2783062	49.55	0.380	910.5	2.331	1.074188e-01

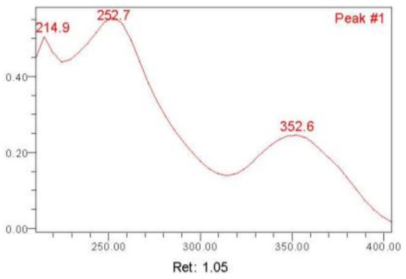

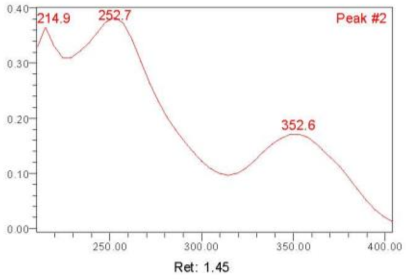

Reference

1. L. Wang, Y. Nam, A. K. Lee, C. Yu, K. Roth, C. Chen, E. M. Ransey, P. Sliz, LIN28 zinc knuckle domain is required and sufficient to induce let-7 oligouridylation. Cell Rep. **18**, 2664–2675. (**2017**)
2. Richter, A., Rose, R., Hedberg, C., Waldmann, H. & Ottmann, C. An optimised small-molecule stabiliser of the 14-3-3-PMA2 protein-protein interaction. *Chem. - A Eur. J.* **18**, 6520–6527 (**2012**).
3. Pallesen J.S., Munier C. C., Bosica, F., Andrei, S. A., Edman, K., Gunnarsson, A., La Sala, G.,Dwichandra Putra, O., Srdanović, S., Wilson, A. J., Wissler, L. Ottmann, C., Perry, M. W. D., O'Mahony, G. Designing selective drug-like molecular glues for the glucocorticoid receptor/14-3-3 protein–protein interaction. *J. Med. Chem.*, **65** (24), 16818–16828 (**2022**).
